# The ‘PhenoBox’, a flexible, automated, open‐source plant phenotyping solution

**DOI:** 10.1111/nph.15129

**Published:** 2018-04-05

**Authors:** Angelika Czedik‐Eysenberg, Sebastian Seitner, Ulrich Güldener, Stefanie Koemeda, Jakub Jez, Martin Colombini, Armin Djamei

**Affiliations:** ^1^ Gregor Mendel Institute (GMI) Austrian Academy of Sciences Vienna BioCenter (VBC) Dr. Bohr‐Gasse 3 1030 Vienna Austria; ^2^ Department of Genome‐oriented Bioinformatics Technische Universität München Wissenschaftszentrum Weihenstephan Freising Germany; ^3^ Vienna Biocenter Core Facilities (VBCF) Dr. Bohr‐Gasse 3 1030 Vienna Austria; ^4^ Workshop, Research Institute of Molecular Pathology (IMP) Vienna BioCenter (VBC) Campus‐Vienna‐Biocenter 1 1030 Vienna Austria

**Keywords:** infection prediction, open source, PhenoBox, PhenoPipe, plant pathogens, plant phenotyping, salt stress, smut fungi

## Abstract

- There is a need for flexible and affordable plant phenotyping solutions for basic research and plant breeding.
- We demonstrate our open source plant imaging and processing solution (‘PhenoBox’/‘PhenoPipe’) and provide construction plans, source code and documentation to rebuild the system. Use of the PhenoBox is exemplified by studying infection of the model grass *Brachypodium distachyon* by the head smut fungus *Ustilago bromivora*, comparing phenotypic responses of maize to infection with a solopathogenic *Ustilago maydis* (corn smut) strain and effector deletion strains, and studying salt stress response in *Nicotiana benthamiana*.
- In *U. bromivora*‐infected grass, phenotypic differences between infected and uninfected plants were detectable weeks before qualitative head smut symptoms. Based on this, we could predict the infection outcome for individual plants with high accuracy. Using a PhenoPipe module for calculation of multi‐dimensional distances from phenotyping data, we observe a time after infection‐dependent impact of *U. maydis* effector deletion strains on phenotypic response in maize. The PhenoBox/PhenoPipe system is able to detect established salt stress responses in *N. benthamiana*.
- We have developed an affordable, automated, open source imaging and data processing solution that can be adapted to various phenotyping applications in plant biology and beyond.

## Introduction

Plant phenotyping techniques allow us to systematically assess plant development and performance under tested conditions and are thus essential assets for plant breeding and research. While phenotyping encompasses all methods that collect data about an organism's observable characteristics, the last few years have seen a boost in the application of high‐throughput phenotyping methods (Fiorani & Schurr, 2013; Yang *et al*., 2013; Fahlgren *et al*., 2015; Rahaman *et al*., 2015). These methods allow for the efficient collection of trait information from large plant populations followed by automated data processing. High‐throughput plant phenotyping is increasingly used in crop breeding (Araus & Cairns, 2014; Ghanem *et al*., 2015; Watanabe *et al*., 2017). It also empowers research approaches such as quantitative trait loci mapping and genome‐wide association study, which aim to close the genotype–phenotype gap by identifying associations between phenotypic traits and genetic markers across a broad panel of genotypes (Slovak *et al*., 2015; Zhang *et al*., 2017). Moreover, computational methods that can identify, classify or quantify plant stress symptoms or predict plant trait outcomes based on phenotypic data collected at an earlier stage have been established (Singh *et al*., 2016). However, the accessibility and affordability of automated high‐throughput phenotyping still lags behind the requirements of the community – a problem termed the ‘phenotypic bottleneck’ (Furbank & Tester, 2011; Cobb *et al*., 2013; Araus & Cairns, 2014).

To date, high‐throughput phenotyping mostly concentrates on very costly large‐scale solutions (e.g. see platforms listed in Rahaman *et al*., 2015: Table 2). Furthermore, phenotyping platforms are often developed and provided by specialized companies where the underlying hardware and software are patent‐protected and can therefore not be freely modified to meet particular research needs without cooperation from the company. By contrast, some labs have established their own phenotyping solutions (Chéné *et al*., 2012; Matos *et al*., 2014; Paulus *et al*., 2014, http://maker.danforthcenter.org). This, however, requires in‐depth knowledge in engineering, electrotechnology and informatics as well as considerable development time. These limitations can be alleviated by providing pre‐developed, openly accessible tools under a general licence, with thorough documentation that includes all technical details enabling its flexible modification. While open source software solutions are available for processing phenotyping data (for an overview of available solutions, see Rahaman *et al*., 2015: Table 3), there is a need for openly accessible phenotyping hardware, ideally integrated with data evaluation pipelines. Such solutions would especially benefit smaller or less well‐funded labs, allowing them to perform phenotyping studies adapted to their specific research questions.

Phenotyping methods have been applied to study plants’ responses to biotic as well as abiotic stresses (Mahlein *et al*., 2012; Barbedo & Garcia, 2013; Li *et al*., 2014). Many abiotic stresses cause systemic or quantitative responses in their host, due to their effects on the regulation of central physiological processes. For example, salinity causes osmotic and ionic stress to plant cells. This leads, as part of the acclimation process, to profound changes in multiple processes (e.g. photosynthesis, cell morphology and gene regulation) that eventually lead to phenotypic effects such as leaf yellowing and general growth delay (Suo *et al*., 2017; Yang & Guo, 2018). By contrast, biotic stress phenotyping often concentrates on the detection and evaluation of localized, qualitative infection symptoms, such as leaf lesions caused by a pathogen (Mahlein *et al*., 2012). However, some pathogens do not initially cause distinctive symptoms in their host upon colonization and our growing understanding of plant defence processes in many pathosystems has highlighted their complex and systemic nature (Dean *et al*., 2012; Imam *et al*., 2016; AbuQamar *et al*., 2017). Therefore, the untargeted, quantitative evaluation of systemic phenotypic traits can give additional insights to the study of biotic plant stress responses, particularly in situations where the evaluation of qualitative infection symptoms is unfeasible or does not provide the necessary resolution.

Head smut fungi, which infect crops such as barley, maize and sugar cane in addition to the model grass *Brachypodium distachyon*, cause distinctive symptoms in the form of spore‐filled sori only in the floral organs of their grass hosts (Laurie *et al*., 2012; Poloni & Schirawski, 2016; Rabe *et al*., 2016). During the host's vegetative phase, the fungal pathogen causes no distinctive symptoms, reminiscent of plant endophytes – organisms that colonize plant tissue without causing apparent disease. In endophytes it has been shown that – despite the lack of explicit infection symptoms – the presence of a second organism within the host and their mutual interaction can lead to distinct changes in the physiology, morphology and growth of the host plant (Latchs & Christensen, 1985; Varma *et al*., 1999; Olejniczak & Lembicz, 2007; Dupont *et al*., 2015; Larriba *et al*., 2015; Rozpadek *et al*., 2015). Stunted growth of head smut‐infected plants has been previously reported (Gallart *et al*., 2009). However, there has been no systematic examination of the potential effects of head smut infection on host growth and morphology before the occurrence of distinct infection symptoms, although this could be of considerable interest in relation to early pathogen detection.

In contrast to the late symptom occurrence in head smuts, the maize‐infecting smut fungus *Ustilago maydis* causes gall formation in infected plant tissues (leaves and floral tissues) a few days after infection. The frequency of establishment and size of these galls are commonly used to score the virulence of *U. maydis* lines. However, it has been shown that *U. maydis* infection also leads to systemic changes in the host beyond local gall formation, including global transcriptional reprogramming (Skibbe *et al*., 2010) and changes in metabolism and photosynthetic activity in otherwise asymptomatic leaves (Horst *et al*., 2010). As with other biotrophic pathogens, *U. maydis* uses an arsenal of secreted molecules – so‐called effectors – to manipulate the host's metabolism to its advantage (Doehlemann *et al*., 2008; Dodds *et al*., 2009; Djamei *et al*., 2011). Interestingly, deletion of effector encoding genes often does not lead to distinct qualitative differences in symptom development as evaluated by classical scoring. Here lies added potential in the application of untargeted whole‐shoot phenotyping, as this may reveal more subtle, quantitative rather than qualitative, differences in host responses to infection by different *U. maydis* genotypes in all of the plant's above‐ground tissue. Thus, it may reveal dimensions of infection responses not covered by classical infection scoring. To summarize, the study of global infection effects on the plant hosts, along with the study of other biotic and abiotic plant stress situations, will thus profit from better accessibility of whole‐shoot phenotyping solutions.

In this study we present a flexible open hardware/open source phenotyping system for the evaluation of visual shoot traits. Our system consists of the ‘PhenoBox’, a chamber that automatically captures and processes plant images from different angles, and the sample management and evaluation framework ‘PhenoPipe’, which submits images for feature extraction and subsequently for statistical analyses through pre‐supplied as well as custom evaluation modules written in the statistical programming language R.

We demonstrate the versatility of our system by providing examples of its use to study two biotic stress situations and one abiotic stress situation in three different plant species: first, we show that we can predict qualitative infection outcome in the model grass *B. distachyon* infected with the head smut fungus *Ustilago bromivora* based on changes in visual features during the early vegetative growth phase, weeks before qualitative symptom development. Second, we used our system to compare quantitative responses in shoot phenotypic traits in maize plants infected with a fully capable *U. maydis* strain and with effector mutant strains. We propose the calculation of multi‐dimensional distances between plant groups based on principal components derived from visual traits as a method to quantitatively compare phenotypic impact between different treatments. Finally, we illustrate the wider applicability of our system to a dicot model and abiotic stress situation by studying the effects of salt stress on *Nicotiana benthamiana*. We show that we can sensitively identify different classes of responses which are in line with published salinity response phenotypes. This experiment is summarized in the form of a tutorial on how to use the PhenoBox/PhenoPipe system. Overall, we expect that our PhenoBox/PhenoPipe system will help fill the gap in affordable, flexible, automated phenotyping solutions and thus open new approaches to study biotic and abiotic stress responses in plants.

## Materials and Methods

### *B. distachyon* growth conditions and infection

*B. distachyon* ecotype ABR4 seeds were germinated as described by Rabe *et al*. (2016). Ideally, the roots were *c*. 5 mm long when the seedlings were infected. *U. bromivora* sporidia of the two mating types were grown in PD medium (24 g l^−1^ potato dextrose broth in deionized H_2_O) to an OD_600_ of *c*. 0.6–1, thoroughly washed with sterile water and resuspended in sterile water to an OD of 1. The two mating types were then combined in a 1 : 1 ratio, and seedlings were submerged in infection solution for 40 min. The tubes were then carefully centrifuged (1200 ***g***, 1 min) and most of the supernatant was removed, so that a low amount of viscous fungal solution was left to moisten the seeds. Seedlings were incubated at *c*. 21°C with the fungal solution for 12–24 h. Seedlings were subsequently planted and vernalized as described by Rabe *et al*. (2016). Plants flowered *c*. 4 wk after vernalization and clear infection symptoms with spore‐filled sori in the spikelets were observable 6 wk after vernalization.

### *Zea mays* growth conditions and infection

*Zea mays* genotype EGB seeds (Olds Seeds, Madison, WI, USA) were potted in a 4 : 1 mixture of standard potting soil (Einheitserde Werkverband e.V., Sinntal‐Altengronau, Germany) and perlite (Granuflor, Vechta, Germany) and plants were grown in a temperature‐controlled glasshouse (14 h : 10 h light : dark cycles; 28°C : 20°C). Plants were infected with *U. maydis* 7 d after potting as previously described (Kamper *et al*., 2006).

### *Nicotiana benthamiana* growth conditions and salt treatment

*Nicotiana benthamiana* was grown at 21°C, 60% humidity under short day conditions (8 h : 16 h, light : dark) using the same soil mixture as described for *Z. mays*. Plants were singled out 1 wk after sowing. Up to 3 wk after singling out, plants were automatically watered (soaked with tap water for 30 min, twice per week). They were then removed from automatic watering and randomly split into a control and treatment group. During the first week of the treatment regime, treated plants were soaked twice with 200 mM NaCl solution in tap water for 2 h, while control plants were soaked with tap water. After a pre‐experiment showed that 200 mM treatment led to no appreciable effects on plant morphology after 10 d, treatment was switched to soaking with 400 mM NaCl solution.

### Lemnatec and IAP feature extraction

For Lemnatec analysis, LemnaGrid, LemnaBase and LemnaMiner, all version 6.14, were used. An image analysis configuration (IAC) was designed based on a randomly chosen data set of 197 images, which represented 12% of the total data set, and contains the following steps: color mean shift, nearest neighbor foreground/background color separation, fill of areas and fill of holes device, region of interest filter, object composition device, and the mean color property tool. Adjustments to parameters was done according to the experimental setup and imaging conditions. After processing and feature extraction, a quality control step was performed on a randomly chosen data sub‐set and the IAC was modified accordingly. The IAC is available as Supporting Information Notes S1. Within the PhenoPipe, Integrated Analysis Platform (IAP) version 2.1.0 is used. For each experiment, a custom segmentation pipeline was first established using IAP on a client computer as described in Notes S5. It is particularly critical to set the background color correctly, compare different background removal algorithms and fine‐tune color filtering limits to achieve good plant segmentation. Lists detailing all image traits extracted by the Lemnatec software and IAP software are given in Notes S2 and S3.

### Phenobox and PhenoPipe

Images detailing the PhenoBox construction and a list of all components can be found in Note S3. A 3D model of the PhenoBox pot adaptor is available in two formats as Figs S5 and S6. The complete source code to run the PhenoBox and PhenoPipe, together with a detailed documentation in wiki format, can be found at https://github.com/Gregor-Mendel-Institute/PhenoBox-System. A user guide describing how to use the PhenoBox and PhenoPipe for an experiment – exemplified by the *N. benthamiana* salt treatment experiment described in the results section – can be found as Notes S5. All resources are available under the terms of the GNU General Public Licence v.2.

### *K*‐means‐based classification of plants and evaluation of classification performance, calculation of multi‐dimensional distances

Statistical analyses within the PhenoPipe were performed in the ‘Renjin’ reimplementation of the R statistical environment for the Java Virtual Machine (http://www.renjin.org). Image features were averaged over all images taken for an individual plant by taking the median value for each feature. Features were then z‐score‐transformed across individuals to adjust for the differences in magnitude between different features. This is conducted for all individuals and features as follows:

$${\text{Feature}}_{{\mathrm{indiv}}_{-}\mathrm{zscore}}=\left({\text{Feature}}_{\mathrm{indiv}}-\text{mean}\left(\text{Feature}\right)\right)/\text{sd}\left(\text{Feature}\right).$$

The z‐score‐transformed image feature traits were used as an input for a principal component analysis (PCA) (R function prcomp()).The first *x* principal components (PCs) that cover ≥ 90% of the total variation in the dataset were selected as input for *k*‐means classification. Based on these variables, classification into two clusters was performed using *k*‐means (R function kmeans()) with 100 random starts. To assess prediction performance for the pathogen solution‐inoculated plants, the predicted outcome for each plant was compared to a list specifying the observed outcome (i.e. symptoms in the spikelet or not). The *R*/*R* ^2^ values and corresponding correlation *P*‐values were thus calculated as the correlation of the predicted and the observed outcome. A confusion table was assembled to count the pathogen solution‐inoculated plants falling into each of four groups: predicted symptoms and observed symptoms = true positive, predicted no symptoms and no symptoms observed = true negative, predicted symptoms and no symptoms observed = false positive, predicted no symptoms and symptoms observed = false negative.

Based on these groups, the following prediction performance measures were calculated:

$$\begin{array}{c} \text{Precision = true positive}/(\text{true positive + false positive})\\ \text{Recall}=\text{true positive}/(\text{true positive + false negative})\\ \text{F1 score}=2\times \text{precision}\times \text{recall}/(\text{precision}+\text{recall}) \end{array}$$

The F_1_ score is a measure of classification accuracy that considers both the recall and the precision of the method. It is the harmonic average of the precision and recall, so that the F_1_ score reaches its best value at 1 (perfect precision and recall) and worst at 0.

Multidimensional distances between treatment groups were calculated based on the PCs as follows: for each PC, the median value for the treatment group was calculated from the individual values of all plants within this treatment group. This then defined the multidimensional median coordinates for each treatment group. The R function dist() was then used to calculate euclidean distances between the median coordinates of the different treatment groups. The complete R code can be found as part of the github repository referred to above.

### Correlation analysis

Spearman correlations between all extracted visual traits were calculated and *P*‐values were corrected by the Benjamini–Hochberg method (Hochberg & Benjamini, 1990). Subsequently, correlations were filtered to retain those with a corrected *P*‐value < 0.05. The correlation matrix was plotted with the pheatmap() function from the R package pheatmap. Statistical analyses that were conducted outside the PhenoPipe were performed in the R statistical environment v.3.4.2.

## Results

### PhenoBox and PhenoPipe – a system to facilitate plant image capture and analysis for phenotyping

**Figure 1:**
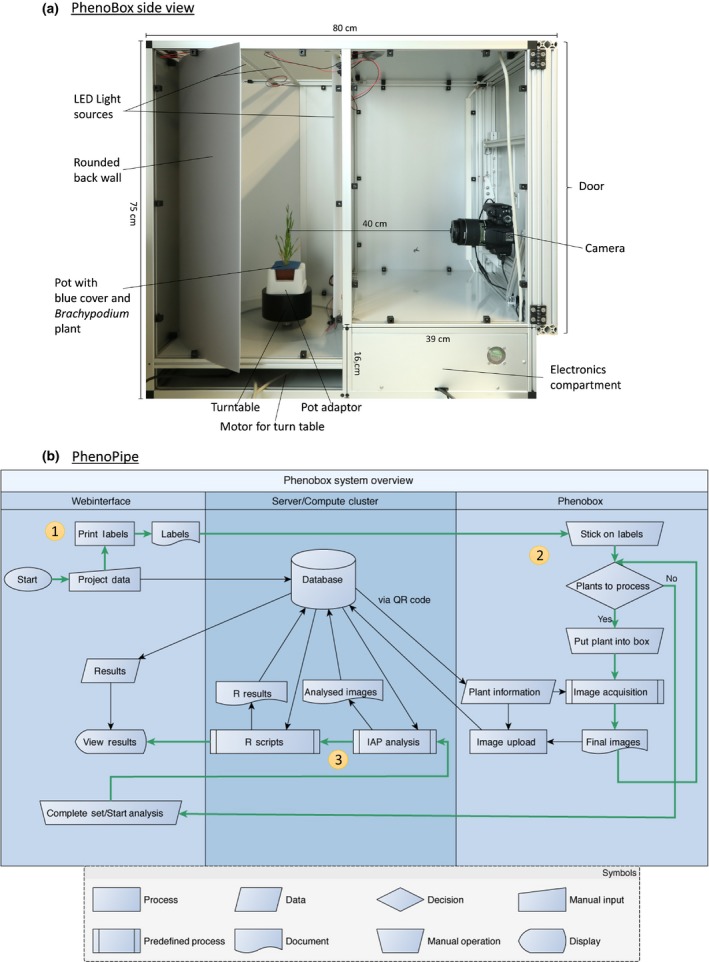
‘PhenoBox’ and processing pipeline ‘PhenoPipe’. (a) Side view of the PhenoBox with one side wall removed. Dimensions are indicated. The camera and turntable are controlled by a Raspberry Pi 3 microcomputer located in the electronics compartment in the lower right area of the PhenoBox. A circuit diagram of the electronic setup as well as further images detailing the setup of the PhenoBox are found in Supporting Information Fig. S5. Pots with plants are covered with blue, air‐permeable foam sheet material for imaging, as this improves segmentation of plants in the images. (b) Flow chart of the automated visual phenotyping pipeline (‘PhenoPipe’). (1) Information about the experiment and individual plants is entered and pot labels with QR codes identifying each plant are printed via the web interface. (2) For imaging, plants are put into the PhenoBox, and pictures are taken, converted to .png format, renamed and uploaded to a network drive. Plant information is requested from the server via the QR codes. (3) After imaging of a plant set is completed, the analysis process can be started. The images are submitted to an Integrated Analysis Platform (IAP) instance for analysis and the extracted image features are passed to R scripts for statistical analysis. The outputs are made available via the web interface.

We have developed an integrated system for plant image capture and subsequent processing of plant images and data evaluation. This system consists of the ‘PhenoBox’ (Fig. 1a), which records plant side view images in an automated fashion, and the encompassing data management and processing pipeline ‘PhenoPipe’ (Fig. 1b). The advantage of our system is that it can perform all analysis steps, from image capture through image processing and segmentation to data preprocessing up to statistical evaluation of sample groups and specific downstream analyses. The hardware is economic to rebuild (material cost *c*. €3000/$3300) with openly available construction plans (Notes S4). The system allows imaging of plants between 1 and 40 cm in height in pots of three sizes: 93, 66 or 50 mm side length (top). The PhenoPipe has a user‐friendly web interface. The software modules are open source (https://github.com/Gregor-Mendel-Institute/PhenoBox-System), use an architecture that allows easy adaptation to individual phenotyping needs and supports running several PhenoBoxes in parallel.

The PhenoPipe data management and processing applications run on a server accessible to the PhenoBox hardware as well as to end users’ client computers. By first defining sample groups, and – within these – individual plants via the PhenoPipe web interface, each plant is given a unique database identity, which is accessed from frontal plant images via a pot label with a unique QR‐Code. Subsequently, the plant is moved by a turntable, so that the PhenoBox can take images from definable angles (e.g. six 60° angled images). The capture of six images from one plant, including QR‐Code decoding, takes *c*. 40 s. The turntable and camera are controlled by a Raspberry Pi 3 microcomputer, which also preprocesses the images and renames them according to the plant identity. Images are uploaded to the server and further processed by the PhenoPipe.

All further image processing and data analysis steps are controlled via the PhenoPipe web interface and are executed on a server. The PhenoPipe uses the openly available IAP software of Klukas *et al*. (2014) for image segmentation and feature extraction. Currently, an image segmentation pipeline for a given image type (e.g. plant species) must be initially established by once running the IAP software manually on a client computer with test images and can then be uploaded to the PhenoPipe. Within the PhenoPipe web interface, users can assign one of the available IAP pipelines to a given dataset and start the image feature extraction task. When image feature extraction is complete, all result files (a table with quantifications of image features for each input image, and images showing the segmentation for each input image) are available for download but can also be directly submitted to further data processing and evaluation steps via the PhenoPipe interface.

The PhenoPipe supports data evaluation modules written in R, which can be assembled into postprocessing stacks, and provides a defined interface for data exchange between individual modules within the postprocessing stack (see Notes S5: PhenoBox system user guide). Standard processing and analysis modules available via the PhenoPipe include a script to filter out irregular and uninformative lines and columns in the IAP output. Furthermore, summarizing image feature information on the level of individual plants and sample groups, the plotting and statistical comparison of image features between sample groups, and PCA of samples is supported. In addition, we have developed and provide downstream functions for plant classification and multidimensional distance calculation analyses used in our application examples. Outputs from the data analysis stacks (data tables and figures) can be downloaded from the PhenoPipe web interface.

### Assessment of PhenoBox system performance

We used test datasets of *B. distachyon* plants to assess the relevance and reliability of the features extracted by our pipeline. Comparison of the features extracted by the openly accessible IAP software run by the PhenoPipe with feature extraction by the established, commercial Lemnatec software suite confirms the high quality of IAP‐based feature extraction, as it can reproduce and even extend results obtained by Lemnatec. In a dataset of *Brachypodium* plants imaged 21 d after removal from vernalization, we extracted 57 relevant features using the IAP‐based pipeline, while we extracted 37 relevant features using a custom defined IAC with LemnaGrid, LemnaBase and LemnaMiner software from Lemnatec. (‘Relevant’ here is defined as variation in feature values between images and that the data for this feature contained < 1/3 missing values. Features for which these criteria were not fulfilled were discarded from further analyses.) Both IAP and Lemnatec extracted features that can be broadly described as belonging to the following categories: geometric features proportional to plant size (e.g. plant height or width), derived geometric features not directly proportional to plant size (e.g. compactness) and pixel‐property‐based features (e.g. average pixel brightness or proportion of pixels falling into a defined color class). However, not only did IAP extract a larger number of features, it also covers a qualitatively distinct subcategory of features – features derived from IAP's leaf skeletonizing algorithm – which was not covered by the Lemnatec feature extraction. This algorithm, which attempts to identify plant leaves on images, can count leaves and determine average leaf length, width and curvature. The results for similar features extracted by both software solutions were very highly correlated (Fig. S1a; Table S1). In general, there is a high degree of correlation within the individual features identified by each method and across the methods (Fig. S2). For example, several of the features are directly related to plant size and form a block of highly intercorrelated features in the top left corner of the correlation matrix (Fig. S2). These observations have implications for data analysis in our PhenoPipe example applications, motivating the use of PCs of image features rather than the features themselves, thus reducing the dataset to a smaller number of linearly uncorrelated variables for downstream applications.

Another aspect that we wanted to assess is how accurately information obtained by our pipeline evaluates shoot growth performance. We thus extracted, at several time points over *B. distachyon* development (12–41 d after planting of germinated seeds), the ‘plant area’ in square pixels from images taken by the PhenoBox, and compared it to the most widely used, albeit destructive, proxy for shoot growth performance, fresh weight (FW). By cutting and weighing individual plants directly after imaging, we show that the feature ‘plant area’ is an effective proxy for FW, as FW and ‘plant area’ had *R* values of almost 0.95 or higher at all tested time points (Fig. S1b). These correlations are similar in strength to the FW–area correlations observed by Klukas *et al*. (2014) when IAP was used to extract visual features from images taken by a Scanalyzer 3D system (LemnaTec). They are also comparable to the correlations between *B. distachyon* dry weight and biovolume calculated from images observed by Poire *et al*. (2014), who also used a Scanalyzer 3D system. When data from all time points are pooled, the strong correlation between FW and area is still observed, albeit with a ‘saturation’ of plant area for plants on the upper end of the tested FW scale compared to those with low biomass (Fig. S1c). This is presumably due to increased leaf overlap in bigger plants. A similar relationship was observed by Klukas *et al*. (2014), who showed that this effect can be corrected for by using a predetermined calibration dataset – a strategy that is thus advisable for the use of the PhenoBox when plants of a wide size range are imaged in one experiment.

### Application of the PhenoBox system to study the quantitative effects of fungal infection in a model grass and predict qualitative symptom development

**Figure 2:**
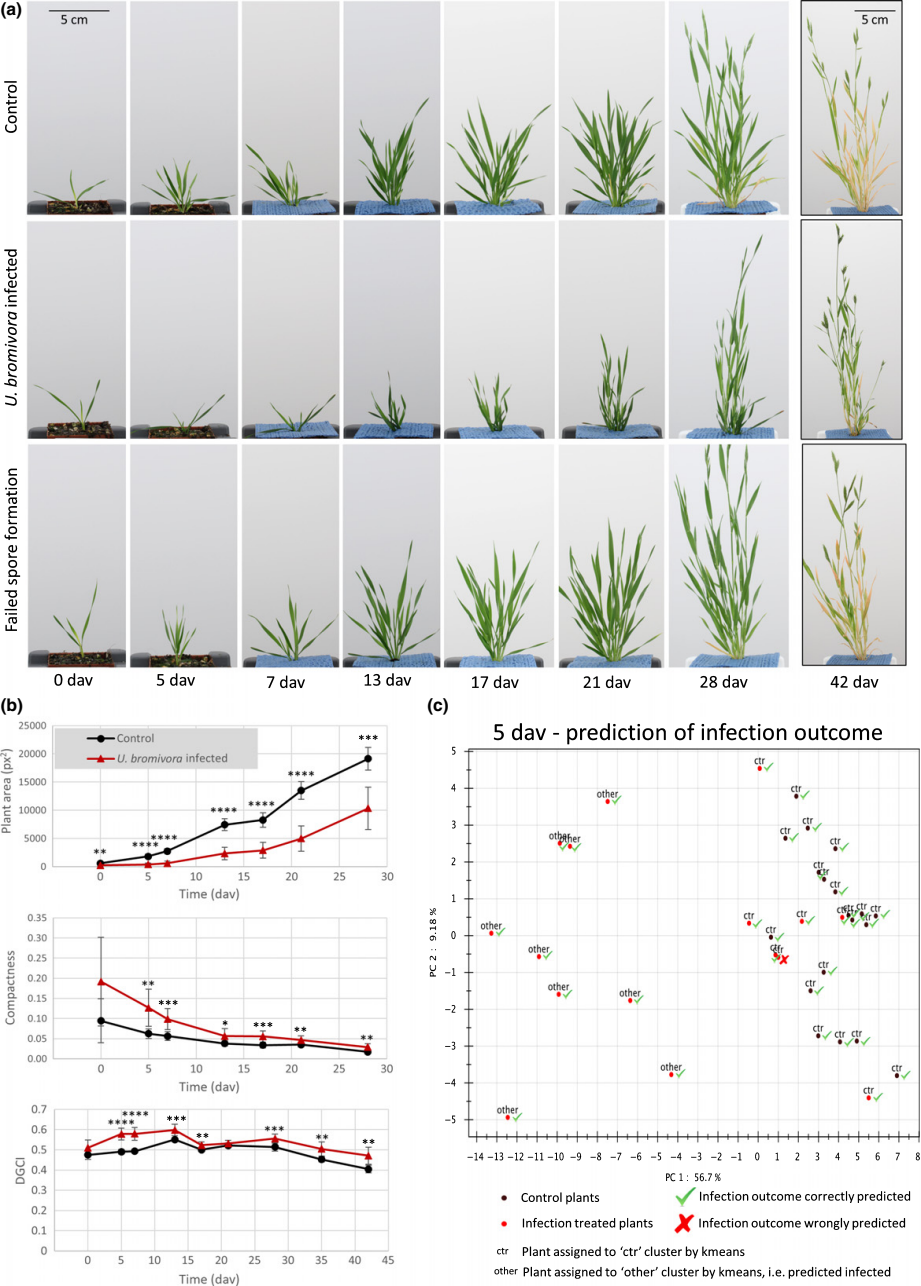
Successfully infected plants differ in growth and morphology from uninfected control plants and plants where fungal spore formation failed. (a) Development of *Brachypodium distachyon* ecotype ABR4, uninfected plants (top line) compared to plants infected by *Ustilago bromivora* at the seedling stage that developed spore‐filled sori in the spikelets (second line) and pathogen‐inoculated plants that did not develop the spikelet symptoms. Images of one typical plant of each group are shown over the time course. The images at 42 d after vernalization (dav) (mature spikelet/full symptom development) were taken at a lower magnification to cover the full plant. (b) Quantification of ‘plant area’ (side.geometry.vis.area), ‘compactness’ (side.geometry.vis.compactness.01) and ‘DGCI’ (side.intensity.hsv.dgci.mean – a numeric indicator of ‘greenness’) from plant images by the Integrated Analysis Platform (IAP) software (Klukas *et al*., 2014). Nineteen control plants were compared to 10 *U. bromivora‐*infected plants where infection was confirmed after symptom development. *, *P *<* *0.05; **, *P *<* *0.01; ***, *P *<* *0.001; ****, *P *<* *0.0001. Error bars indicate standard deviation. Calculation of the compactness and DGCI traits are described in Supporting Information Notes S1. For plant area and compactness, the plots cover only 0–28 dav, as images for 42 dav were taken at a different magnification level. (c) Use of *k*‐means classification to predict infection outcome in *U. bromivora* spore‐treated *B. distachyon* plants based on visual traits. Image features were averaged over all images taken for an individual plant and then z‐score‐transformed across individuals. The z‐score‐transformed image feature traits were used as an input for a principal component analysis. The number of principal components (PCs) to be used as input variables for classification was selected so that the PCs cover ≥ 90% of the variation in the dataset. Based on these variables, classification into two clusters was performed using *k*‐means with 100 random starts (Renjin Java‐based R interpreter). Plants are plotted by their first two PCs. Color of the plotting symbol indicates whether a plant is a control (black) or was pathogen‐inoculated (red). Attached to each plant is a label indicating whether the plant was predicted to belong to the control cluster (‘ctr’) or the infected cluster (‘other’). A green tick mark indicates plants correctly assigned while a red cross indicates plants incorrectly predicted when compared with final infection outcome. This figure is a PhenoPipe output; green tick marks and the red cross were manually added.

As a first example of the use of the PhenoBox system, we studied early infection effects in *B. distachyon* infected by the head smut *U. bromivora*. While characteristic symptoms in the form of black, spore‐filled sori in the spikelets become visible only after flowering, pre‐experiments indicated changes in morphology that already occur before the flowering stage in those plants that go on to develop the spikelet symptoms. We observed a tendency towards slower growth, a less ‘bushy’ morphology and a darker leaf color in the successfully infected plants. To quantify these observations, we used the PhenoBox to image a randomized set of 19 uninfected control plants and 16 plants that had been treated with fungal pathogen suspension. Images were taken at nine timepoints, from the day they were taken out of vernalization (0 dav) up to 42 d after vernalization (42 dav) when plants had already developed mature spikelets (Fig. 2a). Visual inspection of the plants at 42 dav showed that, of the 16 pathogen‐suspension‐treated plants, 10 developed spore‐filled sori in the spikelets, while six developed healthy spikelets (subsequently referred to as ‘failed spore formation’). We then compared the image features extracted by our pipeline between the control plants and those which developed symptoms (Table S2). We identified many features that differed between infected and control plants already during the vegetative phase. Most of these traits showed significant differences immediately after vernalization, indicating that the observed differences were triggered by the fungal infection very early in plant development. We verified that plant size (as determined by plant area on an image) was significantly reduced in infected plants and that infected plants showed a higher compactness, which relates to the ‘less bushy’ phenotype we observed (Fig. 2b). Also, at all timepoints from 5 dav onwards (with the exception of 21 dav), the infected plants had significantly higher DGCI values (a measure of tissue greenness calculated from hue, saturation and brightness) (Fig. 2b). As visible at the 42 dav timepoint in Fig. 2(a), it is apparent that aging plants remained green longer when they were infected, indicating delayed senescence. We also compared the visual traits between pathogen‐inoculated plants that developed spore‐filled sori in the spikelets and those with failed spore formation. Notably, we found that traits that differed between successfully infected and control plants were, in most cases, also significantly different between successfully infected plants and plants with failed spore formation. The latter were generally indistinguishable from noninfected control plants based on their visual traits (Fig. S3; Table S2).

The observation that image features were generally similar between control plants and inoculated plants that did not develop spore‐filled sori, while numerous visual traits differ already during the vegetative phase in successfully infected plants, indicates that these visual traits may be able to predict the infection outcome before the onset of qualitative symptoms in the spikelets. We thus tested if it is possible to use *k*‐means classification (Selim & Ismail, 1984) to split plants into two clusters according to their infection outcome based on visual features: ideally, one of the clusters should be composed of control plants and plants that will not develop qualitative infection symptoms, while the other cluster should be composed of successfully infected plants. Although *k*‐means, as an unsupervised method, is not prone to overfitting, the substantial degree of intercorrelation between the features can lead to suboptimal over‐emphasis of certain aspects of the dataset. Therefore, we performed PCA on the normalized feature values from each imaging time point before *k*‐means classification, and then used as many PCs for prediction as required to cover ≥ 90% of the dataset variation. This typically allowed us to reduce the predictor variables in the datasets from 61 features extracted by IAP down to only between six and 10 derived PCs. Using these PCs derived from each imaging time point as an input, we blindly split the plants into two clusters. We then tested which of the resulting clusters contain more control plants, defining this cluster as the ‘control’ cluster and assigned the pathogen‐inoculated plants that fall into this cluster as ‘failed spore formation’ and those in the other cluster as ‘infected’.

Prediction performance based on the visual features extracted for each imaging time point was evaluated using the final qualitative infection outcome of the tested plants after flowering. We assessed how many of the successfully infected and failed spore formation plants were correctly classified, and, based on this information, calculated performance statistics for each of the analyzed time points (Table 1; Figs 2c, S4). Correlation of the predicted infection status of each plant with their observed outcome shows that a significantly better than random classification performance was achieved from the earliest time points onwards (0 dav). By 5 dav, this method achieved a highly accurate prediction of infection outcome, with only one of 16 plants incorrectly classified (correlation between predicted and observed infection outcome: 0.88) (Fig. 2c; Table 1). This shows that it is possible to use phenotyping of quantitative infection symptoms during the early, vegetative plant stage to assay spikelet infection outcome weeks before qualitative symptoms become visible at flowering. The *k*‐means‐based classification method is available in a postprocessing module via the github repository. It generates a prediction plot such as shown in Fig. 2(c) and a list specifying to which cluster each input plant has been assigned.

**Table 1:** Prediction of infection outcome from visual traits at different time points during *Brachypodium* development

Metadata			Prediction			Prediction statistics for I vs F					
Time point	Imaging	Feature extraction	Correct I	Correct F	Correct C	R obs vs pred	*R* ^2^	*P*‐value	Precision	Recall	F1
0 dav	Phenobox	IAP	6/9	6/6	17/18	0.67	0.44	6.64E‐03	1	0.67	0.80
5 dav	Phenobox	IAP	9/10	6/6	19/19	0.88	0.77	7.64E‐06	1	0.90	0.95
7 dav	Phenobox	IAP	9/10	6/6	19/19	0.88	0.77	7.64E‐06	1	0.90	0.95
13 dav	Phenobox	IAP	9/10	6/6	19/19	0.88	0.77	7.64E‐06	1	0.90	0.95
17 dav	Phenobox	IAP	9/10	6/6	19/19	0.88	0.77	7.64E‐06	1	0.90	0.95
21 dav	Phenobox	IAP	8/10	6/6	19/19	0.77	0.60	4.26E‐04	1	0.80	0.89
28 dav	Phenobox	IAP	9/10	6/6	19/19	0.88	0.77	7.64E‐06	1	0.90	0.95
35 dav	Phenobox	IAP	9/10	6/6	19/19	0.88	0.77	7.64E‐06	1	0.90	0.95
42 dav	Phenobox	IAP	10/10	6/6	19/19	1	1	0	1	1	1

Images were taken at the indicated time points and visual features extracted by Integrated Analysis Platform (IAP) software. Images were acquired using the PhenoBox. The ‘Prediction’ section indicates how many of the plants that showed infection symptoms in their spikelets after flowering (‘I’ for ‘infected’), that were pathogen‐inoculated but did not develop symptoms (‘F’ for ‘failed’) and controls were correctly identified by the *k*‐means clustering‐based prediction method (see Fig. 2, the previous page's second paragraph, and the ‘Materials and Methods’ section). The ‘Prediction statistics’ section shows prediction performance statistics regarding classification of the pathogen solution‐inoculated plants into infected and failed. ‘R obs. vs pred’ is the correlation coefficient if the vector of observed infection outcome for each plant is correlated against the vector of predicted infection outcome, including the *R* ^2^ (coefficient of determination) and *P*‐value for this correlation. Precision, recall and F1 were calculated based on a confusion matrix (see the Materials and Methods section). Precision = true positive/(true positive + false positive), recall = true positive/(true positive + false negative), F1 = 2 × precision × recall/(precision + recall). dav, Days after vernalization.

### Application of the PhenoBox system to quantify pathogen effects in *Z. mays* infection by *U. maydis* effector deletion strains

In the second application example, we used the PhenoBox and PhenoPipe to assess and quantify differences in infection responses between maize plants infected with a *U. maydis* strain containing the full effector repertoire (strain SG200; Kamper *et al*., 2006) and plants infected with strains where individual effector genes were deleted in the same background. SG200 is an engineered solopathogenic strain, that is, it contains components of both *U. maydis* mating types, so that it can infect plants without prior mating. For this assessment, we used the SG200Δ*tin2* mutant strain, which has been described to lack anthocyanin accumulation in infected leaves (Tanaka *et al*., 2014) and the SG200Δ*UMAG_03650* mutant, described to cause a reduction in infection symptoms in floral tissues but no change in symptom development in leaves (Schilling *et al*., 2014). We imaged 12–13 plants for each of four treatments, infected with one or the other effector mutant, with the SG200 progenitor strain, and mock infected with water. Images were taken at three time points: directly before infection, during the early infection stage 4 d post‐infection (dpi), and when infection symptoms were fully established 13 dpi. The data were further processed and evaluated using the established PhenoPipe modules.

**Figure 3:**
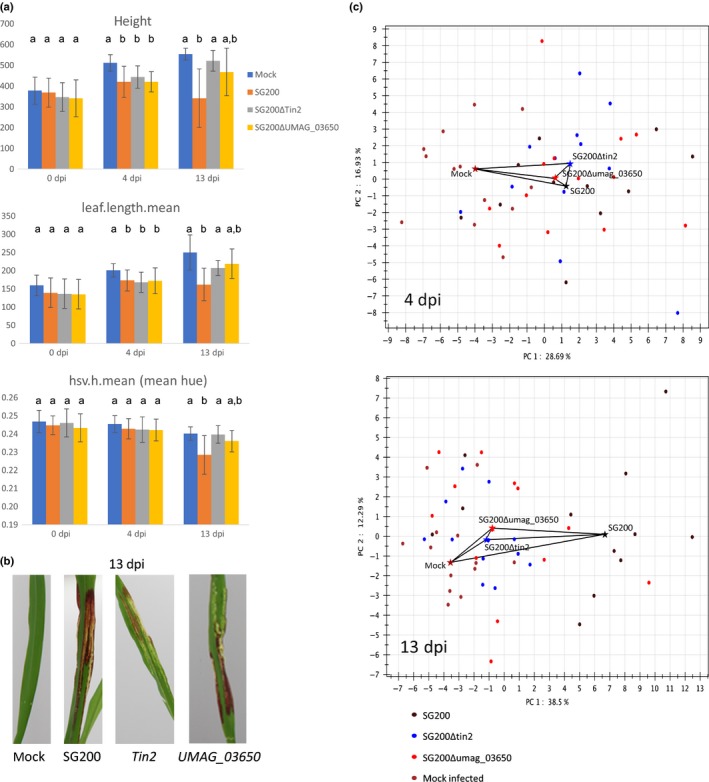
Comparison of phenotypic responses of maize to infection with fully capable *Ustilago maydis* strain SG200 and two effector deletion strains. (a) Comparison of four phenotypic traits extracted from images by our pipeline before infection (0 d post‐infection, dpi) and 4 dpi and 13 dpi with either the SG200 solopathogenic strain, which contains the full effector repertoire, or deletion of either *Tin2* or *UMAG_03650* in the SG200 background. Mock: injection of water instead of infection solution. Error bars indicate the standard deviation. Different letters indicate statistically significant differences between genotypes (*P* < 0.05). (b) Reduced anthocyanin accumulation at 13 dpi upon infection with the *Tin2* deletion strain compared to SG200 and *UMAG_03650* deletion strain. (c) Quantification of the phenotypic effect of infection with the different strains on the host by calculation of multidimensional distances based on recorded phenotypic traits (see the Materials and Methods section). Dots indicate individual phenotyped plants, stars indicate the median position of the plants of a given group along the first two principal components (PCs). Black lines show the projection of the euclidean distances between the median vectors of the groups into the first two PC dimensions. Figures are PhenoPipe outputs with colored stars and labels added manually.

Statistical comparison of the treatment groups showed that, as expected, there were no significant differences between groups before infection (Table S3; Fig. 3). During the early infection stage (4 dpi), plants infected by all three fungal genotypes showed significant differences from the mock infected control plants in some visual traits. These include traits proportional to plant size, such as plant height (Fig. 3; Table S3), but also other morphological traits such as leaf length, leaf curvature and compactness‐related traits. However, there were no significant differences between those plants infected with the progenitor strain SG200 and those infected with either the *Tin2* or the *UMAG_03650* deletion strain. By contrast, at the late infection stage (13 dpi) we observed significant differences in several traits between the *Tin2*‐infected plants and the SG200‐infected plants. These contained size proportional traits and leaf length, but also color‐related traits, for example hsv.h.mean (mean hue), which is in line with the previously described *Tin2* phenotype of reduced anthocyanin accumulation (Fig. 3a,b). Interestingly, at the 13 dpi time point, only plants infected with SG200, but not those infected with the two deletion strains, showed significant differences from the mock inoculated plants. SG200‐infected plants were clearly smaller and differed in their leaf length and curvature, which is presumably in part due to a greater tendency of gall‐bearing SG200‐infected leaves to flex or break off at this stage (even leading to lower absolute plant height measures compared to 4 dpi, Fig. 3). There were also differences between SG200‐ and mock‐treated plants in several color‐related traits, which is in line with the considerable anthocyanin accumulation in the SG200‐infected leaves by that time point (Fig. 3a,b; Table S3). Visual traits in the plant groups infected by the two deletion strains did not significantly differ from the mock infected plants, confirming that infection symptoms in the respective strains were reduced due to the deletion of virulence factors. Nonetheless, traits in the two effector‐mutant‐infected groups typically showed intermediate values between mock‐treated plants and SG200‐infected plants (Fig. 3).

However, analysis of differences in individual traits does not give us a global measure of the difference in the effects of infection between the treatment groups at different time points. To obtain a measure of overall phenotypic distance between the groups, we used the PCA function of the PhenoPipe and developed an additional function, which, based on the PC coordinates determined for each plant, calculates median coordinates for each PC and group and then calculates the distances between the multidimensional median coordinate vectors of all groups. This analysis underlines the impression obtained from comparing individual visual features: that is, during early infection all three groups of infected plants are quite similar to each other but clearly differ from the mock‐treated control plants, while the largest distances observed in the late infection stage are between SG200 and all other treatment groups (Table S3; Fig. 3c). Although the distances between the mock‐treated plants and the *Tin2*‐ and *UMAG_03650*‐infected plants are much smaller, it nevertheless becomes clear that the two groups infected with the deletion mutants are closer to SG200 than the mock group is to SG200. While the use of the distances between sample groups is descriptive in this example, independent experimental replications would make it possible to compare the distances between groups statistically. Thus, this method may be a means to assess overall severity of infection response in different genotypes, treatments or infection stages based on visual traits.

### Application of the PhenoBox system to characterize responses to salt stress in *N. benthamiana*

**Figure 4:**
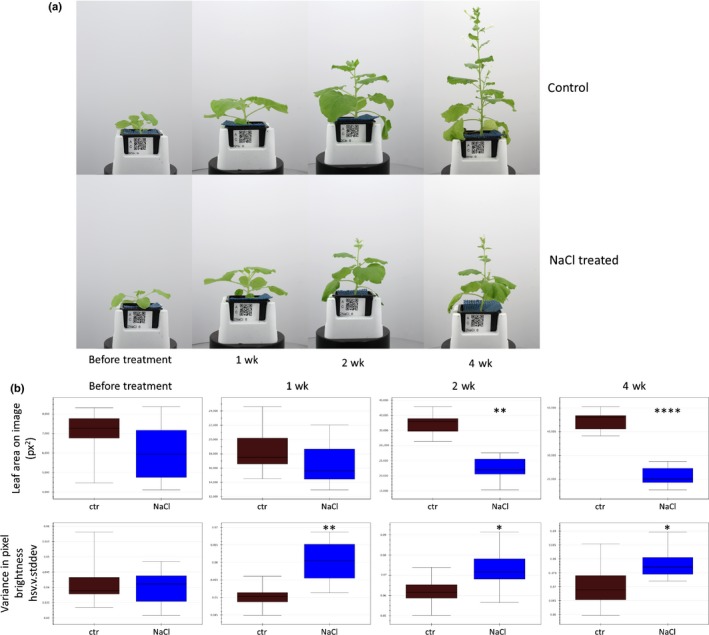
Phenotypic effects of NaCl stress on *Nicotiana benthamiana*. (a) Representative images of time points before and after NaCl treatment taken by the PhenoBox. From the start of treatment onwards, plants were watered with either NaCl solution (see the Materials and Methods section) or water. (b) Alterations in phenotypic parameters observed upon NaCl treatment. *, *P *<* *0.05; **, *P *<* *0.01; ****, *P *<* *0.0001. Error bars indicate the standard deviation. A detailed explanation of traits is found in Supporting Information Notes S1.

In the third application example, we tested the performance of the PhenoBox and PhenoPipe to evaluate phenotypic responses to an abiotic stress situation in a dicotyledonous plant, assaying salt stress response in *N. benthamiana*. Beginning 3 wk after planting, the 11 plants in the treatment group were watered with NaCl solution while 11 control plants were watered with H_2_O. Generally, through to the end of the experiment (4 wk after the first treatment), the only obvious phenotypic response observable by visual inspection was reduced plant size in the NaCl‐treated plants (Fig. 4a). Plants were imaged once before the first treatment, and at three time points after treatment. We used these data to characterize how long after the start of the treatment growth reduction can first be detected and to analyze if the visual imaging approach allows for the identification of other, subtler phenotypic differences. For this experiment, we have included a user guide, detailing the steps of image capture and analysis via the PhenoBox and PhenoPipe, tuning of IAP pipelines and upload via the PhenoPipe interface (Notes S5).

As expected, we did not observe any significant differences between the control group and treatment group before the start of NaCl treatment (Fig. 4b). A significant reduction in plant size‐related parameters was undetectable 1 wk after the start of the treatment but was detectable from 2 wk onwards (Fig. 4b; Table S4). However, analysis of the 1‐wk time point revealed that some color‐related traits differ significantly between the treated and control plants. Specifically, there was a different distribution of pixel brightness values (‘side.intensity.vis.hsv.v.skewness’/‘side.intensity.vis.lab.l.skewness’) and greater variation in pixel brightness values (‘side.intensity.vis.hsv.v.stddev’/'side.intensity.vis.lab.l.stddev’) in the NaCl‐treated plants compared to control plants (Fig. 4b; Table S4). These alterations were undetectable to the human eye, even after closer, retrospective inspection of the images. The differences in brightness‐related traits persist at 2 and 4 wk, with differences in additional color‐related traits at these time points (e.g. ‘hsv.h.mean’, the mean pixel hue value). This application exemplifies the wide applicability of the PhenoBox to different plant species and stress situations and demonstrates the use of this system to detect alterations not observable by eye.

## Discussion

### The PhenoBox/PhenoPipe system

Plant phenotyping is a rapidly developing field and will be critical in understanding genotype–environment interactions as well as the function of individual genes. Its application, however, is still limited by the cost and availability of commercial phenotyping platforms. In this study we present the PhenoBox/PhenoPipe system, a phenotyping solution for the evaluation of visual traits from plant shoot images. We believe that our system is particularly suited to provide easier accessibility of phenotyping solutions in plant research for the following reasons: its excellent cost : performance ratio; the open source/open hardware release format; its flexible architecture, which integrates all steps of the phenotyping process from treatment group definition up to statistical evaluation of image feature data and sample classification; and the availability of a user‐friendly web interface and extensive documentation.

The material cost to rebuild the PhenoBox based on our documentation is very economic at *c*. €3000. However, even an affordable solution is only worth its money if its technical capacity fulfills the needs of the research community to provide biologically meaningful data. We have shown that plant side area extracted from images taken by the PhenoBox system over a series of time points during *B. distachyon* development correlates very highly to plant FW, thus making the area on the image a highly effective and noninvasive proxy for FW, a common read out of plant growth performance. The correlation achieved by our system was similar in strength to published results from maize images taken by the Scanalyzer 3D imaging system installed at the IPK Gatersleben (Klukas *et al*., 2014), or the correlation between digital biovolume and DW in *Brachypodium* described by Poire *et al*. (2014), where images were also taken by a Scanalyzer 3D system. Further, we could also show in the application examples that visual traits obtained by the PhenoBox provided relevant information to study plant stresses, for example allowing the prediction of spikelet symptom development in *U. bromivora*‐inoculated *B. distachyon* plants. These factors demonstrate that the practical use of the PhenoBox is not limited by its ‘cheap’ imaging hardware, emphasizing its broad applicability.

Open hardware, while not as established as open source software releases, has been gaining popularity in science in recent years, due to its potential to dramatically reduce the cost of lab equipment and machinery (Gibney, 2016). The release of the PhenoBox/PhenoPipe as open hardware and open source not only provides unlimited access to the scientific community, it also has the advantage that it allows modifications to the hardware and code to adapt it to specific requirements. For example, it is conceivable to modify the PhenoBox to fit other plant sizes or to integrate other camera systems, such as multispectral or hyperspectral cameras, or to use the system for long‐term observation of individual plants. While the current setup of the PhenoBox is realistically limited to the analysis of plants with significant vertical growth due to the side view setup of the camera, the same processing pipeline could be used in a modified PhenoBox using a top view camera to study, for example, rosette plants such as Arabidopsis. Such modifications can be collected to benefit the community, rather than for‐profit companies.

There is a growing need for standardization in the plant phenotyping field (Krajewski *et al*., 2015; Cwiek‐Kupczynska *et al*., 2016), to allow comparability of results from different labs and efficient digital data mining. While some other labs have developed lower cost phenotyping solutions (Chéné *et al*., 2012; Matos *et al*., 2014; Paulus *et al*., 2014; Tovar *et al*., 2017), our system is set apart by providing a user‐friendly and flexible framework covering the whole phenotyping process. Users can define plants in the database via the web interface, use the same interface to view and select images taken at different time points, and orchestrate feature extraction and data analysis steps. A stack system for the statistical evaluation of visual features allows for the combination of presupplied and custom‐developed evaluation modules written in the statistical programming language R and promotes exchange of evaluation modules between labs. Taken together, we hope that, by providing an easily accessible open source solution in the form of the PhenoBox/PhenoPipe system, we may contribute to the spread of affordable phenotyping capacity and to the establishment of interchangeable standards in the field.

### Applications of the PhenoBox/PhenoPipe system

We provide three application examples of the PhenoBox/PhenoPipe system for the study of plant stress responses by studying biotrophic fungal infections in *B. distachyon* and *Z. mays* and salinity stress response in *N. benthamiana*. These experiments demonstrate the broad applicability of the PhenoBox system to study biotic and abiotic stresses in monocot and dicot species of varied sizes and provide confirmation that established phenotypic responses are detected by our system. Beyond this, these experiments extend our insights into the stress situations we studied, particularly the phenotypic effects of biotrophic fungal plant infections on the whole plant.

We found alterations in quantitative growth traits and morphology of *B. distachyon* plants inoculated with the head smut *U. bromivora* that later developed qualitative infection symptoms in their spikelets. This allows for the accurate prediction of the infection outcome at the flowering stage from images taken in the early vegetative growth stage. Noninvasive detection of plant diseases, particularly by imaging techniques, is an emergent field due to its importance for agriculture (reviewed by e.g. Mahlein *et al*., 2012). Many methods concentrate on the detection and classification of characteristic disease symptoms such as leaf spots, blight and galls, options that are not applicable for the detection of endophytic infection or head smut infection during the vegetative growth stage. While other methods, such as the detection of volatile organic compounds (Goff & Klee, 2006; Vuorinen *et al*., 2007) or pulse‐amplitude‐modulation fluorometry (Rozpadek *et al*., 2015), may have the potential to identify differences between infected and uninfected plants, they are typically technically more challenging, expensive and lower‐throughput than the method presented here. While the PhenoBox methodology is not directly applicable to field situations, a methodology derived from our approach could be applicable in horticultural settings where plants can be individually evaluated. In a modified form, precision agriculture approaches could use high‐throughput field scanning platforms or unmanned airborne vehicles to scan for plants with abnormal, infection‐typical size and morphology traits. Moreover, the current setup is of high practical use for work in the *U. bromivora*–*B*. *distachyon* model system (Rabe *et al*., 2016), because it may drastically shorten the time to evaluate infection outcome, for example in fungal virulence factor or host R‐gene mutants, and allow for the targeted study of the infection stages before qualitative symptom establishment.

Infections of *B. distachyon* with *U. bromivora*, as well as of *Z. mays* with *U. maydis* led to early growth reduction of the infected plants compared to mock‐infected controls. While this is in agreement with observations of negative growth effects in *Digitaria sanguinalis* infected by the head smut *Ustilago syntherismae* (Gallart *et al*., 2009), different mechanisms could lead to this growth reduction. First, fungal pathogens and endophytes derive nutrients, mainly assimilated carbon and nitrogen, from their hosts (Faeth & Fagan, 2002; Fatima & Senthil‐Kumar, 2015), which could lead to nutrient limitation and thus reduced growth of the host (Cheplick, 2007). Also, plant defense triggered upon infection is a costly process, leading to a well‐described trade‐off between plant immunity and growth (Huot *et al*., 2014). Thus, energy invested into defense, in addition to the nutrient consumption by the fungus, could limit nutrient availability for growth. The observation that infected plants were delayed in senescence compared to control plants could support nutrient depletion as a cause of growth reduction, as this could be related to the so‐called ‘green island’/‘green bionissia’ phenomenon (Walters *et al*., 2008). This phenomenon describes the observation of delayed senescence in plant tissues infected by biotrophic pathogens, which, in turn, has been connected to sugar mobilization upon pathogen infection (Wingler & Roitsch, 2008). Alternatively, a signal triggered by the immune response could negatively regulate growth despite sufficient nutrient availability. Furthermore, morphological changes in the host can also be directly caused by fungal effectors, such as the changes in female inflorescence morphology triggered by the *U. maydis* effector SAD1 (Drechsler *et al*., 2016).

We also used our phenotyping system to compare phenotypic changes between maize plants infected with a fully capable *U. maydis* strain and plants infected with effector mutants that have previously been described to reduce infection symptoms. To quantify the phenotypic impact of infection by these different strains, we calculated multi‐dimensional distances between plant groups based on PCs derived from the visual features determined by our system. This approach, which is implemented in a PhenoPipe R module, is broadly applicable to the analysis of phenomic studies of all kinds of plant treatments, particularly when the relevance of individual traits is not known. It can provide a ‘quasi‐linear’ scale to compare treatment severity, even though this must be handled with care, as a linear comparison obviously lacks information on the differences in the types of phenotypic responses.

We found that during the early infection stage (4 dpi), infection with *U. maydis* effector mutants was indistinguishable from infection with the fully capable *U. maydis* strain (SG200). However, when infection symptoms were fully established (13 dpi), the mutant‐infected plants were far more similar to mock‐infected controls than SG200. At this stage they were not significantly different from controls based on the analyzed visual features, while the SG200‐infected plants were. This implies that a first effect on plant growth and morphology observed by 4 dpi is not critically dependent on the two effectors tested. At a later stage, the lack of these effectors inhibits the establishment of the pathogen and the host plant can recover phenotypically closer to mock‐infected control plants. These findings highlight the importance of looking at infection as a temporal process comprising multiple phases, rather than only an end outcome. Overall, visual phenotyping of infections with effector mutants at different infection stages can give insights about the nature, location and/or timeframe of the function of a given effector that cannot be revealed by symptom scoring alone.

Visual phenotyping of the response of *N. benthamiana* to salinity treatment revealed a significant reduction in plant size beginning 2 wk after the start of treatment, and a change in the distribution of pixel brightness values beginning 1 wk after the start of treatment, followed by changes in other color‐related traits, such as mean hue. Salinity affects plant growth by a variety of mechanisms, from the regulation of cell turgor and the cytoskeleton to epigenetic modifications (Yang & Guo, 2018). Changes in leaf coloration are commonly observed after severe or prolonged salinity exposure in different plant species (Begcy *et al*., 2011; Sakuraba *et al*., 2014; Jia *et al*., 2015) and are presumably due to changes in chloroplast organization, including Chl degradation (Sakuraba *et al*., 2014; Suo *et al*., 2017). These results confirm that the PhenoBox can detect phenotypic responses of plants to abiotic stress and is sensitive enough to detect changes in color‐related traits at stages where they are not detectable by the human eye.

In summary, we have demonstrated the use of the PhenoBox/PhenoPipe to study biotic and abiotic plant stress situations. Our results show that the PhenoBox can be used for the efficient detection of established phenotypic effects as well as to reveal new insights. We also present phenotyping approaches for the study of biotrophic fungal plant infections, emphasizing the added – and, in the case of head smuts, predictive – value of untargeted whole‐shoot phenotyping.

## Author contributions

A.D. and A.C‐E. designed the research. A.C‐E. performed plant phenotyping experiments, evaluated data and developed the classification methodology. J.J. and S.K. performed Lemnatec feature extraction. A.D., A.C‐E., U.G. and S.S. conceptualized the PhenoBox/PhenoPipe system. M.C. was head of the technical construction of the PhenoBox at the IMP workshop. S.S. developed the electronic setup and software for the PhenoBox, developed the PhenoPipe interface and the server‐side applications, and integrated IAP feature extraction and R data analysis modules into the PhenoPipe. A.C‐E. developed the R data analysis modules for the PhenoPipe. A.C‐E. and A.D. wrote the manuscript.

## Supporting information

Please note: Wiley Blackwell are not responsible for the content or functionality of any Supporting Information supplied by the authors. Any queries (other than missing material) should be directed to the *New Phytologist* Central Office.

**Fig. S1** Quantifying plant size and morphology traits from images.
**Fig. S2** Correlations between visual traits.
**Fig. S3** Comparison of visual features obtained by IAP between controls, successfully infected plants and plants that were pathogen‐inoculated but did not develop symptoms (‘failed spore formation’) imaged 21 d after vernalization.
**Fig. S4** Infection outcome prediction for *Ustilago bromivora* infection time points.

**Fig. S5** Pot adaptor model (.stp – can be opened by CAD programs).

**Fig. S6** Pot adaptor model (.pdf – to open this file, a 3D plug‐in for the PDF reader is needed).

**Table S1** Extraction of image features by IAP and Lemnatec software; correlation between features

**Table S2** Phenotypic differences between control, successfully infected and failed spore formation in *Ustilago bromivora* infection of *Brachypodium distachyon*

**Table S3** Phenotyping of maize plants infected with *Ustilago maydis* effector deletion strains

**Table S4** Phenotyping of NaCl stress effects in *Nicotiana benthamiana*

**Notes S1** Description of features extracted by the IAP software.

**Notes S2** Description of features extracted by the Lemnatec software.

**Notes S3** IAC file for Lemnatec analysis.

**Notes S4** PhenoBox documentation.

**Notes S5** PhenoBox system user guide.
