# Supplementary material for: The ‘PhenoBox’, a flexible, automated, open‐source plant phenotyping solution
Source: New Phytol. 2018 Apr 5;219(2):808–23. doi: 10.1111/nph.15129 (PMC6485332; doi:10.1111/nph.15129)
Supplement: Supplementary file 1 — Fig. S1 Quantifying plant size and morphology traits from images. Fig. S2 Correlations between visual traits. Fig. S3 Comparison of visual features obtained by IAP between controls, successfully infected plants and plants that were pathogen‐inoculated but did not develop symptoms (‘failed spore formation’) imaged 21 d after vernalization. Fig. S4 Infection outcome prediction for Ustilago bromivora infection time points. [file NPH-219-808-s001.pdf]

The “Phenobox”, a flexible, automated, open-source plant phenotyping solution

Angelika Czedik-Eysenberg, Sebastian Seitner, Ulrich Güldener, Stefanie Koemeda, Jakub Jez, Martin Colombini, Armin Djamei

Accepted 22.2.2018

Supplemental Figures

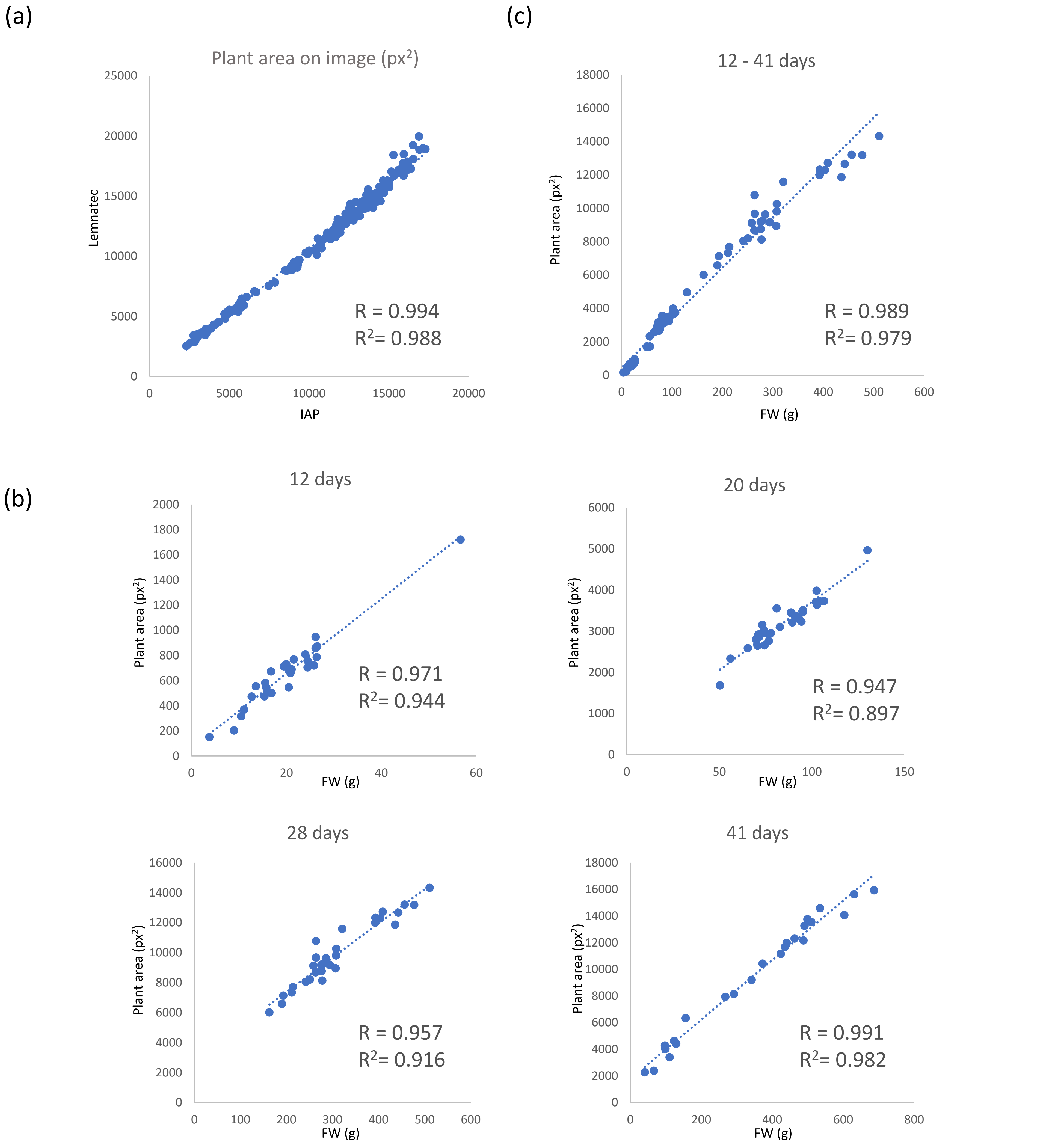

Fig S1: Quantifying plant size and morphology traits from images
a) Correlation between plant area on image in square pixels (px^2) extracted by Lemnatec and IAP software from images of 35 *Brachypodium distachyon* ABR4 plants taken 21 days after vernalization (dav).
b) Correlation between plant area extracted by IAP from images taken by the PhenoBox and shoot fresh weight (FW) determined from the same *Brachypodium* plants at time points from 12 days after planting to 41 days after planting. The 41 days time point also included Area and FW from partly dried plants, thus the larger spread of values. At each time point 26-30 plants were analyzed.
c) Correlation between plant area and FW when data from all time points displayed in b) are pooled.
R.. correlation coefficient, R^2.. coefficient of determination from Pearson correlation

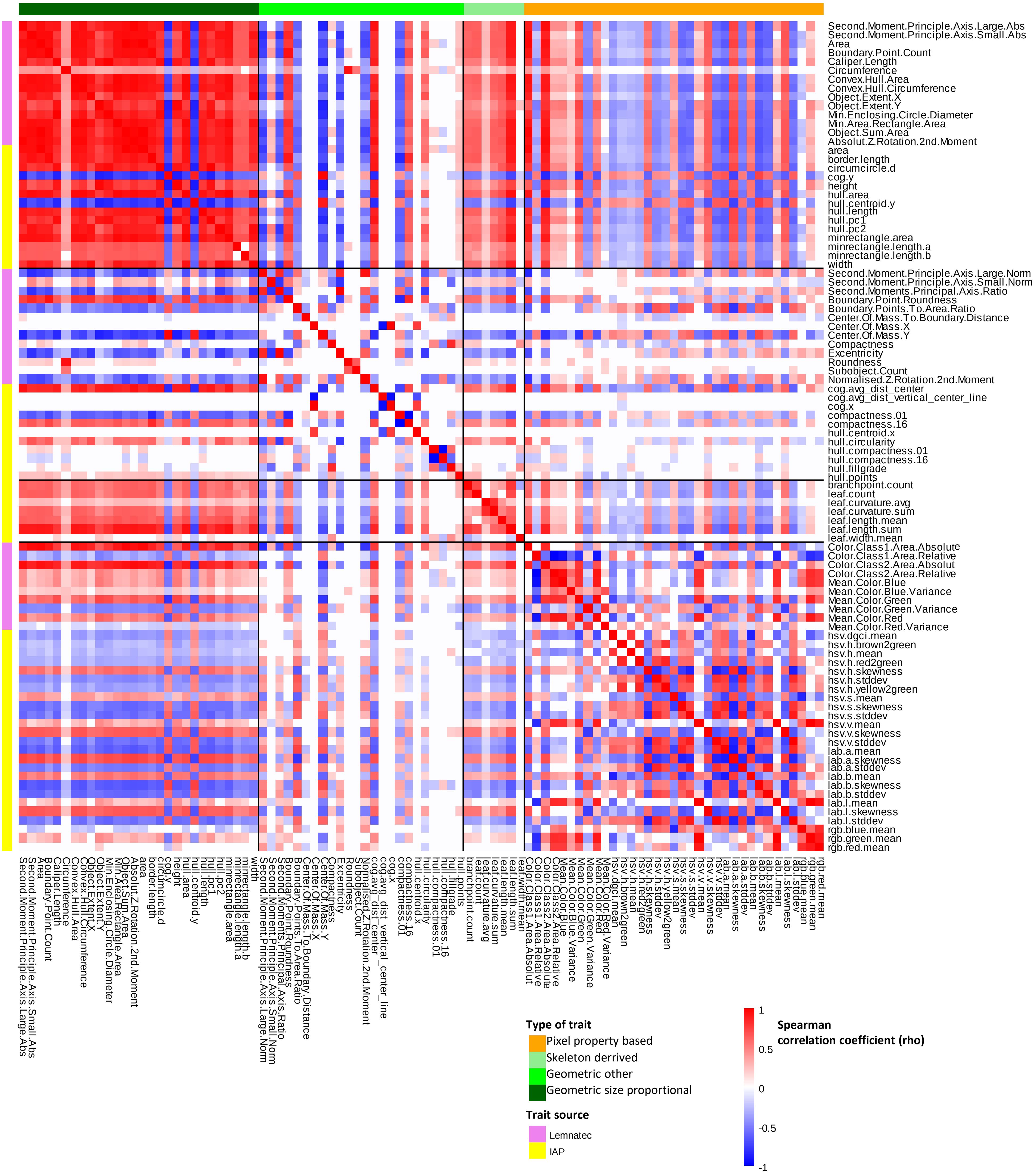

Fig S2: Correlations between visual traits

Spearman correlations between all visual traits extracted by Lemnatec (pink side bar) or IAP software (yellow side bar) from images of 35 *Brachypodium distachyon* ABR4 plants taken 21 days after end of vernalization treatment. Rows and columns are sorted by type of visual trait. Lists detailing all traits extracted by the Lemnatec software and IAP software are given in Notes S2 and S3. Correlation p-values were multiple testing corrected by the Benjamini-Hochberg algorithm and only correlations with corrected p-values <0.05 are shown. Red: positive correlations, blue: negative correlations. The darker the color, the stronger the correlation. See Table S1 for numeric rho and p-values.

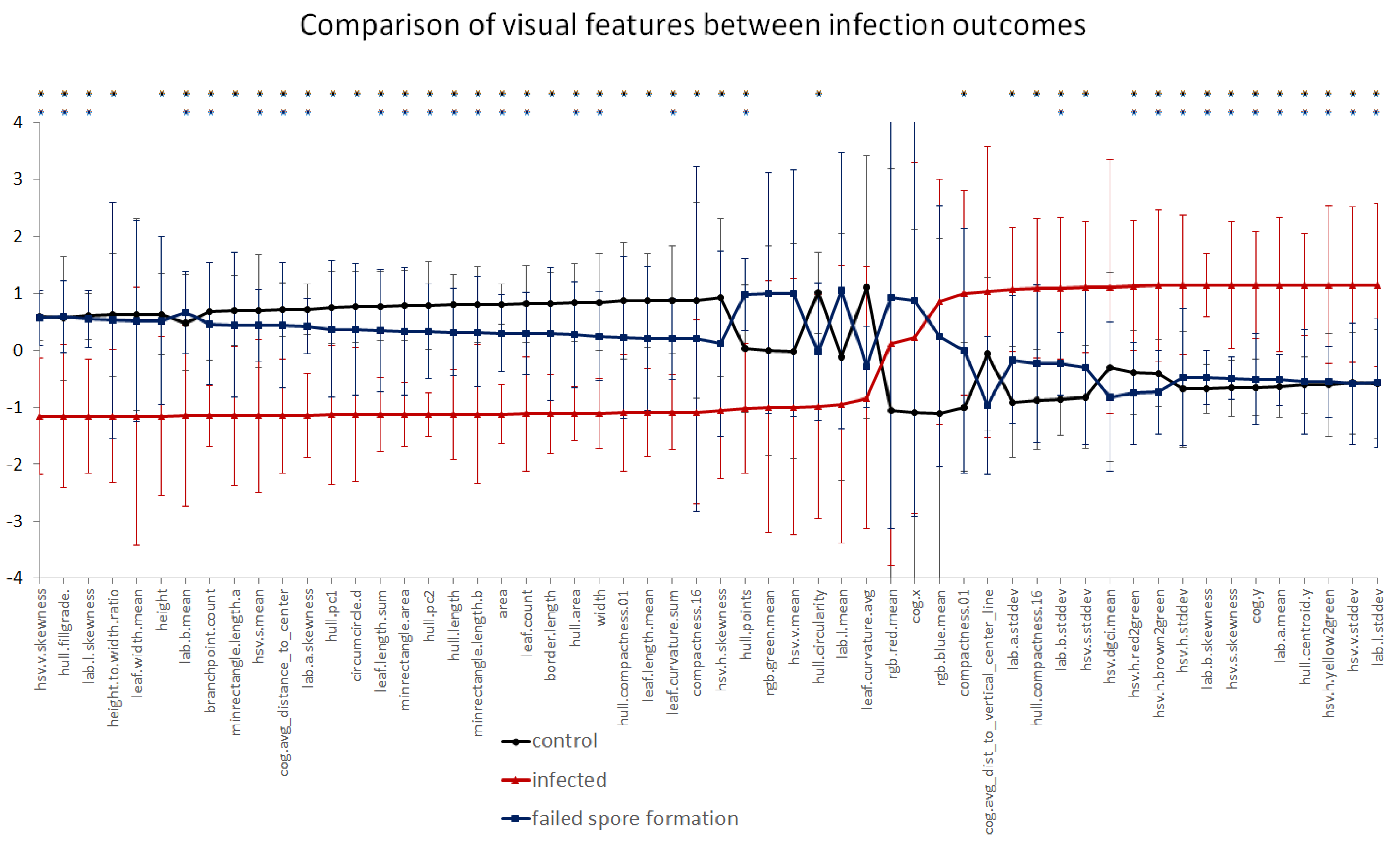

Figure S3: **Comparison of visual features obtained by IAP between controls, successfully infected plants and plants that were pathogen-inoculated but did not develop symptoms ("failed spore formation") imaged 21 dav**

Feature were z-score normalized. Means and standard deviation are displayed. Top row with black star symbol indicates a significant difference between infected and control plants with a corrected p-value <0.05, second row with blue star symbol indicates a significant difference between infected plants and failed spore formation. P-values were corrected for multiple testing by the Benjamini-Hochberg method (Hochberg and Benjamini 1990).

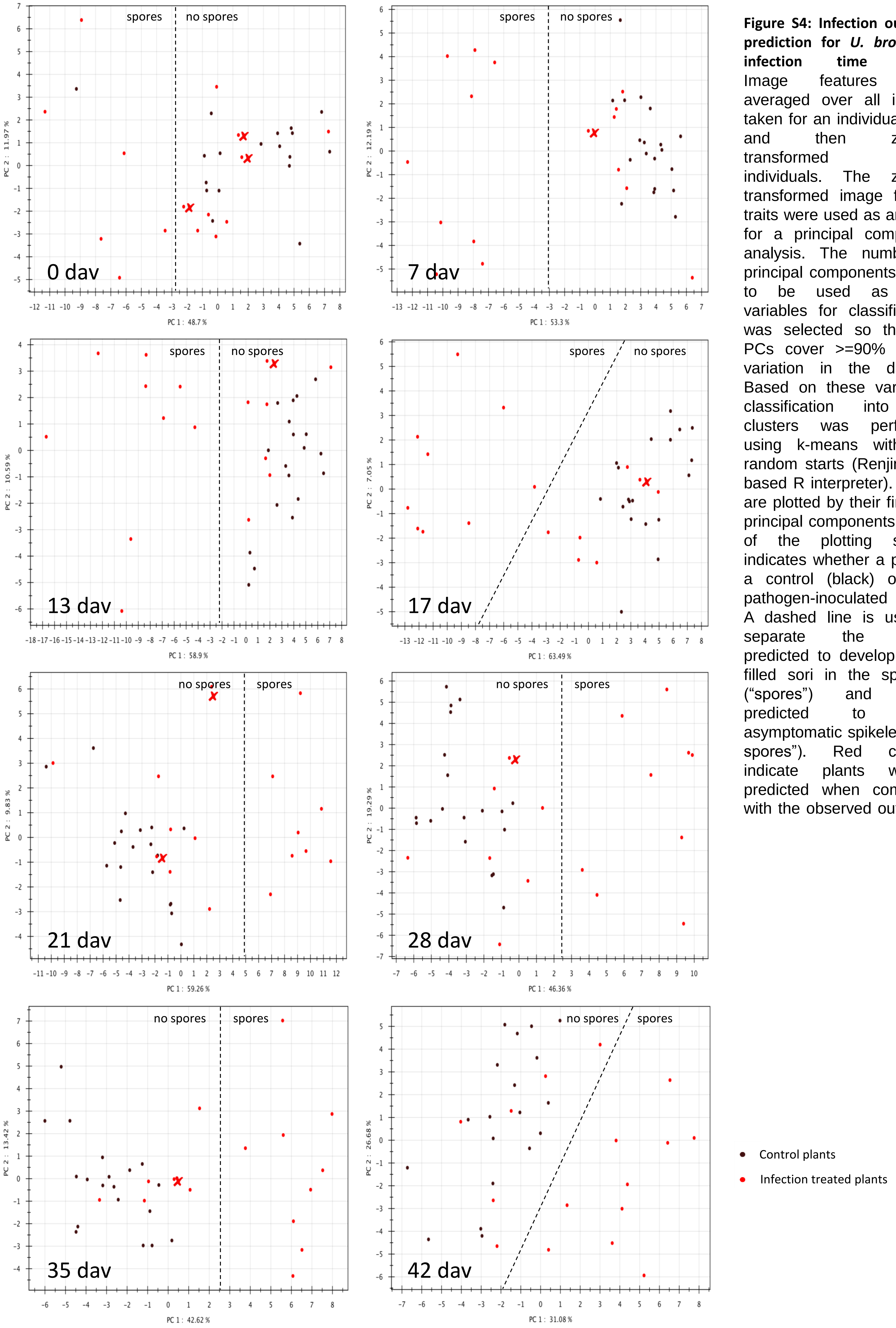

Figure S4: Infection outcome prediction for *U. bromivora* infection time points Image features were averaged over all images taken for an individual plant and then z-score transformed across individuals. The z-score transformed image feature traits were used as an input for a principal component analysis. The number of principal components (PCs) to be used as input variables for classification, was selected so that the PCs cover >=90% of the variation in the dataset. Based on these variables, classification into two clusters was performed using k-means with 100 random starts (Renjin Java based R interpreter). Plants are plotted by their first two principal components. Color of the plotting symbol indicates whether a plant is a control (black) or was pathogen-inoculated (red). A dashed line is used to separate the plants predicted to develop spore filled sori in the spikelets ("spores") and those predicted to have asymptomatic spikelets ("no spores"). Red crosses indicate plants wrongly predicted when compared with the observed outcome.

References:

Hochberg, Y., and Y. Benjamini. 1990. 'More powerful procedures for multiple significance testing', Stat Med, 9: 811-8.
