# Supplementary material for: The ‘PhenoBox’, a flexible, automated, open‐source plant phenotyping solution
Source: New Phytol. 2018 Apr 5;219(2):808–23. doi: 10.1111/nph.15129 (PMC6485332; doi:10.1111/nph.15129)
Supplement: Supplementary file 9 — Notes S2 Description of features extracted by the Lemnatec software. [file NPH-219-808-s009.pdf]

LemnaTec High-Content Screening

LemnaTec Analysis parameters

Matthias Eberius
LemnaTec GmbH
Pascalstr.59
52076 Aachen
Germany
matthias.eberius@lemnatec.com
www.lemnatec.com

Updated 22.1.2014

1 Introduction

The following text provides a short overview on analysis parameters of the LemnaTec image analysis systems. The measurement of these basic parameters is the result of the LemnaGrid analysis package. This package includes a large number of individual tools which are necessary to pre-process the images before they are ready to be quantified.

These tools are described separately.

It must be considered that the parameters described here are the basic image parameters. In subsequent steps these parameters can be combined and thus transformed to parameters of biological meaning. This biological meaning is strongly related to the experimental design as the same parameter may have different meanings depending on the analysis objects and the context in which they are imaged.

1.1 *Morphological parameters*

The following list is a short documentation of the basic morphology-related parameters which can be extracted from any image. These images may originate from visible light, near-infrared, fluorescence, and height scan or, e. g, heat images. Even on hyperspectral images these morphological parameters can be applied, e. g, on index based ration images leading to biologically relevant values and evaluations.

1.1.1 Object characterisation parameters

The following list provides the shape parameters for objects found on images characterizing objects with an area and extension

1. BoundaryPointCount: Number of all pixels of an object boundary
2. BoundaryPointRoundness: BoundaryPointRoundness = $(\text{BoundaryPointCount})^2$/size.
3. DiagonalBoundaryCount: Number of boundary pixels being connected diagonally to the previous one
4. HorizontalBoundaryCount: Number of boundary pixels being connected horizontally to the previous one
5. VerticalBoundaryCount: Number of boundary pixels being connected vertically to the previous one
6. RatioDiagonalBoundaryCount: RatioDiagonalBoundaryCount = DiagonalBoundaryCount/BoundaryPointCount
7. RatioHorizontalBoundaryCount: RatioHorizontalBoundaryCount = HorizontalBoundaryCount/BoundaryPointCount: RatioVerticalBoundaryCount
8. RatioVerticalBoundaryCount = VerticalBoundaryCount/BoundaryPointCount
9. CaliperLength: Maximum distance between two points on the object boundary
10. Excentricity: Excentricity = $\text{sqr}(\mu20 - \mu02) + 4 * \text{sqr}(\mu11)/\text{sqr}(\mu20 + \mu02)$
11. MinAreaRectLargeExtension: Longer side length of non-oriented bounding box
12. MinAreaRectSmallExtension: Shorter side length of non-oriented bounding box

13. MinAreaRectAngle: The angle measured describes the angle between 3 o'clock/east and the larger extension side of the rectangle.
14. ObjectExtentX: Length in x-direction of the minimum vertically oriented rectangle covering the object
15. ObjectExtentY: Length in y-direction of the minimum vertically oriented rectangle covering the object
16. MinEnclosingCircleDiameter: This value describes the radius of the minimum hole the object would be able to pass through
17. 2ndMoment20Abs: $\mu 20$ = S (pixelA * rAx^2), rAx is the distance of pixel A from the y-axis (through the centre of mass) in x-direction.
18. 2ndMoment02Abs: $\mu 20$ = S (pixelA * rAy^2), rAy is the distance of pixel A from the x-axis (through the centre of mass) in y-direction.
19. 2ndMoment11Abs: $\mu 11$ = S (pixelA * rAxrAy), rAy is the distance of pixel A from the x-axis (through the centre of mass) in y-direction; rAx is the distance of pixel A from the y-axis (through the centre of mass) in x-direction.
20. 2ndMoment20Norm: 2ndMoment20Norm = 2ndMoment20Abs/ObjectArea
21. 2ndMoment02Norm: 2ndMoment02Norm = 2ndMoment02Abs/ObjectArea
22. 2ndMoment11Norm: 2nd_mom_norm_mu11
23. 2ndMoment11Norm = 2ndMoment11Abs/ObjectArea
24. 2ndMomentPrincipleAxisLargeAbs: μl = S (pixelA * rAs^2), rAs is the distance of pixel A from the larger moment axis (through the centre of mass) in direction of the smaller moment axis with the additional condition $\mu 11 = 0$
25. 2ndMomentPrincipleAxisSmallAbs: μs = S (pixelA * rAl^2), rAl is the distance of pixel A from the smaller moment axis (through the centre of mass) in direction of the larger moment axis with the additional condition $\mu 11 = 0$
26. 2ndMomentPrincipleAxisLargeNorm: 2ndMomentPrincipleAxisLargeNorm = 2ndMomentPrincipleAxisLargeAbs/ObjectArea
27. 2ndMomentPrincipleAxisSmallNorm: 2ndMomentPrincipleAxisSmallNorm = 2ndMomentPrincipleAxisSmallAbs/ObjectArea
28. 2ndMomentPrincipleAxisAngleToLargeMoment: The angle between the larger 2nd moment principle axis and the x-axis is calculated as 2ndMomentPrincipleAxisAngleToLargeMoment = 0,5*arctan(2m11/(m20-m02)).
29. 2ndMomentsPrincipalAxisRatio: 2ndMomentsPrincipalAxisRatio = 2ndMomentPrincipleAxisLargeAbs/2ndMomentPrincipleAxisSmallAbs
30. ObjectArea: Number of object pixels
31. AbsolutZRotation2ndMoment: μz = S (pixelA * rA^2) rA Euclidian distance from the centre of mass
32. NormalisedZRotation2ndMoment :μz = (S (pixelA * rA^2))/ObjectArea
33. Compactness: Compactness = ObjectArea/ConvexHullArea
34. CentreOfMassX: Average x-value (x-position) of all object pixels (all pixel same size). **Formula:** CentreOfMassX = S pixelA*rAx/Object area. It is the x-value of the crossing point of the two principal axes.
35. CentreOfMassY: Average y-value (y-position) of all object pixels (all pixel same size). **Formula:** CentreOfMassX = S pixelA*rAy/Object area. It is the y-value of the crossing point of the two principal axes.
36. Circumference: Sum of the direction corrected length of the outer boundary of all subobjects
37. SubobjectCount: Count number of physically non-connected object units

38. CentreOfMassToBoundaryDistance: The shortest distance between the centre of mass and a point
39. ConvexHullArea: ConvexHullArea is the area of the smallest object without concave boundary parts
40. BoundaryPointsToAreaRatio: BoundaryPointsToAreaRatio = BoundaryPointCount /ObjectArea: 0 to 1 /no unit

1.1.2 Skeleton parameters

Skeleton analysis is always important when long and small objects are reduced to their centreline and these "bones" are morphologically characterised again. Following the concept of a skeleton, a skeleton consists of bones connected by joints.

Application examples: Skeletons are important in all cases where the very basic architecture and spatial arrangement could be a basis for phenotyping or just the quantification of length, orientation, number of connected parts of one organism (corn plant with leaves and stem) or a number of organisms, e. g. larvae).

1. BoneCount: Number of all the bones in the image
2. JoinCount: Number of all the joints in the image
3. Final name: SingleBoneCount: Number of bones without joints, thus not connected to any other bone
4. EndBoneCount: Number of bones with one joint, being at the end of the skeleton
5. InnerBoneCount: Number of bones with two joints being in the inner part of the skeleton
6. SumBoneLength-: Length of all bones lengthcorrected for diagonal orientation
7. SumSingleBoneLength: Length of all SingleBones length-corrected for diagonal orientation
8. SumEndBoneLength: Length of all EndBones length-corrected for diagonal orientation
9. SumInnerBoneLength: Length of all InnerBones length-corrected for diagonal orientation
10. SumBoneDirHorizontal: Number of all horizontally oriented pixels of all bones (not orientation corrected)
11. SumBoneDirVertical: Number of all vertically oriented pixels of all bones (not orientation corrected)
12. SumBoneDirDiagonal: Number of all diagonally oriented pixels of all bones (not orientation corrected)
13. RelativeBoneDirHorizontal: Ratio of all horizontally oriented pixels of all bones (not orientation corrected)
14. RelativeBoneDirVertical: Ratio of all vertically oriented pixels of all bones (not orientation corrected)
15. RelativeBoneDirDiagonal: Ratio of all diagonally oriented pixels of all bones (not orientation corrected)
16. SumEndBoneDirDown: Number of all vertically downwards oriented pixels of all EndBones (not orientation corrected). Only EndBones can define 5 directions as they have a start at a joint. Nevertheless, upside (45° upwards) does not distinguish between 45° left or right due to the rotational symmetry of most objects to be analysed.

17. SumEndBoneDirDownSide: Number of all 45° downwards oriented pixels of all EndBones (not orientation corrected)
18. SumEndBoneDirSide: Number of all sidewards oriented pixels of all EndBones (not orientation corrected)
19. SumEndBoneDirUpSide: Number of all 45° upwards oriented pixels of all EndBones (not orientation corrected)
20. SumEndBoneDirUp: Number of all vertically upwards oriented pixels of all EndBones (not orientation corrected).
21. RelativeEndBoneDirDown: Ratio of all vertically downwards oriented pixels of all EndBones
22. RelativeEndBoneDirDownSide: Ratio of all 45° downwards oriented pixels of all EndBones
23. RelativeEndBoneDirSide: Ratio of all sideward oriented pixels of all EndBones
24. RelativeEndBoneDirUpSide: Ratio of all 45° upwards oriented pixels of all EndBones
25. RelativeEndBoneDirUp: Ratio of all vertically upwards oriented pixels of all EndBones

1.2 *Colour based object parameters and transformation tools*

In addition to the shape and size of objects the colour of the objects is always an important feature to quantify plant phenotypes. The original more than 16 Mio colours of colour images and the grey shades of other camera images must be transformed in advance on the complete images or after object separation on the individual object.
What is described "colour" here may have different meanings for different camera systems as shown below. Please consider that more "meanings" can be derived if the original image colour is transformed by image analysis procedures like ratio calculation between images etc.:

Camera type	Original meaning of "colour"
Visible light camera	red, green, blue
NIR camera	Relative amount of water in tissue/substrate based on strong absorbance of NIR light by H2O molecules
IR Camera	Surface temperature
Blue Fluorescence camera	Excitation of certain fluorophores such as Chlorophyll, GFP, YFP, and phenolic compounds.
Chlorophyll fluorescence camera	Photosynthetic system status and performance calculated from time-dependent fluorescence kinetics (Kautsky effect)
Hyperspectral cameras	Reflective intensity of a wavelength band
Height scan	Height

1.2.1 Colour classification

Colour classification is used to separate all colours of an image into biologically relevant classes defining colour shades or e. g specific effects e. g. chlorosis.

Application examples: To assess the fitness (nutrition state, age, stress) of a plant over time or to differentiate between stress response due to different treatments plant the user defines e. g. 5 colour classes (dark, medium and light green, chlorotic, necrotic) and adds manually by selection from an image some representative anchor colours to each colour class represented by one false or signal colour. After the analysis all colours of the leaves will be assigned to one of the colour classes.

Technical description: In a first step a set of colour classes represented by a name and an anchor colour is defined by the user. The user assigns a set of representative colours to each class by clicking on respective areas on one or more original images. The colours of each single pixel is then assigned to the nearest (Euclidean distance in RGB space) representative colour in the RGB Cartesian space and labelled with the anchor colour linked to the colour class. The number of pixels is counted for each colour class.

1.2.2 Nearest neighbour foreground/background colour separation

All colours of an image are assigned to either the object colour class or the background colour class to separate them.

Application examples: Analysing green and yellow leaves on a soil background – containing a wide range of colour shades from dark brown to almost white, particle-like vermiculite – is best done based on colour. The same applies if a blue background is used for plants with a high variability of colours and colour shades from dark green to white (e. g. flowers).

Technical description: A number of representative colours are selected in a colour classification dialogue for both background and objects (foreground). Each single pixel is assigned to one or the other class by the nearest neighbour rationale in the RGB space.

1.2.3 HSI Colour Space Converter

Converts a given an RGB input image to one of the three components of the HSI colour space. HSI stands for Hue, Saturation, Intensity.

Application examples: Depending on the test sample, characteristic colours may be less important than e. g. saturation. This enables for example the normalisation of different green shades caused by different leaf thickness.

Technical description: Non-parametrical transformation of RGB to HIS is followed by a selection of one of the channels Hue, Saturation or Intensity for further use as a greyscale image.

1.2.4 Red/Green division

The Red/Green division was specifically developed to separate healthy green from damaged yellowish or brownish leave areas, and in many cases this is done automatically.

Application examples: For leave damage assessment, separating healthy green areas from damaged areas can be almost completely automated by using a low number of parameters to characterise the green areas and defining the border to damaged areas.

Technical description: The value of the red channel of each pixel is divided by the value of the green channel. The resulting value is rescaled and an offset is added.
Red /Green division is part of a larger algorithm projecting all Cartesian colour coordinates to the red/green level. A kind of green “hill” appears which can be easily separated from non-green areas as it is logarithmically transformed and the borderlines are characterised as lines through the white point. For more details please see also the patent of LemnaTec.

1.2.5 RGB calculator

This highly flexible device allows recalculation of any kind of value based on the 3 colour channel values and using the 4 basic arithmetic operations (*, –, +, :) to produce a greyscale image for further use.

Application examples: In cases where colour shades may differ massively and specific objects – e. g. yellowish lesions on leaves of very different green shades – are to be separated for better recognition, specific ratios between colour channels are used. The most suitable ratio strongly depends on the image characteristics.

Technical description: For the final greyscale value any mathematical calculation based only on the 4 basic arithmetic operations and the 3 colour channel values of the respective pixel is possible.

1.2.6 RGB to Grey Converter

To convert an RGB image to a greyscale image, each channel can be weighted with an individual amplification factor.

Application examples: In many cases just a few colour channels are important to identify a specific kind of object. The weighting mechanism can be used to extract those channels (or just one channel) that include the information and thus produce higher contrasts and

avoid interfering effects. This is often valid for the identification of lesions on leaves and especially helpful for stained samples or e. g. images from fluorescence experiments.

Technical description: Each colour channel is weighted with an individual factor before using the sum value as grey shade value. Value grey shade = $Wr*R+Wg*G+Wb*B$ where R, G, B are the values of the RGB channels and Wx is the weighting factor of the channel x with the additional condition being $Wr+Wg+Wb=1$.

1.2.7 Contrast enhancer

The Contrast enhancer is an important image processing tool if test objects have low absolute contrasts or the background varies greatly between images.

Application examples: The Contrast enhancer is an important image processing tool for test objects with low absolute contrasts or backgrounds changing from image to image (e. g. due to backlight imaging with different leaf thickness or growth or degradation stages).

Technical description: This device enhances the contrasts at the edges by calculating the value of the derivation (identification of the local gradient) within the image. The result/output is either this value or a multiplicative overlay of the values in the original image with the gradient.

Mask size describes the distance in pixels that is used to search for contrasts/gradients. The higher the value, the smaller the gradients that can be detected (as going over longer distances means having the same absolute contrast change as high gradients over short distances). Intensity is a parametric value to describe the extent of contrast change. The higher this value is, the smaller the contrasts that are recognized as potential borders of objects. The whole procedure is very sensitive and will detect even small contrast steps. To eliminate very small contrast steps and particularly undesired substructures within objects, the hooklet **Multiplicative Overlay** causes superposition of the greyscale image after the weighting of colours to the stressed gradient image. Thus gradients producing no significant change of the whole object contrast can be eliminated.

1.2.8 Device – Image Absolut values

Calculates the absolute positive value of each pixel with a given RGB or greyscale image. Some filters or image calculations may result in negative values. This device is useful to map these negative values into the positive value range.

Application examples: Negative values – resulting for example from the calculation of movement out of a set of images – need a mapping on the respective positive values

before they can be revisualised properly on the result image. In general, negative values can occur if two or more images are subtracted from each other or if filter masks include negative values).

Technical description: Calculates the value of each item assigned to a pixel.

1.2.9 Device – Image adder

Calculates the sum of pixel values (greyscale or RGB) for each pixel of an arbitrary number of input images, which can then be changed by clicking on the sum symbol.
Application examples
To detect movement on a series of images, single images are added as part of the whole algorithm. To enhance contrasts, original images and massively contrast-enhanced images can be added as well. As colours are generally not suitable to produce consistent results, greyscale images are added in most cases.

Technical description: Calculates pixel by pixel the sum of all input pixel values. By right clicking on the symbol in the centre of the device, additional functions can be activated in a context menu box. All input images must strictly be of the same type, either all greyscale or all RGB, and have the same size. All input boxes must be used in all cases. Missing input images will generate error messages and inhibit further analysis.
The output image is based on floating point values. To rescale the image into the normal 0–255 value range, a division must take place after the image adding, using the Image amplifier device. If this is not done, all values higher than 254 are set as being white by many subsequent devices.

1.2.10 Device – B/W Image amplifier

Multiplies each value of each pixel (greyscale) with a user-defined value. The amplifier device is used if all pixel values of a picture are to be made proportionally darker or brighter.

Application examples:
Amplification of images to make them brighter (factor > 1) makes sense e. g. for movement difference images, where the resulting raw value is too low (images to dark) to be visible. Using a constant value allows comparison of different images.
The use of factors smaller than 1 is particularly necessary if earlier image processing steps have produced floating point values higher than 256, the maximum value of images to be visualised. If such results are not reduced proportionally by the amplifier, later image processing devices will just set all values higher than 254 as white. This could be used to enhance dark values while ignoring bright details.

Technical description: An amplifier multiplies all grey shade values of each pixel of a picture by a constant factor higher than 1 to mark it brighter or lower than 1 to make it darker.

1.2.11 Device – Image compare

Compares two greyscale images pixel by pixel by setting the respective pixel white if the condition (first value larger, equal, smaller or smaller equal, larger equal than the values of the second one) is fulfilled. The condition can be selected by clicking on the symbol in the centre of the box.

Application examples: May be used to build regions of interests.

Technical description: Compares two greyscale images pixel by pixel by setting the respective pixel white if the condition (first value larger, equal, smaller or smaller equal, larger equal than the values of the second one) is fulfilled. Finally, this is a logical operator producing a binary image.

1.2.12 Device – Image inverter

The image inverter generates the classical negative image out of any kind of greyscale or RGB image.

Application examples: The image inverter can be used to invert a binary image that defines identified interferences (like conveyor belts as background objects) and exclude it from further image processing as part of the region of interest.

Technical description: Each pixel value (greyscale or each RGB channel separately) is mirrored at the value 128 (e. g. 136 is transformed to 120 or 255 (white) to 0 (black)).

1.2.13 Device – Image mean

Calculates the mean of pixel values (greyscale or RGB) for each pixel and pixel value of an arbitrary number of input images, which can then be changed by clicking on the mean symbol in the centre of the device.

Application examples: This device is often used to reduce camera noise, by calculating the mean over a time series of images (without movement), taken with the same camera settings. To detect movement on a series of images, mean values of different single images are calculated as part of the whole algorithm.

Technical description: Calculates the mean of pixel values (greyscale or RGB) for each pixel and pixel value of an arbitrary number of input images separately.

1.2.14 Device – Image negate

All values of each pixel are multiplied by –1, making all positive values negative and vice versa.

Application examples: Used as part of complex imaging tasks.

Technical description: All values of each pixel are multiplied by –1, making all positive values negative and vice versa.

1.2.15 Device – Minimum-maximum operation

This logical operation selects out of two images always the brighter (max. value) or the darker (min. value) value.

Application examples: Use in special move algorithms.

Technical description: This logical operation selects out of two images always the brighter (max. value) or the darker (min. value) value.

1.2.16 Device – RGB image amplifier

Multiplies each value of each pixel (RGB) with a user-defined value for each channel separately. The amplifier device is used if all colour values of a picture are to be made proportionally darker or brighter.

Application examples: Amplification of images to make them brighter (factor > 1) makes sense e. g. for movement difference images where the resulting raw value is too low (images to dark) to be visible. Using a constant value allows comparison of different images.
The use of factors smaller than 1 is particularly necessary if earlier image processing steps have produced floating point values higher than 256, the maximum value of images to be visualised. If such results are not reduced proportionally by the amplifier, later image processing devices will just set all values higher than 254 as white. This could be used to enhance dark values while ignoring bright details.

Technical description: Multiplies each value of each pixel (RGB) with a user-defined value for each channel separately. The amplifier device is used if all colour values of a picture are to be made proportionally darker or brighter. Only positive factors can be used.

1.3 *Other plant specific parameters derived from non-image sensors*

The most common other sensor and parameter set are the data from watering and weighing. Each time a plant is weighted the weight of the plant is measured and thus the loss of water compared to the last measurement or a pre-set target-value is written into the database. As water is added again the weight value before and after watering provides the amount of added water.

1.4 *General non-plant specific parameters derived from other sensors*

Depending on the configuration of the system additional values e. g. related to climate conditions in the greenhouse may be stored in the database.

1.5 *Derived parameters*

Based on the parameters mentioned above there are two options to further process the data. This might be done either on the image basis connecting two or more images by a specific algorithm based on the resulting dataset on pure numerical level. While all image based calculation are done by LemnaGrid as part of advanced image analysis, data based calculation are performed by the LemnaMiner as part of advanced spreadsheet calculation in the LemnaBase database.

All measurements (taking images, weighing, watering etc.) are related to a timestamp which allows deriving rate values.

As the list of derived parameter is very long and widely configurable by the user of the system the following paragraphs just show some aspects.

1.5.1 Hyperspectral Imaging

1.5.1.1 Indices overview

Hyperspectral indices are derived from two or more frequency band based images of one hyperspectral dataset taken by one camera at one time point. If such a set of images is available the LemnaGrid analysis package allows calculation the indices as shown below. The following table will provide a short overview on different indices which care related to plant phenotyping in literature. A more extensive paper on details is available separately including explanations on their application. The special focus is on identifying indices which are especially suitable for plant phenotyping under controlled conditions to extract a maximum amount of information.

The table provides an overview on the formulas of hyperspectral indices which are mentioned below.

Index name	Lowest nm	Highest nm	Formula
Simple ratio 800,550	550	850	SR800,550 = R800/ R550
Modified Chlorophyll absorption in Reflectance index (MCARI)	550	700	MCARI=[(R700- R670) – 0.2 * (R700-R550)]*((R700/R670)
Modified Chlorophyll Absorption in Reflectance Index (MCARI1)	550	790	MCARI1=1.2*[2.5(R790- R670) – 1.3 * (R790-R550)]
Soil adjusted vegetation indices (XSAVI)	670	800	SAVI = (1+L)(R800-R670)/ (R800+R670+L)
Optimised Soil Adjusted Vegetation Index (OSAVI)	670	790	OSAVI = (1+0.16)* (R790-R670)/(R790+0.16)
Gitelson and Merzlyak Indiex1	550	750	GMI1 = R750/R550
Gitelson and Merzlyak Indicex2	700	750	GMI2 = 750/700
Red Edge Normalized Difference Vegetation Index NDVI705	705	750	NDVI705 = (R750 – R705) / (R750+R705)
Modified Red Edge Simple Ratio Index	445	750	mSR705 = (R750 – R445) / (R705+R445)
Modified Red Edge Normalized Difference Vegetation Index	445	750	mNDVI705 = (R750 – R705) / (R750+R705-2*R445)
Greenness Index	554	677	G = R554 / R677
Vogelmann Indicex1	720	740	Vog1= R740 / R720
Vogelmann Index2	715	747	Vog2= (R734-R747) / (R715-R726)
Vogelmann Index3	715	747	Vog3= (R734-R747) / (R715-R720)
Transformed CAR Index (TCARI)	550	700	TSARI =3* [(R700- R670) – 0.2 * (R700-R550)]*((R700/R670)
Simple Ratio Pigment Index (SRPI)	430	680	SRPI = R430/R680
Normalised Phaeophytinization Index NPQI	415	435	NPQI = (R415- R435)/R415-R435)
Carotenoid Reflectance Index 1	510	550	CRI1= 1/R510 – 1/R550
Carotenoid Reflectance Index 2	510	700	CRI1= 1/R510 – 1/R700
Anthocyanin Reflectance Index 1	550	700	ARI1=1/R550 – 1/R700
Anthocyanin Reflectance Index 2	550	800	ARI2=R800 *(1/R550 – 1/R700)
Plant Senescence Reflectance Index	500	750	PSRI = (R680 –R500) / R750
Photochemical Reflectance Index (PRI)	531	570	PRI = (R531 –R570)/(R531+R570)
Nitrogen related index NRI1510	660	1510	NRI 1510 = (R1510-R660)/(R1510+R660)
Nitrogen related index NRI850	660	850	NRI850= (R850-R660)/(R850+R660)
Normalized Difference Nitrogen Index	1510	1680	NDNI=[log (1/R1510) –log(1/R1680)]/ =[log (1/R1510) +log(1/R1680)]
Normalized Pigment Chlorophyll Index (NPCI)	430	680	NPCI = (R680 – R430)/ (R680+R430)

Carter Index1	420	695	Crt1 = R695/R420
Carter Index2	695	760	Ctr2 = R695/R760
Lichtenthaler Index1	680	790	Lic1 = (R790- R680)/ (R790+R680)
Lichtenthaler Index2	440	690	Lic2 = R440/R690
Structure Insensitive Pigment Index (SIPI)	445	800	SIPI = (R790 – R450) / (R790+R650)
NVDI Turf Colorimeter	660	850	
Water Band Index	900	970	WI = R900 / R970
Water index (Thiel, Rath , Ruckelshausen)	1050	1450	WI = R1450 / R1050
Normalized Difference Water Index	860	1240	NDWI = (R860 – R1240) / (R860 + R1240)
Moisture Stress Index	820	1600	MSI = R1600 / R820
Normalized Difference Infrared Index	819	1649	NDII = (R819 – R 1649) / (R819 + R 1649)
Desease-Water Stress Index 1	800	1660	(DSWI-1) = R800/R1660
Desease-Water Stress Index 2	550	1660	(DSWI-2) = R1660/R550
Desease-Water Stress Index 3	680	1660	(DSWI-3) = R1660/R680
Desease-Water Stress Index 4	550	680	(DSWI-4) = R550/R680
Desease-Water Stress Index 5	550	1660	(DSWI-5) = (R800+R550)/(R1660+R680)
Leaf structure index R1110/R810	610	1110	LSI =R1110/R810
Normalized Difference Lignin Index	1680	1754	NDLI = (log(1/R1754)-log(1/R1680)) / (log(1/R1754)+log(1/R1680))
Cellulose Absorption Index	2000	2200	*CAI = 0.5*(R2000 +R2200) – R2100*

1.5.1.2 Selection of hyperspectral cameras

Due to the physical limits of sensor systems the wavelength range of hyperspectral cameras are limited. Plants are moving (at least the leaves) and cameras for different wavelength ranges often have different resolutions both on the wavelength and the spatial range.For this reason it is extremely helpful if all wavelength values needed for the calculation of hyperspectral indices are imaged with the same camera. The following table shows a large compilation of hyperspectral indices and which camera ranges are the best to image as many hyperspectral indices as possible.

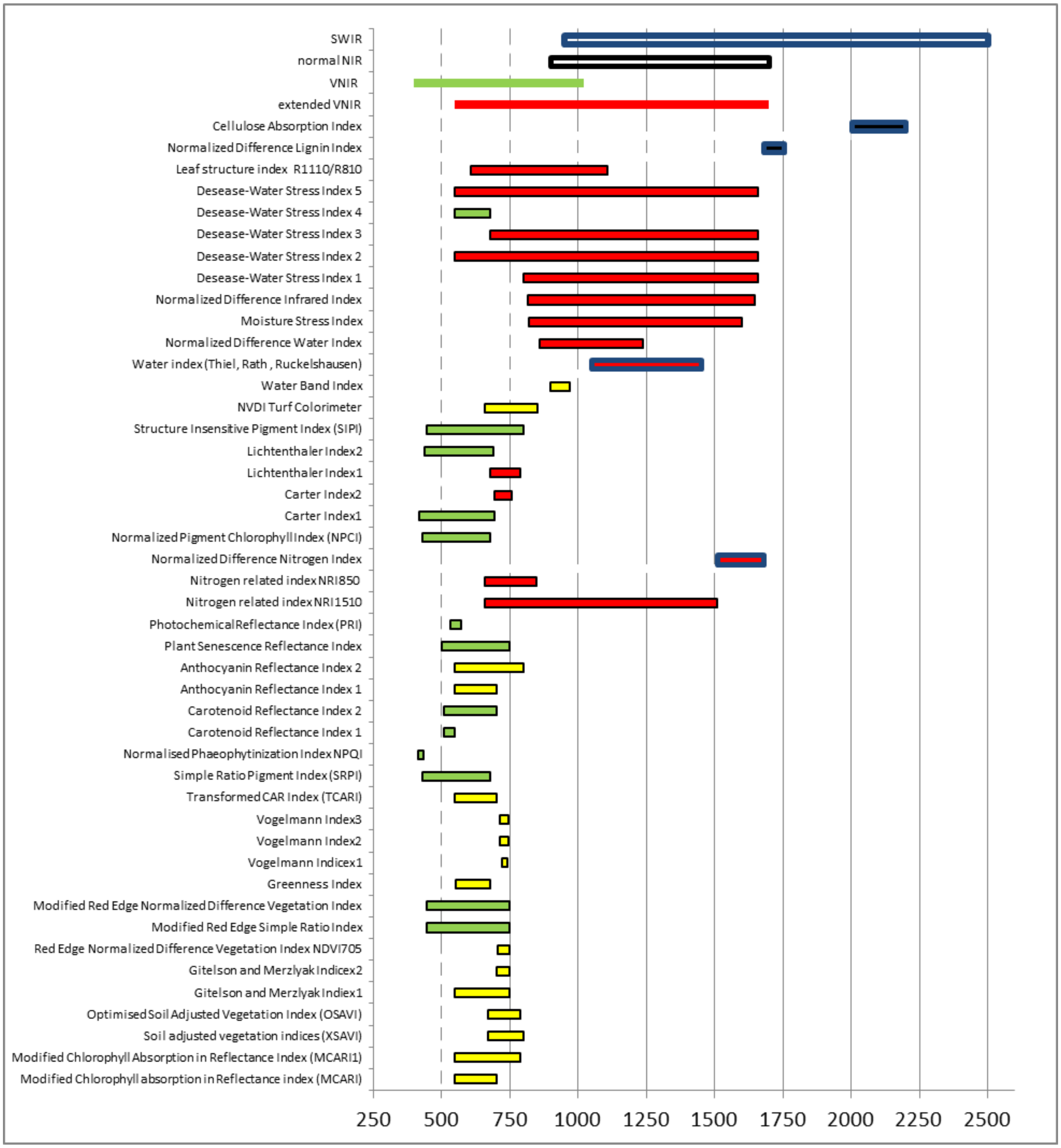

Figure: Graphs showing which indices can be imaged by which cameras. The colored bars show the range of required wavelength. Green and yellow bars/indices can be imaged by VNIR cameras (380-1000 nm), yellow and red bars/indices extended VNIR (550-1700 nm). NIR cameras do not cover the important range lower than 900 nm. For SWIR cameras the number of existing indices is still very restricted to cellulose and lignin related indices.

1.5.1.3 Example for hyperspectral index calculation

The following data are a first subset of hyperspectral data which were acquired for 3 different turf samples (SuF, TR, PR) which were treated differently (KO = control; HAL = impact of salt; SD = lowered light intensity). A hyperspectral camera for a range of 550 to 1700 nm was used.

The images below show calculation of 18 different indices based on top hyperspectral images of around 10 by 15 cm sizes of the turf samples. The index images show color encoded intensity values.

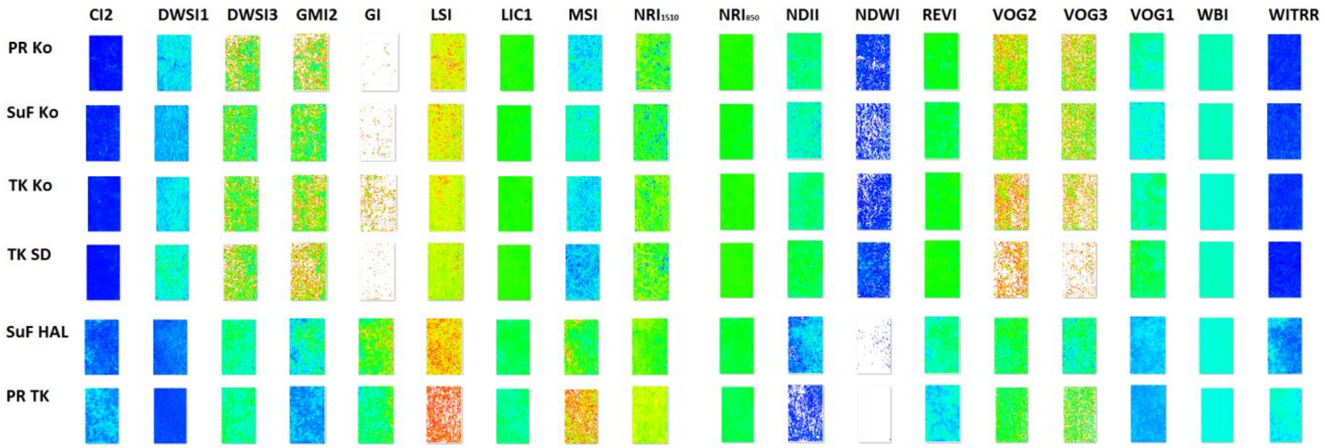

Figure: shows how different turf types and different treatments result in different intensity patterns.

While the control samples are relatively similar, the SuF sample is the most differ. It relates to a comparatively broad leafed turf sample with high salt and drought resistance. The SD treatment is still relatively similar to its TK control while the salt treatment altered the samples SuF Hals significantly relative to TK Ko. The most different sample is the drought stress sample PR TK.

These kinds of images can be used again for any kind of image analysis as documented above identifying e. g. specific morphological effects.

1.5.2 Rates (change of a parameter in time)

1.5.2.1 Growth rate:

Repeated measurements of the digital biomass based on leaf area from different sides can be used to compute the measured growth rate and to develop a growth model. Growth rate and change of growth rate especially if implemented in growth models is a very sensitive parameter reflection plant stress or integrated assimilation rates under the genetic and environmental conditions to which the plant is actually exposed.

1.5.2.2 Evaporation rates

Based on the measured water loss values a daily evaporation rate can be calculated.

LemnaTec GmbH
Matthias Eberius
Pascalstr. 59
52074 Aachen
Germany
+49 (0)2408 9383-102
matthias.eberius@lemnatec.com
