# Supplementary material for: The ‘PhenoBox’, a flexible, automated, open‐source plant phenotyping solution
Source: New Phytol. 2018 Apr 5;219(2):808–23. doi: 10.1111/nph.15129 (PMC6485332; doi:10.1111/nph.15129)
Supplement: Supplementary file 10 — Notes S3 IAC file for Lemnatec analysis. [file NPH-219-808-s010.pdf]

The Integrated Analysis Platform

User Documentation for IAP V1.1

Christian Klukas

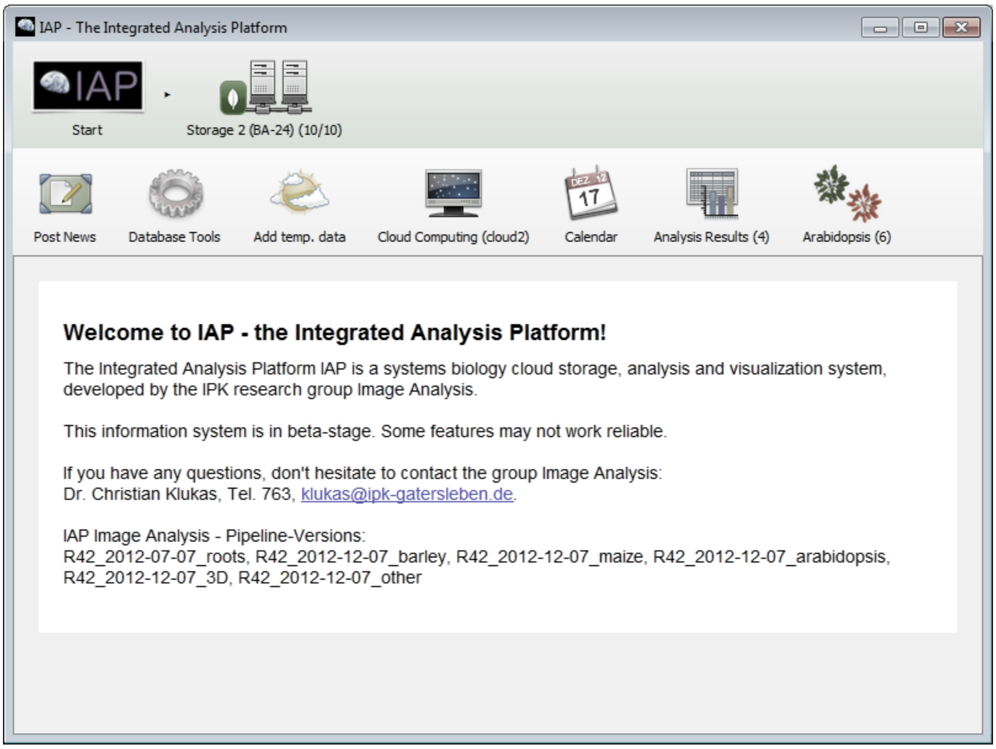

V5, November 2013

Content overview

Development history and objective ... 5
Installation ... 5
Software and hardware requirements ... 5
Downloading and installing the software ... 5
Removal of the software ... 5
User Interface ... 6
The three sections of the GUI ... 6
Multi-Document-Interface ... 6
Command bookmarks ... 6
Configuration ... 6
Direct LemnaTec-DB image-file transfer ... 7
MongoDB data storage ... 7
Installation and configuration of MongoDB ... 8
Connecting IAP to the MongoDB database instance ... 8
File-based data storage (local or remote) ... 8
MongoDB data storage combined with File-based data storage ... 9
Experiment progress monitoring ... 9
Adding webcam images to the monitoring mails ... 10
Adding desktop screenshot images to the monitoring mails ... 10
Observing the control PC desktop live from a remote PC ... 11
Automated copy-service ... 11
Data import ... 11
Access to exported LemnaTec-DB datasets ... 11
Importing climate data ... 12
Importing data from other data domains ... 13
Image analysis ... 14
Image analysis customization ... 14
Starting the analysis ... 18
Result interpretation ... 19
Data export ... 19
Command reference & Background Information ... 19
Understanding the command line output ... 19

Multithreading 20
Appendix 20
List of tables 20
List of images 20
Complete list of calculated traits 22
Meta data columns 22
Data columns calculated only for side camera images 23
Data columns calculated only for top camera images 25
Data columns calculated for top and side camera images 25
Data columns calculated by combining data from top and side camera images 35
Keywords 37
Revision History 42
Legal and Trademarks 42

This is the first public release of the software and the documentation. If you feel a part of it is incomplete, incorrect or unclear we would be happy if you contact the group image analysis by mail or phone.

Development history and objective

The Integrated Analysis Platform (IAP) has been development by the IPK research group "image analysis" since May 2010. The overall software design and architecture has been envisioned and implemented by the head of the group and author of this documentation. The image analysis pipeline development has been supported by J.M. Pape as part of his work for his Bachelor thesis and by Dr. A. Entzian, who also developed the PDF-report-function R and LaTex code.

The software system IAP is first and foremost focused on supporting the analysis of high-throughput imaging datasets, mainly sourced from automated greenhouse and imaging control systems, e.g. from the LemnaTec® [1] GmbH, Wuerselen, Germany. IAP is flexible enough to process image data from other imaging systems. The system is also able to process data from other data domains, as it incorporates the complete functionality of the VANTED system. IAP aims in bridging phenotype data with data from other domains and will be extended in the future to more easily link these data domains.

Installation

Software and hardware requirements

IAP has been developed using the Java™ framework. This means, that execution of the application is possible on all operating systems, which provide a compatible execution environment (Java Runtime Environment, JRE of version 1.7 or higher, sometimes called Java 7 or higher). Preferred are 64-bit versions of Java, as they support the utilization of more than 1600 MB of RAM. Enter "java -version" into the command line of a terminal window[2], in order to check if Java is correctly installed and has the proper version. IAP has been developed and tested under Windows® 7, Mac® OS X® 10.8 and the Linux® distribution Scientific Linux 6.3.

Hardware requirements are a mouse, at least 4 GB RAM and a display resolution of at least 1024x768 pixels.

Downloading and installing the software

Download IAP from the following website: ***http://iap.ipk-gatersleben.de***. After downloading the file IAP.zip, extract it and move the "iap" folder to the desired location. Use the operating system - dependent start-scripts within the folder to launch IAP.

Removal of the software

IAP creates a working-directory for its settings. Some commands also write their output into this directory or into sub-directories. To easily locate and open this operating system-dependent folder click ***Settings*** and then ***Show Config-File*** from the IAP start screen. To confirm that you are seeing the

[1] "LemnaTec" is sometimes abbreviated in the software and in this documentation as "LT" or "Lt".
[2] To open a command terminal window under Windows, open the run-dialog by pressing the Windows-key and then in addition the R-key. Enter "cmd" into the input-field and click "OK". On a Mac, open the Terminal application by using spotlight to search for "terminal". Under Linux, a terminal should be available from the application starter (menu).

settings folder you should check that it contains the file ***iap.ini***. Then close the IAP program and delete the contents of the settings folder. Then delete the “iap” program folder (see previous section) from your system.

User Interface

The three sections of the GUI

The user interface of IAP is divided into three sections. The first button list contains the ***Start*** button and the command history. The start-screen shows next to the start button the defined bookmarks. The second button list contains the commands, related to the currently selected command (last item in first button row). The area below the two button bars shows command results. It remains unchanged if a selected command produces no output for this window section.

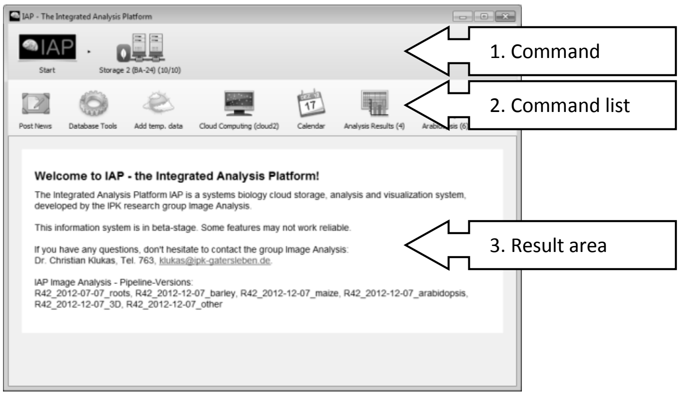

1 The three user interface sections of IAP

Multi-Document-Interface

In order to work more flexibly with the system, e.g. to load and compare more than one dataset, it is possible to open additional command windows, which provide the same functionality as the main window. To open a new command window, right-click on the first or second button-bar and choose ***New Window***. If you close the main command window (identifiable by its title and the “earth”-window-icon), the program will stop execution and all windows will immediately be closed. If you close a newly opened command window, only the corresponding window is closed.

Command bookmarks

You may quickly return to often used data sources or datasets by creating command bookmarks which are shown in the first button list on the start screen.

To create such bookmark, first navigate to the desired command. Then move the mouse over the small ***right-pointing arrow*** (▶) on the left hand side of the currently selected command. The arrow will change into a small ***writing pen*** (🖊). Clicking onto this pen icon activates the start-screen and adds the new bookmark right next to the ***Start*** button.

To remove a bookmark, move the mouse cursor to the left of the particular bookmark button and over the ***small flower icon*** (❁). The icon will change into a ***crossed rectangle*** (☒). Click onto this rectangle to remove the related bookmark.

Configuration

To simplify usage of the software enable only commands relevant to your work or your work environment. Click ***Settings*** on the start screen to customize IAP.

Direct LemnaTec-DB image-file transfer

If you are used to system administration and plan to regularly copy and transfer datasets including its metadata with a "one-click-operation" from a LT database server, it is recommended to follow the configuration procedures. If the configuration makes problems (e..g. if meta-data cannot be found) or if the server installation is not compatible or for other reasons a direct connection is not preferred, skip the following section and follow the descriptions in the next chapter in the section "Data Import" on how to use the LT supplied database export tool and then on how to import such dataset and related metadata into IAP (section "Access to exported LemnaTec-DB datasets").

This application supports the direct import of image datasets and metadata from the LemnaTec database. To enable database access, click and enable ***Settings* ‣ *Lt Db* ‣ *Show Icon***. The settings sub-group ***Image File Transfer*** contains properties[3], listed in table 1, page 7. Modify these settings as needed for your server settings and network environment

1 *Settings ‣ Lt Db ‣ Image File Transfer Settings*

Setting	Description
Use Scp Instead of Ftp	Images (if configured in this way) are transferred from the LT database using FTP (default) or using the encrypted SCP file transfer protocol.
Ftp Host	Specifies the host name, used for FTP file transfer.
Ftp Directory Prefix	Specifies the directory prefix (used to change the directory during the FTP session), to access the root folder of the image storage location.
Ftp User, Ftp Password	Contains user name and password (symmetric encrypted) for FTP access.
Scp Host	Specifies the host name, used for SCP file transfer.
Scp Local Storage Folder	Specifies the directory prefix (used to change the directory during the SCP session), to access the root folder of the image storage location.
Scp User, Scp Password	Contains user name and password (symmetric encrypted) for SCP access.
Use MongoDb Data If Available	If enabled, images which have been previously copied to a MongoDB database instance from the LT database server will be retrieved from the MongoDB database instead of from the LT database server.
Use Local File Access If Available.	If enabled and available, direct file access will be used to access image data. Local file structures are available when executing the program on the LT database server, mounting the same image-file storage-location on the local computer, or by copying the directory structure to the local PC.
Local Copy or Mount Point	If the previous setting is active, the specified directory is used to access LT image data.

MongoDB data storage

"MongoDB (from "humongous") is a scalable, high-performance, open source NoSQL database." [Source: www.mongodb.org] It can be used within IAP for storing, retrieving and managing image datasets, analysis results, and data from other data domains. Image datasets which are stored in a

[3] If this sub-group is not shown, click the *Start* button to show the start screen, click the *LemnaTec* button, wait for completion or error, click *Start* again, then go back to the *Settings* section, and click *Lt Db*.

MongoDB database can be processed using several compute servers or PCs in parallel (grid computing), which increases processing speed considerably.

If this database is already installed, you can skip the following section and head to the IAP MongoDB® configuration description at the end of this section.

Installation and configuration of MongoDB

Go to ***http://www.mongodb.org***, click ***DOWNLOADS*** and download the latest (64-bit version) of MongoDB for your operating system. Unzip the downloaded file archive and move the executables to the desired target folder. Use a startup-script similar to the following code to start the database instance:

2 *MongoDB example start script (for Linux)*

```
#!/bin/bash
cd /home/klukas/mongodb-linux-x86_64-2.2.2/bin
ulimit -d 20000
./mongod --auth --dbpath /media/16TB/MongoDB
```

To create a database with access only to authenticated users, start the database process using the startup-script and then start the command ***mongo*** from the 'bin' directory. Type ***use storage1***, to create the database named "storage1", then type ***db.addUser("iap", "pass")***, to create the user account named "iap", with password "pass". You can authenticate later by first typing again ***use storage1***, then ***db.auth("iap", "pass")***. Refer to the MongoDB documentation for further administrative tasks.

Connecting IAP to the MongoDB database instance

Start IAP and click ***Settings* ‣ *Grid-storage* ‣ *N***, and enter the value "1", to specify one MongoDB storage location. Now click ***Start*** again, then ***Settings* ‣ *Grid-storage-1***. Click ***Enabled***, specify the storage location title (e.g. "Storage 1"), the database name ("storage1", see previous paragraph), the login name ("iap") and the password ("pass"). The "Host" setting should be the fully qualified host name or IP of the database server or computer. As you click ***Start*** again, the new storage location ***Storage 1*** will is listed and available as a copy-command target.

File-based data storage (local or remote)

Experiment data can be stored in the MongoDB database, but may also be stored locally in a file folder. To define a corresponding storage location, click ***Settings* ‣ *Vfs***[4] ‣ ***Enable***. Click ***Start*** and then ***Settings* ‣ *Vfs-1***. Click ***Enabled***, specify a storage location identifier (Url-Prefix, e.g. "desktop", "drive_c", …), enter the descriptive title ("Description", e.g. "Local Storage"), and the actual storage location ("Directory"). Vfs-Type should be "LOCAL", host, username and password can be left empty. As you click ***Start*** again, the new storage location ***Local Storage*** will be listed and also be available as a copy-command target. If you specify a remotely accessible resource, e.g. a FTP or SSH server (SFTP), you need to specify also user name and password, which will be stored using symmetric encryption in the IAP settings file.

[4] VFS stands for „Virtual File System“.

MongoDB data storage combined with File-based data storage

The current file storage architecture of MongoDB “GridFS” stores binary files in chunks as normal database objects. The disadvantage of this file handling is that the memory cache of the database is quickly saturated by such binary objects. Additionally, the MongoDB instance (and the database server) becomes more sluggish and more difficult to administer, if Terrabytes of data is stored in the database. Conventional databases avoid this bottleneck and problems, by providing mechanisms to store binary objects in the file system, storing only a link in the database. Currently MongoDB does not contain such feature. With IAP it can be simulated, by using a file-based data storage location for storing binary files. A VFS file storage location can be assigned to a MongoDB database within the IAP client application. File I/O is then redirected at first to the corresponding VFS storage location. Only if a file can’t be found in the VFS, the software automatically reverts to loading the data from the database instance. This kind of setup is recommended for storing very large amounts of experimental data in a MongoDB, with the connected advantages, e.g. the possibility to analyze datasets using multiple compute PCs/servers, and data de-duplication (any file is only stored once, as it is identified by its content hash value). You can switch to the VFS based file storage also at a later time point, when the MongoDB database files have grown too large.

Please refer to the two previous sections for setting up a MongoDB database storage location and a file-based storage location. If binary files are stored in a VFS location and not only within the MongoDB database, all IAP client installations need to be configured in this way, binary (image) data files are otherwise not accessible, in case only the MongoDB database access can be established. The VFS storage location needs to be accessible by all clients, it should therefore be a central file server, accessible as a locally mounted network drive, or by FTP, SFTP, Samba or other supported networking file exchange protocols.

To connect a VFS file storage location to a MongoDB database, go to ***Settings* ‣ *Vfs* ‣ *Vfs-X_1***, make sure, that the item is ***Enabled***, click ***Store Mongo-Db Files***. Enter the name of the MongoDB database (this value needs to be the same as ***Settings* ‣ *Grid-storage-X_2* ‣ *Database-Name***). If this VFS storage location should only be used for storing the MongoDB files, the setting ***Use Only For Mongo-Db Storage*** should be enabled. The storage location will then not be available as a target for copying Experiments and will not be listed as one of the Home-actions for experiment data browsing.

To move files from the MongoDB database to a VFS location, click from the start screen ***[Mongo Storage Location]* ‣ *Database Tools* ‣ *External File Storage* ‣ *Move data to [VFS name]* ‣ *Move [XX GB or all files] to VFS***. The action command “External File Storage” will only be shown if the VFS entry has been configured with the correct database name and is enabled. MongoDB will not automatically free-up disk space on the database server, ass soon as data has been removed from the database. Instead, as with MongoDB V2.2, you need to issue the database-repair command from the MongoDB shell, to free-up the disk-space (for details please refer to the MongoDB documentation on this topic[5]).

Experiment progress monitoring

Timely reactions to technical problems are essential for the proper execution of a phenotyping-experiment. IAP supports this by the so called IAP watch service, an automated procedure to send an E-Mail to a list of interested users, once new data has been captured (image data, watering or weight

[5] http://docs.mongodb.org/manual/reference/command/repairDatabase/#repairDatabase

data) by the LT system or once data capture has ended. If the start- and end-times are known, constant every day and specified in the watch-service configuration, such E-Mails are only send if data capture is (or is not) observed at times differing in comparison to the expected start and stop times.

To setup the LT-experiment-progress-monitoring, start IAP from the command line with the parameter “watch”, e.g. by issuing the following command:

3 *Startup-command for the IAP watch service*

```
java –Xmx256m -jar iap.jar watch
```

If this command is issued from within Windows, the watch-service configuration file (experiments.txt) will be shown automatically within the Windows file manager. In the same directory the file “mail-server.txt” contains the required settings for correctly sending E-Mails from the application. The settings are stored in the “watch” directory with the settings folder, which can be located within IAP by clicking ***Settings*** and then ***Show Config-File***. On Linux systems[6], the settings are stored in “~/.iap/watch”. The monitoring command adds the latest size-reduced snapshot images to each e-mail.

Adding webcam images to the monitoring mails

If configured, a current snapshot of defined web-cams can be added to each e-mail. Click ***Settings*** ‣ ***Watch-services*** ‣ ***IP cameras*** to specify the number ***N*** of the defined cameras. For each defined camera three settings can be specified (click ***Start*** ‣ ***System Status*** if not all of these settings are shown, after the ***N*** parameter has been increased): ***Webcam X enabled***, ***URL-X***, ***Content Type-X***. Access to each webcam can thus be enabled or disabled, the URL for obtaining a snapshot of the camera can be specified and the content type (e.g. “image/jpg” or “image/png”) can be specified. If the webcam is protected by access rights as defined in the HTTP protocol, you need to add the user name and password to the webcam URL by using the following syntax:

user:*pass*@http://lemnacam.ipk-gatersleben.de/jpg/image.jpg?timestamp=[time]

In this case also the current time (in ms) is added to the URL as specified by the replacement indicator "[time]”. After the first mails have been sent, the mapping of database name(s) to webcam(s) can be configured, by starting IAP graphically and clicking ***Settings*** ‣ ***Watch-services*** ‣ ***DB2WebCam***. For each monitored database up to two webcams can be selected from the list of defined webcams. In this way this function supports multiple greenhouses and multiple webcams. Mails may thus include only the relevant webcam images.

Adding desktop screenshot images to the monitoring mails

If the monitoring service is not executed in a command-line-only-environment (non-GUI or headless environment), the monitoring component automatically includes a screenshot of the PC desktop view (if started under Windows with available GUI). In this way also error dialog windows and the current status of the imaging system, as shown on the control PC, is included in each mail.

[6] If you are using the RedHat Linux operating system or a compatible Linux distribution, the watch-service can be configured as Linux service, by following the instructions, detailed in http://iap.ipk-gatersleben.de/watch/help.txt.

Observing the control PC desktop live from a remote PC

If the option ***Settings* ‣ *Watch-services* ‣ *Screenshot* ‣ *Publish Desktop*** is enabled in the graphical IAP GUI, the current view of the control PC desktop can be monitored at any time from a remote PC. A screenshot of the desktop will be created every few seconds and the corresponding database field in the MongoDB database will be updated (no history is saved, only the current desktop). On the remote PC the life view can be observed by clicking ***System Status* ‣ *[Host Name]***. The host-name buttons are only available, if a current screenshot is stored and availabe in the database. The publish-function can also be quickly enabled or disabled by clicking ***System Status* ‣ *Screenshot Storage [enabled/disabled]***.

Automated copy-service

The automated copy service can be enabled to automatically copy datasets from the LemnaTec database to a MongoDB storage location. At first it should be checked that the direct loading of experiment datasets from the LT database works. After that make sure that at least one MongoDB storage location has been defined and is working. Click ***Settings* ‣ *Watch-services* ‣ *Automatic Copy* ‣ *Enabled*** to enable the service. The property ***Starttime-H*** specifies the daily starting time, it should be set to the very early morning or late evening (default: 1 am), a time at which normally all imaging and watering runs have been completed. The copy process applies information on defined outliers and other properties (e.g. analysis settings) of previously stored datasets to the newly copied dataset. The service activities are saved in a log file, which can be displayed using the command ***System Status* ‣ *Watch-service History***. If the watch-service has been configured as described, it can also be temporarily disabled using the ***System Status* ‣ *Watch-service*** command, and also be re-enabled using this command. If the service is re-enabled using this command, the service gets active once at the very moment, in addition to the scheduled execution time.

Data import

Data can easily be transferred to local folder storage or to the MongoDB database, by opening the source data location. Click ***Imaging System***, then select the desired LT database name and click onto the experiment button command which corresponds to the desired measurement label. If the direct access has not been configured or is not working, export and load the datasets as described in the following section. Once a dataset is loaded, click ***Copy*** and then choose the desired copy-target.

Access to exported LemnaTec-DB datasets

IAP supports the direct import of image datasets and metadata from the LemnaTec database. To export an imaging dataset, start the program ***Lemna DBImportExport*** (from the Windows desktop). Then click ***Database-Login***, enter your user name and password and select the desired LT database. Then click the button ***Snapshot-Export***. Now choose the first option ***Export Dump.*** Then click ***Select Snapshots*** and choose sort by “measurement label” from the icon bar on the left. Now select the desired measurement label and click the right-pointing arrow button, repeat this step if you would like to export several experiments. Then click OK, choose the output directory, click the radio button option “don’t combine tiled images” and then ***Start*** to export the desired dataset(s).

This export includes image data, watering and weight data, but not the metadata (e.g. genotypes, treatments). Metadata is usually imported into the LT system, using a CSV-file/template. Such template could look similar to this layout, but may contain less or more columns:

4 *Example metadata table*

Barcode	OAC	Experiment	Species	Genotype	Variety	Pot
1214CK001	12Tray_Small_Plant_Top	1214CK	Arabidopsis thaliana	IL	N987654	12-tray
1214CK002	12Tray_Small_Plant_Top	1214CK	Arabidopsis thaliana	Col	N456789	12-tray

If a certain plant-treatment regime has been applied, this information should be stored in a column with the title "Treatment". Place the metadata CSV or XLSX file(s) into the main level of the exported directory hierarchy. On the start screen of IAP click the command button ***Load LT File Export***[7], select the folder which contains the exported data and the metadata, and click ***Select Input Folder***. After the first loading, you should go back to the settings and modify, if needed, the column assignment to the IAP metadata fields. Click ***Settings*** ‣ ***Metadata Import*** ‣ ***Columns*** and check and/or change the field assignment. Then go back and load the data with the corrected metadata. For each measurement-label an experimental dataset will be created, which can be copied to a MongoDB database or local file-based data storage folder, and subsequently analysed.

Importing climate data

Currently, two German greenhouse control software export formats (tabular text files) are directly supported by this function. Detailed format descriptions and example files will soon be placed on the IAP website. Export format number one stores the average temperature during day and during night as well as the start and end times for these two temperature regimes in separate columns. Export format two stores the average environment temperature for every hour during the day. It is planned to change this function in a timely manner as soon as we get feedback from users, who would like to process their temperature datasets. It would be best if you could send such example data files by mail to klukas@ipk-gatersleben.de, so that we can quickly extend the software to make it flexible in this regard and to support additional templates out of the box.

IAP supports the loading and processing of greenhouse climate data, most importantly of temperature data. Other data such as humidity information could be stored, but is not processed by build-in calculations. If temperature data has been assigned to a dataset, the numeric data export function transforms all time values from "days after sawing" (DAS) or "day of experiment" (DAY), into so called "growing degree days" (GDD) values. Using the GDD time unit datasets can more easily be compared, as plant growth is strongly dependent on environment temperatures.

To assign a temperature dataset to an experiment, first load the temperature dataset using the command ***Add temp. data***, click ***Add to Clipboard***, then load the analysis result dataset and click again ***Add to Clipboard***. Then click ***Start***, and then ***Merge Clipboard***. If you now click ***View/Export Data*** and export a numeric data file (***Create Spreadsheet (XLSX)***) or create an automated PDF report, the data file will show GDD time values and units instead of DAY or DAS. The time values can only be adapted accordingly, if the greenhouse temperature time values cover the time period of the imaging runs.

The baseline temperature (the temperature where no plant growth is assumed to happen), can be customized at ***Settings*** ‣ ***Growing-Degree-Days***. These options are only shown once temperature data has been assigned to an experiment and the export function has been executed as described at least once.

[7] If this command button is not shown, click *Settings* ‣ *Lemnatec-db* ‣ *Show-Loaded-Export-Icon*.

Importing data from other data domains

IAP may also process (store, retrieve, transfer, display) data from other data domains, for example metabolomics, proteomics and genomics. This functionality is available, as IAP is internally using ***VANTED***, as one of its base programming libraries (***http://vanted.ipk-gatersleben.de***). To differentiate the functionalities from an end-user perspective[8], VANTED is accessed within IAP from the start screen using the ***IAP-Data-Navigator*** command button. By default this functionality is not made visible, but it can be shown by clicking ***Settings ‣ VANTED ‣ Show-Icon***. Once the IAP-Data-Navigator is shown, experiment data can be loaded in the ***side panel Experiments*** in form of tabular data (CSV or XLSX), or from specially formatted templates[9]. Once the dataset has been loaded, it can be transferred into the MongoDB database or any other defined storage location using the action command button labelled ***Copy to IAP Storage***. The following image shows how such data can be loaded and where this command button is displayed:

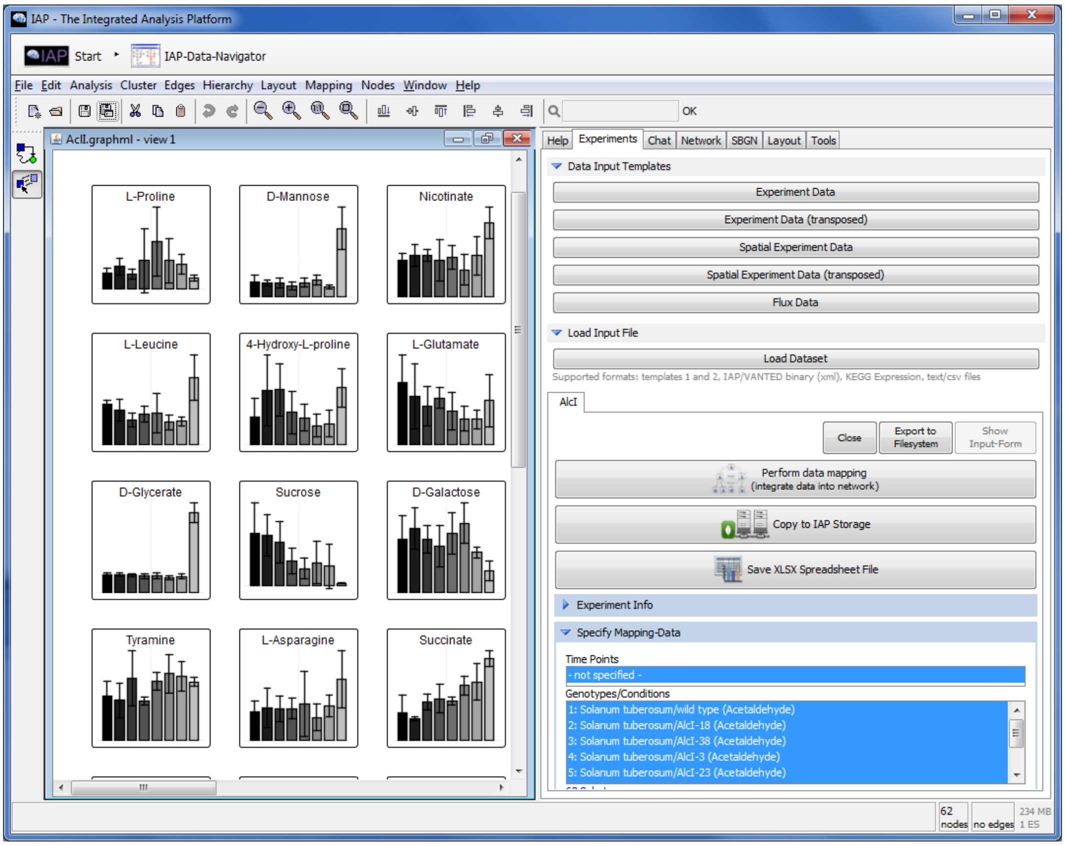

2 Using the IAP-Data-Navigator function to load and process data from other data domains.

Datasets (e.g. image analysis results), loaded from MongoDB or other storage locations can be shown and processed within the IAP-Data-Navigator user interface by clicking the command button ***Show in IAP-Data-Navigator***[10]. The experiment structure is slightly modified by this command. Sample fine times (images times are stored within IAP to the exact hour, minute and second) are normalized for this further processing, and reduced to whole day time values and units. The reason for this is that VANTED has been designed for data display and processing in more coarse time units.

---

[8] IAP includes extended functionality and data structures supporting the processing of high-throughput image datasets and may therefore not be 100% compatible with VANTED.

[9] Example datasets and detailed format descriptions are available at the following website: http://vanted.ipk-gatersleben.de/index.php?file=doc102.html.

[10] This command is not shown if the IAP-Data-Navigator icon display is not enabled.

This embedment of this functionality did not only speed-up and eased the development of IAP, it is also valuable, when phenotypic data should be related with data from other data domains. In addition, functionality (e.g. Add-ons) developed for VANTED can be more easily added and re-used for IAP (and vice versa).

Image analysis

IAP utilizes so called image analysis ***blocks***. A series of sequentially executed blocks forms an ***image analysis pipeline***. IAP initially provides at least one image analysis pipeline (Maize), but further specially tuned pipelines for Barley, *Arabidopsis* and other species are in preparation and included as preview code. The provided pipelines are called ***image analysis templates***. For analyzing an experiment such template needs to be assigned to the particular experiment. The pipeline is copied (duplicated) and stored within a storage field in the experimental data structure. The next section describes how to assign a template to an experiment and how to fine tune and change the settings for analysis.

Image analysis customization

First, navigate to the desired experiment. Data just loaded by the import function or from LT cannot be processed - only experiments which have been copied to a MongoDB database storage location or to a file-based storage location can be analyzed.

Once a dataset has been loaded, click ***Analysis* ‣ *Select Analysis Template***. Then choose the best fitting template (***Use [Template Name]***). The template will be copied and all changes to the assigned analysis pipeline will automatically be stored within the experiment structure, as soon as you change a setting. Other instances of IAP or additionally opened windows will pick-up these changes, normally in an instance. Only if you revert some settings to their default values, by deleting these settings with the command ***Defaults (delayed)***, you sometimes need to navigate back to the first item in the command history, and then back to the desired location, to fully refresh the active command buttons. As a template has been assigned, experiment specific changes and adjustments can be performed. To do so, navigate to the experiment and choose ***View/Export Data***. The experiment tree structure should list substance names such as “vis.top”, “vis.side”, “fluo.top”, “fluo.side”, “nir.top”, “nir.side”, “ir.top”, “ir.side”. The first part indicates the camera type (visible light/fluorescence/near-infrared/infrared), the second part the camera position (top/side). At this moment only these camera types and camera positions are supported[11]. You should check and optimize the analysis pipeline for top and side camera positions, if both are available. Drill down the tree structure to a particular image (e.g. some visible light image from top, from the last days of the experiment). On the right hand side of the window the small scale icons of the particular images will be shown in form of a button. By right-clicking onto a button you can display the particular image (***Show Image***), the so called Reference Image, or a so called Annotation Image. These types of images may not be initially available. After an analysis, the main image will be the analysis result image, the reference image will be the previous image. And the previous reference image will be available as a annotation image. The list of images behaves in this situation similar to a history stack. During the image analysis pipeline processing the so called “Snapshots” are processed, this means, that in every analysis up to four images (from the different camera systems), which belong to a single plant in a point of time, are

[11] If your imaging system contains other kinds of camera systems or positions, they need to be mapped to the listed types.

processed in parallel. So every image should be correctly connected to the other images of a snapshot. You can load and display all related images by right-clicking onto a button and choosing ***Show Complete Snapshot Set ‣ Main Image***. If related images are not displayed correctly, the import of the images was performed incorrectly (e.g. wrong and differing snapshot times or different replicate IDs[12]).

In the image-button context menu, the assigned template can be executed by two menu commands the title corresponds to the assigned template name (e.g. ***[Barley Analysis] (Image+Reference)***). This command should be used on the imported dataset. If you have opened the analysis result data set, the analysis pipeline steps and results can be checked with the second menu item (e.g. ***[Barley Analysis] (Reference+Old Reference)***). If you would like to check the results of a template, you can use the commands in the sub-menu ***Analysis Templates***.

After choosing an analysis pipeline from the image-button context menu, the related analysis pipeline is executed with the selected snapshot image set. When the analysis is finished, a new window will be shown:

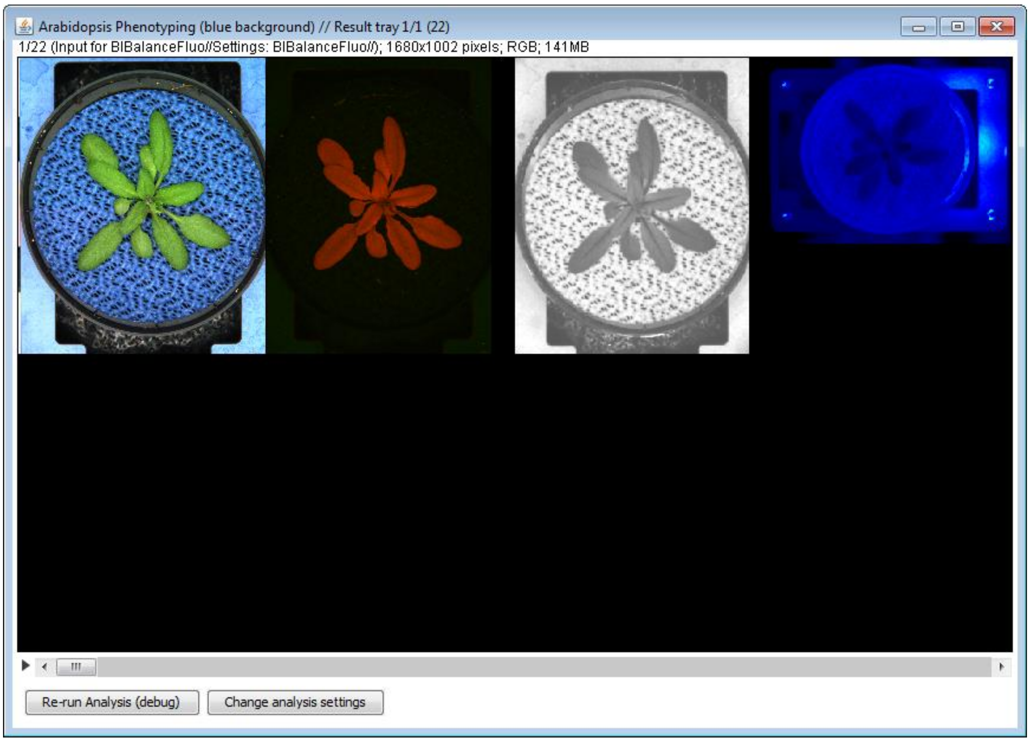

3 Image analysis pipeline results (displayed is the first step within the pipeline) for a particular snapshot. The images from the different camera types are displayed in the following order: visible light, fluorescence, near-infrared and infrared. If a snapshot contains a subset of these, the corresponding part stays empty. The lower part is used to display reference images in the beginning and intermediate results during the pipeline processing.

Within this window you may scroll (using the scroll bar at the bottom or using the scroll wheel of your mouse) through the individual pipeline steps and investigate the results of the analysis blocks. If you identify a problem, e.g. in the IR-image, the rotation is not correct; the pipeline setting for a

[12] A replicate ID is a unique number, which corresponds to a particular plant. IAP creates these numbers on the bases of provided text-IDs, e.g. carrier IDs or plant IDs. This number and the provided plant IDs are stored and processed within the system for identification purposes.

related analysis block needs to be changed. Click the command button ***Change analysis settings***[13] to open an additional window, which provides access to the pipeline configuration settings:

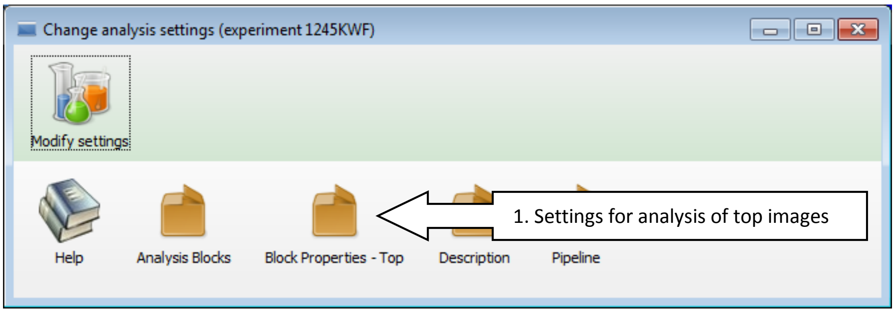

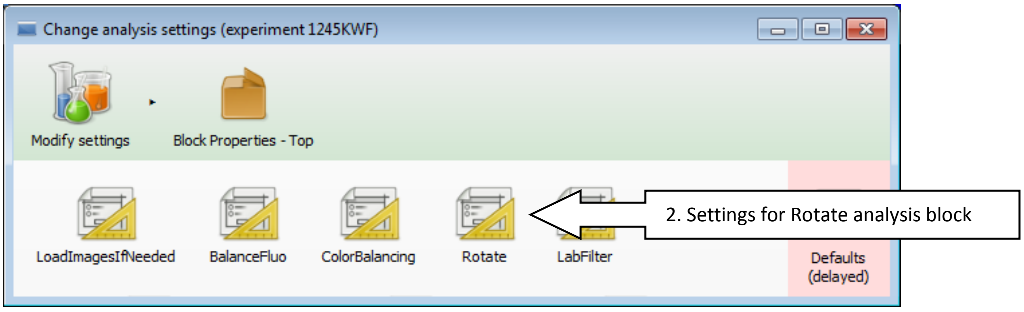

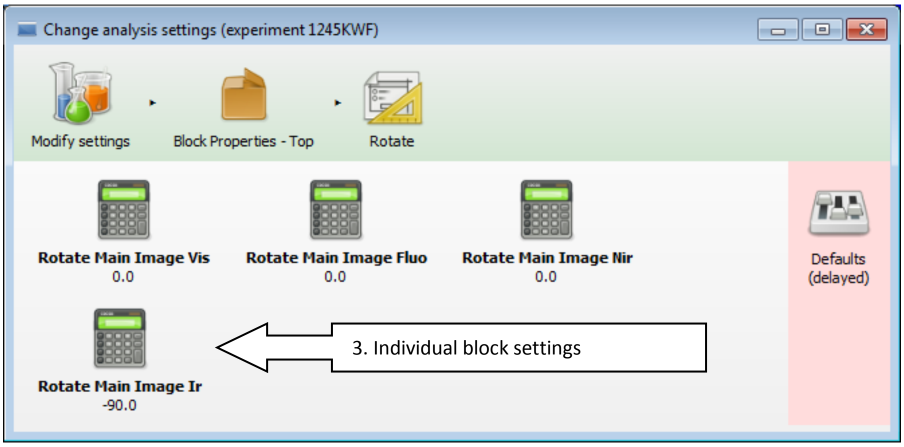

4 Navigating the pipeline settings to the block, responsible for the rotation of input images.

If your dataset contains images from several cameras, it is especially important, that the plants are positioned at the exact same position and with the same size within the image. During the analysis pipeline images from the fluorescence and the visible light images are matched on each other and applied to images from NIR or IR cameras. The analysis block ***Align*** provides a number of settings for each camera type, to correct misalignment and differences in the camera zoom. The process of adjusting these settings is eased with the special overlay image display, which can be enabled by enabling the “debug” option of this image analysis block. The overlay display allows the quick re-processing of this pipeline step. Just change any of the settings, described in the following and click the command button ***Update View***, to recalculate the block-output with the changed settings. You may customize the width of the displayed stripes of the two overlaid images, with the three

[13] This command is also directly available from the image list by right-clicking any image and choosing the context menu item “Change Analysis Settings”.

settings[14] (“Debug-Crossfade-F1/F2/F3”). There are two settings in X and Y direction (e.g. “Ir Zoom X” and “Ir Zoom Y”), you should keep the same X and Y setting, different settings are only required if the aspect ratio between the cameras differs. If the plant has the same relative size within the image, but is positioned differently (differing alignment of the camera hardware), this can be corrected for with the “... Shift X and ... Shift Y” settings. It is best, to ensure proper alignment and the same zoom setting before starting an experiment. But if the images are already taken or if remaining small differences need to be corrected for, then these settings should be adjusted.

As you change a setting, it will be saved automatically, and you can repeat the particular analysis with the updated settings by clicking the command button ***Re-run analysis (debug)***. If a particular analysis block should be completely disabled, click ***Export/Modify settings*** ‣ ***Analysis Blocks*** ‣ ***Block*** and add the comment indicator in front of the particular pipeline ID (e.g. “iap.blocks.BlMedianFilterFluo” ➔ “#iap.blocks.BlMedianFilterFluo”). Changing a block is made quite easy using the dialog sequence, as shown in the screenshots of this page:

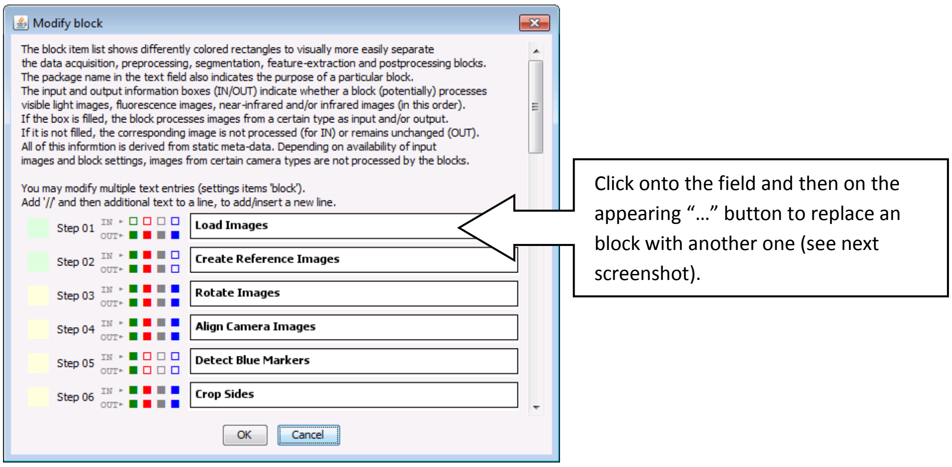

5 Editing the list of pipeline-blocks.

Replacing blocks is supported by graphical command buttons:

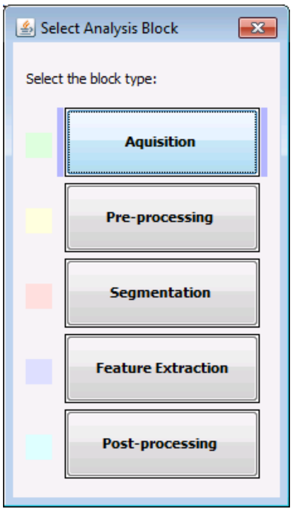

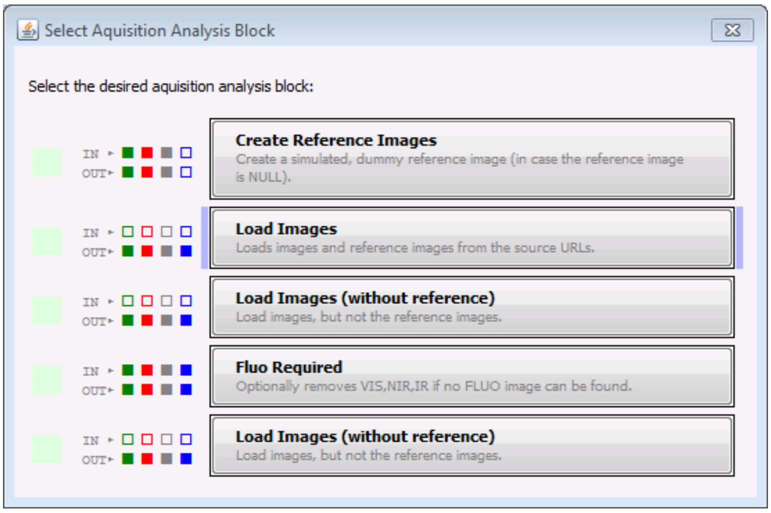

[14] Just try different values and click “Update View” in the overlay image, to yield a good view. The default values for these debug settings are included at the end of these settings names, so that you can quickly change them back if needed.

6 Selecting a new pipeline-block (left: block type selection, right: block selection).

In order to remove, move, delete, or insert new blocks, currently it is required to use the special command text “//” at certain input positions. After entering the command at the right place, the input needs to be confirmed (by clicking OK) and then the dialog can be opened again to add a new block using the graphical selection dialog windows or to confirm other types of edit operations.

Command	Input sequence
**Replace block**	Click the text field and then the appearing “...” button on the right, use the appearing selection dialog windows to choose the new block.
**Disable block for side and top images and all time points**	Add “#” in the beginning of the text field.
**Remove block and leave empty block position**	Click the text field and remove the complete name of the appearing block class name. The block step will remain empty and could be filled with another block, if desired.
**Remove block position**	Remove all text of the block and enter “//”.
**Insert block between two blocks**	Edit the block which should be moved downwards. Add “//” in the beginning of the block and close the dialog. As you open it again, a new empty spot will be created before the previously edited block.

Sometimes it may be desired to have different settings for different time points or for different camera settings, used throughout the experiment time. For example it could be useful, not to remove larger noise objects in the beginning, when the plants are still small and for example no moss has grown on the soil of *Arabidopsis* plants. To differentiate settings for early and/or late stages from settings for the main growth period, click ***Export/Modify settings* ‣ *Separate Settings* ‣ *Early / Late***. Two options appear ***Custom Settings For Early Timepoints*** (yes/no) and ***Early Time Until Time Point*** (number). By modifying the time point number and then enabling the custom setting flag, new separate settings will appear and be used once an image from an early or late time is analysed. It is then possible to modify the block settings or to disable/enable certain blocks for top and/or side settings individually for the early/middle/late time spans.

Once you have checked the correct processing of images from different camera positions, time points, of plants from different genotypes or treatments within your experiment, you can start the analysis of the whole experiment, as described in the next section.

Starting the analysis

After confirming the correct operation of the assigned template, you can start analyzing an experiment. Don’t forget to disable all pipeline “debug” settings, so that no windows are opened during the analysis run. Then navigate back to the desired experiment, and click ***Analysis* ‣ *Perform [Pipeline Name]***. Once the analysis is finished, the experiment result data set is automatically saved and opened. To open it later again, open the particular storage location and choose ***[Storage***

Location]* ‣ *Analysis Results. The results can then be investigated and exported as described in the following.

Result interpretation

The included analysis pipelines by default provide analysis results for visible light, fluorescence, near-infrared and infrared camera data (included by default only for the *Arabidopsis* pipeline template). Within the analysis blocks numeric phenotypic traits are calculated. The result data set contains the source images as so called reference images, the analysis result images (extracted plant from the background), and numeric data. In addition, metadata from the input dataset is retained and used to annotate the result data. The analysis results are calculated and stored for each input image, therefore individually also for each side and top view. To save memory the XLSX export does currently not include the result data for individual side views. Instead for each plant, the average of the side views is exported. The CVS export includes all side view data individually.

Generally, shape and color related properties are calculated. The complete list and description of calculated properties is included in section ***Complete list of calculated traits*** from page 22 on.

Data export

Numeric data (e.g. numeric analysis results) can be exported as a Microsoft Excel Spreadsheet file (XLSX format) (preferred approach) or in text form as a CSV file. To save space, the XLSX export does not include data for individual side views. Open the desired experiment dataset and click ***View/Export Data*** and choose the corresponding export command. Binary files (images) can be exported using the ***Create ZIP file*** and ***Create TAR file*** commands. Also the experiment command button sequence ***Copy* ‣ *To Local File System*** can be used to create a folder hierarchy, containing the experiment images.

The result data may be exported in form of a VANTED data template, see section Importing data from other data domains at page 13 for details on how to enable and access the IAP-Data-Navigator / VANTED functionality. Once this is enabled, data may be opened in the IAP-Data-Navigator user interface. After loading the dataset click ***Put data in 'Experiments' tab***. At the top of the appearing experiment tab click ***Export to Filesystem*** and specify a XLSX target file name (e.g. 'export.xlsx') to export the data in this format. This data table may be loaded again at other installations, but is not the best for data plotting purposes.

Command reference & Background Information

Understanding the command line output

During image analysis the output, printed at the program console looks like this:

5 *Command line output during image analysis*

```
…
18.01.13 21:19>INFO: 22405 ms, 180 p.e., 187 bl/m, 1530/13245 MB (11%) ||
18.01.13 21:19>INFO: 22683 ms, 185 p.e., 187 bl/m, 1627/13245 MB (12%) ||
Pipeline 457: finished block 16/24, took 32 sec., 32873 ms, time: 18.01.13 21:45 (BlockSkeletonize)
```

Most messages are printed with the current date and time at the beginning. After the “>” character beginning with “INFO:” information messages are printed. Error messages begin with “ERROR:”.

In the given example, the analysis progress is printed. The first information is the number of milliseconds for the execution of 5 image analysis pipelines (in this case about 22 seconds). The next number “p.e.” indicates, how many analysis pipeline runs have been finished until now. The value “bl/m” means blocks per minute, and indicates how quickly the blocks are processed and therefore how fast the image data is received processed and stored. The next number, e.g. “1530/13245 MB (11%)”, indicates that currently 11% of the maximum RAM which can be utilized by the application is used.

The last example line is printed if the execution of a block takes longer than expected. The analysis block “Skeletonize”, took in this case 32 seconds, which is over the customizable threshold of 30 seconds (***Settings* ‣ *Iap* ‣ *Info-Print-Block-Execution-Time***).

Multithreading

The maximum number of threads can be tuned using the ***Settings* ‣ *System* ‣ *Cpu-N*** value. If within this setting group the setting ***Cpu-Use-Half-N*** is enabled, half of the indicated number is used. The values are pre-initialized, with the actual number of detected CPU cores. If more than 6 CPU cores are detected, by default it is assumed, that the CPU uses virtual cores, and only half of the CPU cores are utilized, as the virtual cores add only little performance boost, by requiring more available memory for the application, as more images would need to be processed in parallel. These settings can therefore be adjusted in special cases, e.g. if a very large amount of memory is available and used, more cores can be utilized.

Appendix

List of tables

1 *Settings ‣ Lt Db ‣ Image File Transfer Settings* 7

2 *MongoDB example start script (for Linux)* 8

3 *Startup-command for the IAP watch service* 10

4 *Example metadata table* 12

5 *Command line output during image analysis* 19

List of images

1 The three user interface sections of IAP 6

2 Using the IAP-Data-Navigator function to load and process data from other data domains. 13

3 Image analysis pipeline results (displayed is the first step within the pipeline) for a particular snapshot. The images from the different camera types are displayed in the following order: visible light, fluorescence, near-infrared and infrared. If a snapshot contains a subset of these, the corresponding part stays empty. The lower part is used to display reference images in the beginning and intermediate results during the pipeline processing. 15

4 Navigating the pipeline settings to the block, responsible for the rotation of input images. 16

5 Editing the list of pipeline-blocks. 17

6 Selecting a new pipeline-block (left: block type selection, right: block selection). 18

Complete list of calculated traits

When exporting numeric analysis results in CVS or XSLX form, a data table with a larger number of columns is generated. In the following five major groups and the corresponding analysis blocks are shown:

1. Meta data (this page)
2. Traits which are calculated only for side camera images (page 23)
    a. BlBlueMarkerDetection (page 23)
    b. BlCalcWidthAndHeight (page 24)
    c. BlConvexHull (page 24)
3. Traits which are calculated only for top camera images (page 25)
    a. BlCalcMainAxis (page 25)
4. Traits which are calculated in the same way for top and side images (page 25)
    a. BlCalcLeafTips (page 26)
    b. BlSkeletonizeVisOrFluo (page 26)
    c. BlConvexHull (page 27)
    d. BlCalcIntensity (page 30)
5. Calculations which take into account side and top camera images (page 35)
    a. BlConvexHull (page 35)

Meta data columns

Column Name	Description
Angle	For values greater than or equal to 0, the plant rotation angle from the side view. Value -720 stands for the sample average, including data from every side or top view (if applicable to the particular trait).
Plant ID	The identifier of the plant ID (corresponds to the plant carrier). If the image contains several plants, and the processing of these parts has been performed independently, the particular image segment is indicated with the suffix '_x', where 'x' is a whole number, beginning with zero.
Condition	A combination of available metadata information for the different experimental factors (genotypes, treatments).
Species	The plant species (user provided metadata).
Genotype	The plant genotype (user provided metadata).
Variety	The plant species variety (user provided metadata).
GrowthCondition	The growth condition (user provided metadata).
Treatment	The plant treatment (user provided metadata).

Sequence	Metadata field, which can be used to store additional information, e.g. information about a particular experiment, consisting to a sequence of experiment runs.
Day	A textual representation of the sample times including time point as a whole number and time unit (e.g. "day 1").
Time	Exact sample time and date.
Day (Int)	A whole number indicating the relative sample time (e.g. day "5").
Day (Float)	A floating point number, indicating the relative sample time. It is constructed from the whole day information and the relative sample time within the day. E.g. for day 3 10:00 AM, the value would be 3+10/24.
Weight A (g)	If weight data is available, this value contains the measured weight before the watering.
Weight B (g)	If weight data is available, this value contains the measured weight after the watering.
Water (weight-diff)	If weight data is available, this value is the weight difference, observed as the result of the watering.
Water (sum of day)	If plants are watered several times a day, this value is of help to more easily and directly access the daily watering amount for a plant.

Data columns calculated only for side camera images

Analysis Block: BlBlueMarkerDetection

Column Name	Description
RESULT_VIS_MARKER_POS_1_LEFT_X	If three blue marker points are located in the image at the left and right, this value indicates the position of the top left blue marker (X coordinate).
RESULT_VIS_MARKER_POS_1_RIGHT_X	See above, first blue marker at the right, x-coordinate.
RESULT_VIS_MARKER_POS_2_LEFT_X	See above, second blue marker at the left x-coordinate.
RESULT_VIS_MARKER_POS_2_RIGHT_X	See above, second blue marker at the right x-coordinate.
RESULT_VIS_MARKER_POS_3_LEFT_X	See above, third blue marker at the left x-coordinate.

RESULT_VIS_MARKER_POS_3_RIGHT_X	See above, third blue marker at the right x-coordinate.
mark1.y (percent)	Average relative vertical position of the first top blue markers from left and right.
mark2.y (percent)	Average relative vertical position of the second top blue markers from left and right.
mark3.y (percent)	Average relative vertical position of the second top blue markers from left and right.

Analysis Block: BlCalcWidthAndHeight

Column Name	Description
side.width (px)	Width of the plant, measured as the horizontal distance in pixels from the most left plant pixel to the most right plant pixel.
side.width.norm (mm)	See above, but normalized to mm. Requires detection of blue markers and availability of user provided marker distance.

Analysis Block: BlConvexHull

Column Name	Description
side.vis.area.avg (px^2)	Average of plant pixels from the side images (visible light camera) (value is useful, if data for all side images is exported and the avage of the side areas is not immediately available, otherwise. If the data export is done for the average of the side images, this value is equal to the trait 'side.vis.area').
side.vis.area.avg.wue (px^2/ml/day)	The average side area is used to calculate the "water use efficiency", by taking into account the exact sample time, the increase of side area from the previous sample time to the current sample time and the amount of water applied to the plant during this time. If the watering data does not exactly cover the sample time span, the fraction of the water amount from watering data covering a larger time span around the current relevant time span is calculated and considered. The exact calculation formula will be added to the documentation.
side.vis.area.avg.wue.relative	See above, but a relative value (percentage of growth per

(percent/ml/day)	day and ml of water, instead of increase of pixels per day and ml).
side.vis.area.max (px^2)	The maximum side area (in pixels) from the sample side views.
side.vis.area.median (px^2)	The median of the side area values from the sample side views (in pixels).
side.vis.area.min (px^2)	The minimum side area (in pixels) from the sample side views.

Data columns calculated only for top camera images

Analysis Block: BlCalcMainAxis

Column Name	Description
top.avg_distance_to_center (px)	For side trait description, replace the prefix 'top.' by 'side.' and look up the corresponding trait description.
top.main.axis.normalized.distance.avg (mm)	A centre line is calculated by detecting a line crossing the centre of the image. This line is oriented so that the sum of the distances of the plant pixels to this line is minimal. For maize plants this line orientation corresponds to the main leaf orientation. This value indicates the average distance of the plant pixels to this line. The higher this value, the less oriented are the plant leaves relative to the centre line.
top.main.axis.rotation (degree)	The orientation of the line (in degree), 0 indicates horizontal orientation (when looking at the top-image), 90 means orientation from top to bottom (when looking at the image).

Data columns calculated for top and side camera images

All of the data columns listed in the following start with “top.” or “side.”. The data is calculated accordingly from either the top or side image.

Analysis Block: BlCalcLeafTips

Column Name (top. / side. omitted)	Description
side. leaf.count|SUSAN_corder_detection<br><br>*side. leaf.count.[median /max/up/down] |SUSAN_corder_detection*	Estimated leaf count, according to the SUSAN corner detection algorithm. Be aware, that for example the Maize stem may be recognized as a “leaf”, because all

	sharp endpoints appear similar to leaves. You may safely subtract 1 from the calculated value, to get a more direct estimate of the actual leaf count.<br><br>The "median" value represents the median of the detected leaf counts from all side views. The maximum accordingly the maximum count. The up and down count represents the average from side view images of leaves that point up and down accordingly.
bloom.count (tassel)	After skeletonization of the image, this number indicates the number of end points of plant segments, identified as part of a maize bloom.

Analysis Block: BlSkeletonizeVisOrFluo

Column Name (top. / side. omitted)	Description
bloom (0/1)	If a maize bloom has been detected, the value 1 is stored, if no bloom is detected, a 0 is stored.
bloom.count (tassel)	After skeletonization of the image, this number indicates the number of end points of plant segments, identified as part of a maize bloom.
leaf.count (leaves)	Number of end points of the plant skeleton (no minimum branch length).
leaf.width.whole.max	Maximum leaf width in pixels.
leaf.width.whole.max.norm	See above, but normalized to mm. Requires detection of blue markers and availability of user provided marker distance.
leaf.count (leaf)	Number of end points of the plant skeleton (no minimum branch length).
leaf.width.whole.max	Maximum leaf width in pixels.
leaf.width.whole.max.norm	See above, but normalized to mm. Requires detection of blue markers and availability of user provided marker distance.
bloom (0/1)	If a maize bloom has been detected, the value 1 is stored, if no bloom is detected, a 0 is stored.

bloom.count (tassel)	After skeletonization of the image, this number indicates the number of end points of plant segments, identified as part of a maize bloom.

Analysis Block: BlConvexHull

**Column Name (top. / side. omitted)**	**Description**
fluo.area	Projected side area (in pixels) of the plant, from the fluorescence image.
fluo.area.norm	Projected side area (in mm^2 of the plant, from the fluorescence image. Normalised data is only calculated, if blue markers have been detected and the horizontal marker distance is provided by the user.
fluo.border.length	Number of pixels of the plant, connected by at least one side of the pixel to the background (4-neighbourhood).
fluo.border.length.norm	See above, but normalized to mm. Requires detection of blue markers and availability of user provided marker distance.
fluo.compactness.01	4 * Math.PI / (borderPixels * borderPixels / filledArea)
fluo.compactness.16	borderPixels * borderPixels / filledArea
fluo.hull.area	Area (in pixels) of the convex hull, which is the shortest convex line drawing around the plant.
fluo.hull.area.norm	See above, but normalized to mm^2. Requires detection of blue markers and availability of user provided marker distance.
fluo.hull.circularity	Indicates similarity to a circle, ranges between 0 and 1. A circular object has value 1.
fluo.hull.circumcircle.d	Diameter of the smallest circle drawn around the plant.
fluo.hull.circumcircle.d.norm	See above, but normalized to mm. Requires detection of blue markers and availability of user provided marker distance.
fluo.hull.fillgrade	Number of pixels of the plant relative to the area of the convex hull. May be formatted as percentage values in Excel (e.g. 20%), CSV exported data is displayed unformatted, e.g. 0.2.
fluo.hull.pc1	Largest distance (in pixels) of any two pixels of the plant.

fluo.hull.pc1.norm	See above, but normalized to mm. Requires detection of blue markers and availability of user provided marker distance.
fluo.hull.pc2	If a line connects the two most far from each other situated plant pixels is drawn, this number indicates the sum of the maximum distances of other plant pixels from the left and right of this line.
fluo.hull.pc2.norm	See above, but normalized to mm. Requires detection of blue markers and availability of user provided marker distance.
fluo.hull.points	Number of edge points of the convex hull around the plant.
vis.area	Projected side area (in pixels) of the plant, from the visible light image.
vis.area.avg (px^2)	Average of plant pixels from the side images (visible light camera) (value is useful, if data for all side images is exported and the average of the side areas is not immediately available, otherwise. If the data export is done for the average of the side images, this value is equal to the trait 'side.vis.area').
vis.area.avg.wue (px^2/ml/day)	The average side area is used to calculate the "water use efficiency", by taking into account the exact sample time, the increase of side area from the previous sample time to the current sample time and the amount of water applied to the plant during this time. If the watering data does not exactly cover the sample time span, the fraction of the water amount from watering data covering a larger time span around the current relevant time span is calculated and considered. The exact calculation formula will be added to the documentation.
vis.area.avg.wue.relative (percent/ml/day)	See above, but a relative value (percentage of growth per day and ml of water, instead of increase of pixels per day and ml).
vis.area.max (px^2)	The maximum side area (in pixels) from the sample side views.
vis.area.median (px^2)	The median of the side area values from the sample side views (in pixels).
vis.area.min (px^2)	The minimum side area (in pixels) from the sample side

	views.
vis.area.norm	See 'side.vis.area', but normalized to mm. Requires detection of blue markers and availability of user provided marker distance.
vis.border.length	Number of pixels of the plant (visible light image), connected by at least one side of the pixel to the background (4-neighbourhood).
vis.border.length.norm	See above, but normalized to mm. Requires detection of blue markers and availability of user provided marker distance.
vis.compactness.01	4 * Math.PI / (borderPixels * borderPixels / filledArea)
vis.compactness.16	borderPixels * borderPixels / filledArea
vis.hull.area	Area (in pixels) of the convex hull, which is the shortest convex line drawing around the plant.
vis.hull.area.norm	See above, but normalized to mm^2. Requires detection of blue markers and availability of user provided marker distance.
vis.hull.circularity	Indicates similarity to a circle, ranges between 0 and 1. A circular object has value 1.
vis.hull.circumcircle.d	Diameter of the smallest circle drawn around the plant.
vis.hull.circumcircle.d.norm	See above, but normalized to mm. Requires detection of blue markers and availability of user provided marker distance.
vis.hull.fillgrade	Number of pixels of the plant relative to the area of the convex hull. May be formatted as percentage values in Excel (e.g. 20%), CSV exported data is displayed unformatted, e.g. 0.2.
vis.hull.pc1	Largest distance (in pixels) of any two pixels of the plant.
vis.hull.pc1.norm	See above, but normalized to mm. Requires detection of blue markers and availability of user provided marker distance.
vis.hull.pc2	If a line connects the two most far from each other situated plant pixels is drawn, this number indicates the sum of the maximum distances of other plant pixels from

	the left and right of this line.
vis.hull.pc2.norm	See above, but normalized to mm. Requires detection of blue markers and availability of user provided marker distance.
vis.hull.points	Number of edge points of the convex hull around the plant.

Analysis Block: BlCalcIntensity

**Column Name (top. / side. omitted)**	**Description**
fluo.intensity.average (relative)	Deprecated. The same as 'side/top.fluo.intensity.chlorophyl.average (relative)'. Trait may be removed at a later time point.
fluo.intensity.chlorophyl.average (relative)	A relative indicator of the red fluorescence intensity, not taking into account brightness but only the color hue (red = highest intensity, yellow = no intensity). Detailed information will be added to the documentation.
fluo.intensity.chlorophyl.sum	The sum of the intensities of each pixel, calculated as above.
fluo.intensity.classic.average	A relative indicator of the red fluorescence intensity, taking into account brightness and colour hue (red = highest intensity, yellow = no intensity, bright = high intensity, dark = low intensity). Detailed information will be added to the documentation. Calculation formula: (1 - red / (255 + green)) / 0.825
fluo.intensity.classic.sum	The sum of the intensities of each pixel, calculated as above.
fluo.intensity.phenol.average (relative)	A relative indicator of the yellow fluorescence intensity, not taking into account brightness but only the color hue (red = no intensity, yellow = high intensity). Detailed information will be added to the documentation.
fluo.intensity.phenol.chlorophyl.ratio (c/p)	The ratio of trait 'side.fluo.intensity.chlorophyl.sum' and 'side.fluo.intensity.phenol.sum'. For the top

	value, the according top values are used.
fluo.intensity.phenol.plant_weight	-
fluo.intensity.phenol.plant_weight_drought_loss	-
fluo.intensity.phenol.sum	The sum of the yellow fluorescence intensities of each pixel, calculated as described for 'side/fluo.intensity.phenol.average (relative)'.
ndvi (relative)	ndvi = (averageNir - averageVisR) / (averageNir + averageVisR)
ndvi.vis.blue.intensity.average (relative)	Average intensity of the blue channel of the plant pixels in the visible light image.
ndvi.vis.green.intensity.average (relative)	Average intensity of the green channel of the plant pixels in the visible light image.
ndvi.vis.red.intensity.average (relative)	Average intensity of the red channel of the plant pixels in the visible light image.
nir.filled.percent	Number of plant pixels divided by number of overall pixels in the near-infrared image. If half of the image is filled by the plant, the value would be 0.5. If 10% of the image is filled by the plant, the value would be 0.1. Excel table display may be formatted to show
nir.filled.pixels	Number of plant pixels from the near-infrared image.
nir.intensity.average (relative)	Average intensity (1-brightness) of the plant near-infrared pixels.
nir.intensity.sum	Average intensity multiplied by number of plant pixels (for both, see above).
nir.skeleton.intensity.average (relative)	Average intensity (1-brightness) of the plant skeleton pixels of the near-infrared image.
vis.hsv.dgci.average	Numeric indication on how 'dark green' the plant appears, taking into account hue, saturation and brightness. Differs from calculation in other sources in that the higher the saturation, the assumption is that the plant appears greener, and thus the value is increasing in this case. The column 'side.vis.hsv.dgci_orig.average'

	corresponds to the unintuitive but documented calculation of this trait.
vis.hsv.dgci_orig.average	Numeric indication on how 'dark green' the plant appears, taking into account hue, saturation and brightness. Uses the original calculation scheme, where higher saturation is said to indicate less green. The column 'side.vis.hsv.dgci.average' corresponds to the more intuitive interpretation that less saturation means also less green appearance.
vis.hsv.h.average	The plant average hue in the HSV/HSB colour space. The value range is normalized to a minimum of 0 and a maximum of 1. Value one corresponds to non-technical descriptions of 360 degrees for this colour space.
vis.hsv.h.kurtosis	The 'kurtosis' of the hue values of the plant pixels. 'kurtosis' is a statistical term, indicating the 'peakedness' of the value distribution. The documentation will include a more complete description of this trait in the future; see reference literature for full details.
vis.hsv.h.skewness	The 'skewness' of the hue values of the plant pixels. 'skewness' is a statistical term, indicating the tendency of the value distribution to lean to one side of the value range. The documentation will include a more complete description of this trait in the future; see reference literature for full details.
vis.hsv.h.stddev	The standard deviation of the hue values of the plant pixels. The lower this value, the more uniform is the plant colour.
vis.hsv.s.average	The plant average saturation in the HSV/HSB colour space. A high value indicates more 'intensive' colours, low values indicate pale colours. This value ranges from 0 to 1, other software or references may utilize different ranges, e.g. a maximum of 100.
vis.hsv.s.kurtosis	The 'kurtosis' of the saturation values of the plant pixels. 'kurtosis' is a statistical term, indicating the 'peakedness' of the value distribution. The

	documentation will include a more complete description of this trait in the future; see reference literature for full details.
vis.hsv.s.skewness	The 'skewness' of the saturation values of the plant pixels. 'skewness' is a statistical term, indicating the tendency of the value distribution to lean to one side of the value range. The documentation will include a more complete description of this trait in the future; see reference literature for full details.
vis.hsv.s.stddev	The standard deviation of the saturation values of the plant pixels. The lower this value, the more uniform is the saturation of the plant colours.
vis.hsv.v.average	The plant average brightness in the HSV/HSB colour space. This value ranges from 0 to 1, other software or references may utilize different ranges, e.g. a maximum of 100.
vis.hsv.v.kurtosis	The 'kurtosis' of the brightness values of the plant pixels. 'kurtosis' is a statistical term, indicating the 'peakedness' of the value distribution. The documentation will include a more complete description of this trait in the future; see reference literature for full details.
vis.hsv.v.skewness	The 'skewness' of the brightness values of the plant pixels. 'skewness' is a statistical term, indicating the tendency of the value distribution to lean to one side of the value range. The documentation will include a more complete description of this trait in the future; see reference literature for full details.
vis.hsv.v.stddev	The standard deviation of the brightness values of the plant pixels. The lower this value, the more uniform is the plant brightness.
vis.lab.a.kurtosis	The 'kurtosis' of the a-values in the L*a*b* colour space of the plant pixels. 'kurtosis' is a statistical term, indicating the 'peakedness' of the value distribution. The documentation will include a more complete description of this trait in the future; see reference literature for full details.

vis.lab.a.mean	The plant average a-value of the plant pixel colours in the L*a*b* colour space. Small values indicate green while high values indicate magenta. This value ranges from 26 to 225, other software or references may utilize different ranges, e.g. higher negative together with higher positive values.
vis.lab.a.skewness	The 'skewness' of the a-values in the L*a*b* colour space of the plant pixels. 'skewness' is a statistical term, indicating the tendency of the value distribution to lean to one side of the value range.
vis.lab.a.stddev	The standard deviation of the a-values in the L*a*b* colour space of the plant pixels. The lower this value, the more uniform is the plant colour.
vis.lab.b.kurtosis	The 'kurtosis' of the b-values in the L*a*b* colour space of the plant pixels. 'kurtosis' is a statistical term, indicating the 'peakedness' of the value distribution.
vis.lab.b.mean	The plant average b-value of the plant pixel colours in the L*a*b* colour space. Small values indicate blue and high values indicate yellow. This value ranges from 8 to 223, other software or references may utilize different ranges, e.g. higher negative values together with higher positive values.
vis.lab.b.skewness	The 'skewness' of the b values in the L*a*b* colour space of the plant pixels. 'skewness' is a statistical term, indicating the tendency of the value distribution to lean to one side of the value range.
vis.lab.b.stddev	The standard deviation of the b values in the L*a*b* colour space of the plant pixels. The lower this value, the more uniform is the plant colour.
vis.lab.l.kurtosis	The 'kurtosis' of the brightness values L in the L*a*b* colour space of the plant pixels. 'kurtosis' is a statistical term, indicating the 'peakedness' of the value distribution.
vis.lab.l.mean	The plant average brightness value of the plant

	pixel colours in the L*a*b* colour space. Small values indicate low and high values high brightness. This value ranges from 0 to 255, other software or references may utilize different ranges.
vis.lab.l.skewness	The 'skewness' of the brightness values L in the L*a*b* colour space of the plant pixels. 'skewness' is a statistical term, indicating the tendency of the value distribution to lean to one side of the value range.
vis.lab.l.stddev	The standard deviation of the brightness values L in the L*a*b* colour space of the plant pixels. The lower this value, the more uniform is the plant brightness.
vis.stress.hue.brown2green	Proportion of brown colour plant pixels (histogram bin 2) divided by the count of green colour pixels (bins 4 to 7).
vis.stress.hue.red2green	Proportion of red colour plant pixels (histogram bins 0 and 1) divided by the count of green colour pixels (bins 4 to 7).
vis.stress.hue.yellow2green	Proportion of yellow colour plant pixels (histogram bin 3) divided by the count of green colour pixels (bins 4 to 7).

Data columns calculated by combining data from top and side camera images

Analysis Block: BlConvexHull

Column Name)	Description
volume.fluo.iap (px^3)	A approximation of the projected plant volume, calculated by assuming the plant to be a square box. The top and bottom planes are assumed to be quadratic in this approximation. The side plane area is assumed to be equal to the average of the projected side views ('side.fluo.area' for each side view), and the top and bottom plane area is assumed to be equal to the projected top view area ('top.fluo.area'). The calculation formula is therefore volume.fluo.iap = sqrt(avg(mean(side.fluo.area)^2 * mean(top.fluo.area)).

volume.fluo.plant_weight.iap	-
volume.vis.area090T (px^2)	The sum of two side areas (best fitting to 0 and 90 degree) and the top area.
volume.vis.iap (px^3)	Calculated the same way as 'volume.fluo.iap', but taking the area values from the visible light images.
volume.vis.iap.relative (percent/day)	A relative growth rate of the plant volume per day (formatted and displayed as percentage).
volume.vis.iap.wue (px^3/ml/day)	The plant volume growth rate per ml water and day. For details on how the water consumption and time is considered, see 'side.vis.area.avg.wue (px^2/ml/day)'.
volume.vis.iap_max (px^3)	The volume calculated similar to 'volume.fluo.iap (px^3)', but taking into account not the average of the projected side views, but the maximum of the projected side view areas.
volume.vis.lt (px^3)	Math.sqrt(sideArea_for_angleNearestTo0 * sideArea_for_angleNearestTo90 * avgTopArea)
volume.vis.prism (px^3)	The volume of the plant, when assuming the plant to be a triangular prism. Taking into account three side views and the top view area.

Keywords

—[—

[time] 10

—A—

Add to Clipboard 12

Analysis Results 19

Angle..22

Annotation Image..14

Automated copy-service 11

—B—

BlBlueMarkerDetection................................23

BlCalcIntensity 30

BlCalcLeafTips...25

BlCalcMainAxis 25

BlCalcWidthAndHeight 24

BlConvexHull....................................24, 27, 35

BlSkeletonizeVisOrFluo................................26

bookmark 6

button list 6

—C—

Change analysis settings...............................16

Condition 22

Create TAR file...19

Create ZIP file 19

CSV...11, 12, 13, 19

—D—

Data columns...................................23, 25, 35

Day...23

Day (Float) 23

Day (Int) 23

Defaults (delayed).......................................14

Disable block...18

—E—

Experiment progress monitoring........ 9, 10, 11

—F—

FTP 7

—G—

Genotype 22

growing-degree-days

GDD...12

GrowthCondition 22

—H—

host name 7

—I—

iap.ini 6

image analysis pipeline........................... 5, 14

image analysis templates............................14

Image File Transfer 7

Insert block 18

IP cameras.. 10, 11

—L—

Lemna DBImportExport 11

Linux...5

Load LT File Export......................................12

—M—

Mac OS X..5

mark1.y (percent) 24

mark2.y (percent) 24

mark3.y (percent) 24

Meta data columns 22

MongoDB 7, 8, 9, 11, 12, 13, 14

monitoring 10

Multi-Document-Interface

MDI 6

Multithreading 20

—N—

New Window 6

—P—

password 7

Plant ID 22

program folder 6

Publish Desktop 11

—R—

RAM 5

Reference Image 14

Remove block 18

Replace block 18

Re-run analysis (debug) 17

RESULT_VIS_MARKER_POS_1_LEFT_X 23

RESULT_VIS_MARKER_POS_1_RIGHT_X 23

RESULT_VIS_MARKER_POS_2_LEFT_X 23

RESULT_VIS_MARKER_POS_2_RIGHT_X 23

RESULT_VIS_MARKER_POS_3_LEFT_X 23

RESULT_VIS_MARKER_POS_3_RIGHT_X 24

—S—

SCP 7

Screenshot Storage 11

Separate Settings ▸ Early / Late 18

Sequence 23

Show Complete Snapshot Set 15

Show Image 14

side.vis.area.avg (px^2) 24

side.vis.area.avg.wue (px^2/ml/day) 24

side.vis.area.avg.wue.relative (percent/ml/day) 24

side.vis.area.max (px^2) 25

side.vis.area.median (px^2) 25

side.vis.area.min (px^2) 25

side.width (px) 24

side.width.norm (mm) 24

side/top.bloom (0/1) 26

side/top.bloom.count (tassel) 26, 27

side/top.fluo.area 27

side/top.fluo.area.norm 27

side/top.fluo.border.length 27

side/top.fluo.border.length.norm 27

side/top.fluo.compactness.01 27

side/top.fluo.compactness.16 27

side/top.fluo.hull.area 27

side/top.fluo.hull.area.norm 27

side/top.fluo.hull.circularity 27

side/top.fluo.hull.circumcircle.d 27

side/top.fluo.hull.circumcircle.d.norm 27
side/top.fluo.hull.fillgrade............................27
side/top.fluo.hull.pc1 27
side/top.fluo.hull.pc1.norm 28
side/top.fluo.hull.pc2 28
side/top.fluo.hull.pc2.norm 28
side/top.fluo.hull.points...............................28
side/top.fluo.intensity.average (relative) 30
side/top.fluo.intensity.chlorophyl.average (relative) 30
side/top.fluo.intensity.chlorophyl.sum.........30
side/top.fluo.intensity.classic.average..........30
side/top.fluo.intensity.classic.sum...............30
side/top.fluo.intensity.phenol.average (relative) 30
side/top.fluo.intensity.phenol.chlorophyl.ratio (c/p) 30
side/top.fluo.intensity.phenol.plant_weight 31
side/top.fluo.intensity.phenol.plant_weight_drought_loss...31
side/top.fluo.intensity.phenol.sum..............31
side/top.leaf.count (leaf)..............................26
side/top.leaf.count (leaves) 26
side/top.leaf.width.whole.max 26
side/top.leaf.width.whole.max.norm 26
side/top.ndvi (relative)................................31
side/top.ndvi.vis.blue.intensity.average (relative) 31
side/top.ndvi.vis.green.intensity.average (relative) 31
side/top.ndvi.vis.red.intensity.average (relative).. 31
side/top.nir.filled.percent............................ 31
side/top.nir.filled.pixels............................... 31
side/top.nir.intensity.average (relative)....... 31
side/top.nir.intensity.sum 31
side/top.nir.skeleton.intensity.average (relative).. 31
side/top.vis.area 28
side/top.vis.area.avg (px^2) 28
side/top.vis.area.avg.wue (px^2/ml/day...... 28
side/top.vis.area.avg.wue.relative (percent/ml/day) 28
side/top.vis.area.max (px^2) 28
side/top.vis.area.median (px^2).................. 28
side/top.vis.area.min (px^2)........................ 28
side/top.vis.area.norm 29
side/top.vis.border.length........................... 29
side/top.vis.border.length.norm 29
side/top.vis.compactness.01 29
side/top.vis.compactness.16 29
side/top.vis.hsv.dgci.average 31
side/top.vis.hsv.dgci_orig.average 32
side/top.vis.hsv.h.average........................... 32
side/top.vis.hsv.h.kurtosis........................... 32
side/top.vis.hsv.h.skewness 32
side/top.vis.hsv.h.stddev............................. 32
side/top.vis.hsv.s.average 32
side/top.vis.hsv.s.kurtosis 32

side/top.vis.hsv.s.skewness 33
side/top.vis.hsv.s.stddev 33
side/top.vis.hsv.v.average 33
side/top.vis.hsv.v.kurtosis 33
side/top.vis.hsv.v.skewness 33
side/top.vis.hsv.v.stddev 33
side/top.vis.hull.area 29
side/top.vis.hull.area.norm 29
side/top.vis.hull.circularity 29
side/top.vis.hull.circumcircle.d 29
side/top.vis.hull.circumcircle.d.norm 29
side/top.vis.hull.fillgrade 29
side/top.vis.hull.pc1 29
side/top.vis.hull.pc1.norm 29
side/top.vis.hull.pc2 29
side/top.vis.hull.pc2.norm 30
side/top.vis.hull.points 30
side/top.vis.lab.a.kurtosis 33
side/top.vis.lab.a.mean 34
side/top.vis.lab.a.skewness 34
side/top.vis.lab.a.stddev 34
side/top.vis.lab.b.kurtosis 34
side/top.vis.lab.b.mean 34
side/top.vis.lab.b.skewness 34
side/top.vis.lab.b.stddev 34
side/top.vis.lab.l.kurtosis 34
side/top.vis.lab.l.mean 34
side/top.vis.lab.l.skewness 35
side/top.vis.lab.l.stddev 35
side/top.vis.stress.hue.brown2green 35
side/top.vis.stress.hue.red2green 35
side/top.vis.stress.hue.yellow2green 35
Snapshot-Export 11
Species 22
Starttime-H 11

—T—

terminal 5
Time 23
top.avg_distance_to_center (px) 25
top.main.axis.normalized.distance.avg (mm) 25
top.main.axis.rotation (degree) 25
Treatment 22

—U—

Update View 16, 17
User Interface 6
user name 7

—V—

VANTED 13
Variety 22
VFS 8
Virtual File System 8
volume.fluo.iap (px^3) 35
volume.fluo.plant_weight.iap 36
volume.vis.area090T (px^2) 36
volume.vis.iap (px^3) 36
volume.vis.iap.relative (percent/day) 36

volume.vis.iap.wue (px^3/ml/day)...............36
volume.vis.iap_max (px^3)...........................36
volume.vis.lt (px^3) 36
volume.vis.prism (px^3) 36

—W—

watch 10
Watch-services 10, 11
Water (sum of day).......................................23
Water (weight-diff) 23
webcam 10
Weight A (g) 23
Weight B (g) 23
Windows 5
working-directory 5

—X—

XLSX... 12, 13, 19

Revision History

V1: Dec 2012 – Jan 2013: Initial version of document, May 2013: Added list of trait descriptions and final check for first public revision.

V2: July 2013: Trait normalization detailed (conversion of trait values from pixels to mm).

V3: November 2013: Updated help on configuration of monitoring settings and pipeline settings to reflect changes of IAP V1.1.

V4: November 2013: Added leaf count block result details.

Legal and Trademarks

IAP – The Integrated Analysis Platform is Copyright © 2010-2013 IPK Gatersleben, Group Image Analysis

Disclaimer of Warranties

You acknowledge and agree that the use of IAP is at your sole risk and that the entire risk as to satisfactory quality, performance, accuracy and effort is with you. It cannot be guaranteed, that (1) the functions contained in this software will meet your requirements, that (2) the operation of IAP will be uninterrupted or error-free, or that (3) defects in the software will be corrected.

A number of software libraries, listed within IAP (click "About" and then "Library Licenses" on the bottom of the start screen) have been used during the development of IAP. By using IAP you need to accept and be aware of the terms and conditions of these licenses.

Trademarks

Linux® is the registered trademark of Linus Torvalds in the U.S. and other countries. Windows® is a registered trademark of Microsoft Corporation in the United States and other countries. Mac® and OS X® are trademarks of Apple Inc.. Oracle® and Java™ are registered trademarks of Oracle and/or its affiliates. MongoDB® and Mongo®, and the leaf logo are registered trademarks of 10gen, Inc.. lemnatec is a registered trademark of LemnaTec GmbH.

Other names may be trademarks of their respective owners.
