# Supplementary material for: The ‘PhenoBox’, a flexible, automated, open‐source plant phenotyping solution
Source: New Phytol. 2018 Apr 5;219(2):808–23. doi: 10.1111/nph.15129 (PMC6485332; doi:10.1111/nph.15129)
Supplement: Supplementary file 11 — Notes S4 PhenoBox documentation. [file NPH-219-808-s011.pdf]

The "Phenobox", a flexible, automated, open-source plant phenotyping solution

Angelika Czedik-Eysenberg, Sebastian Seitner, Ulrich Güldener, Stefanie Koemeda, Jakub Jez, Martin Colombini, Armin Djamei

Accepted 22.2.2018

PhenoBox Documentation

Slide	
1	PhenoBox top view
2	PhenoBox side view
3	PhenoBox electronics compartment
4-5	circuit diagrams for the PhenoBox electronics
6	PhenoBox front view open/closed
7	PhenoBox door inside with mechanisms to adjust vertical camera position
8	Pot Adaptor
9	PhenoBox Component list

24 Numbers on images indicate items from the PhenoBox component list

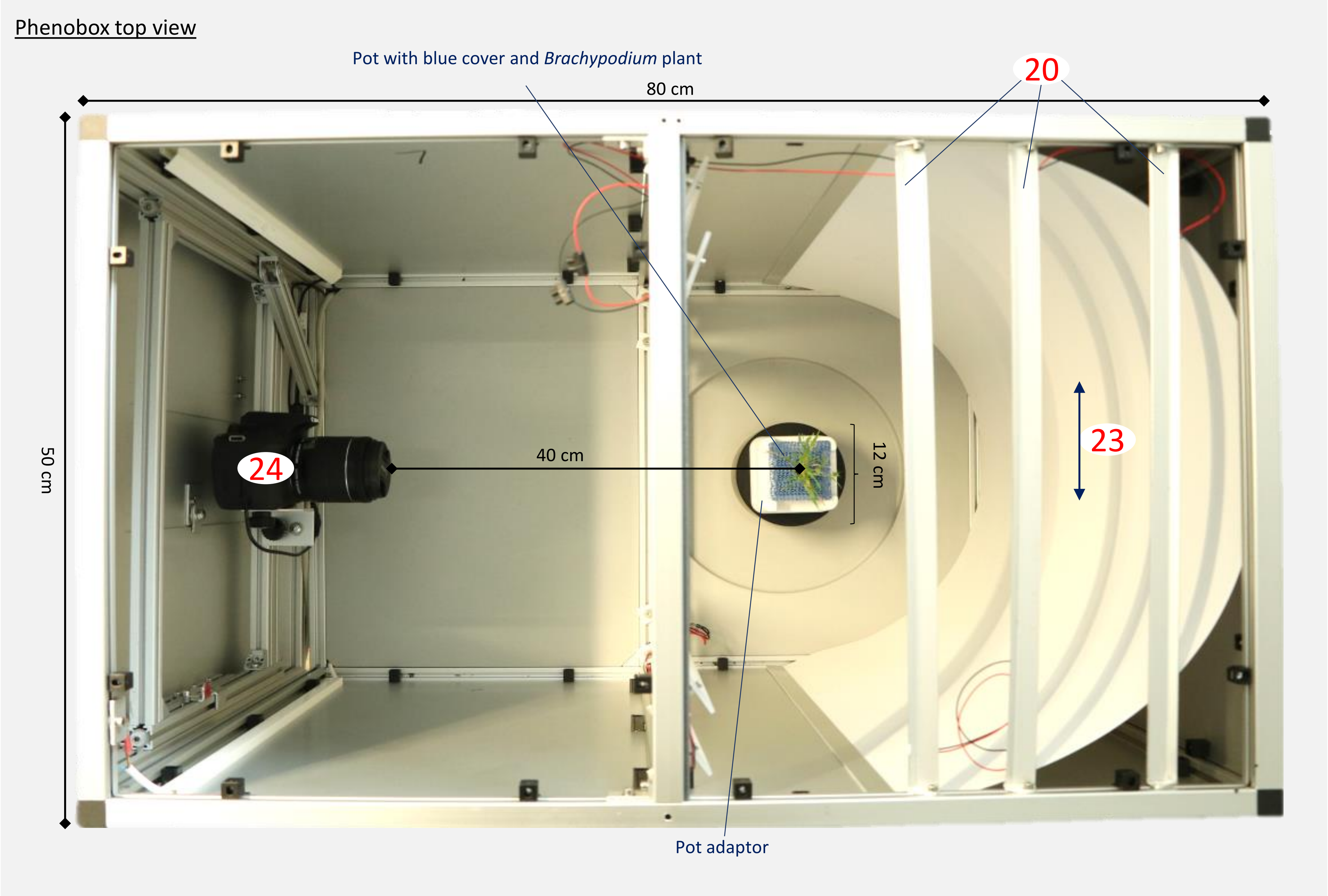

Phenobox top view
Pot with blue cover and *Brachypodium* plant
20
80 cm
50 cm
24
40 cm
12 cm
23
Pot adaptor

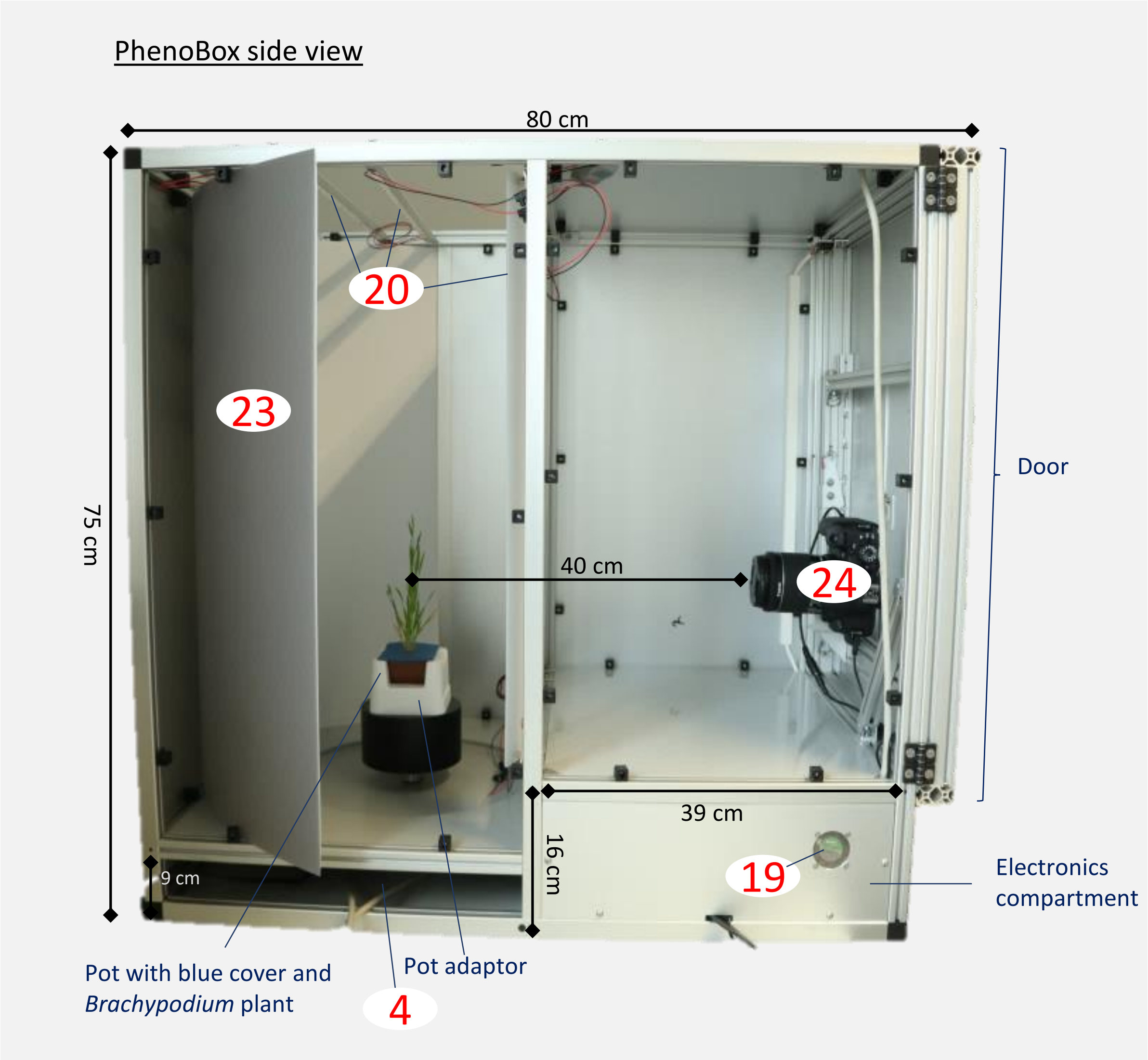

PhenoBox side view
80 cm
75 cm
20
23
40 cm
24
Door
39 cm
16 cm
19
9 cm
Electronics compartment
Pot with blue cover and *Brachypodium* plant
Pot adaptor
4

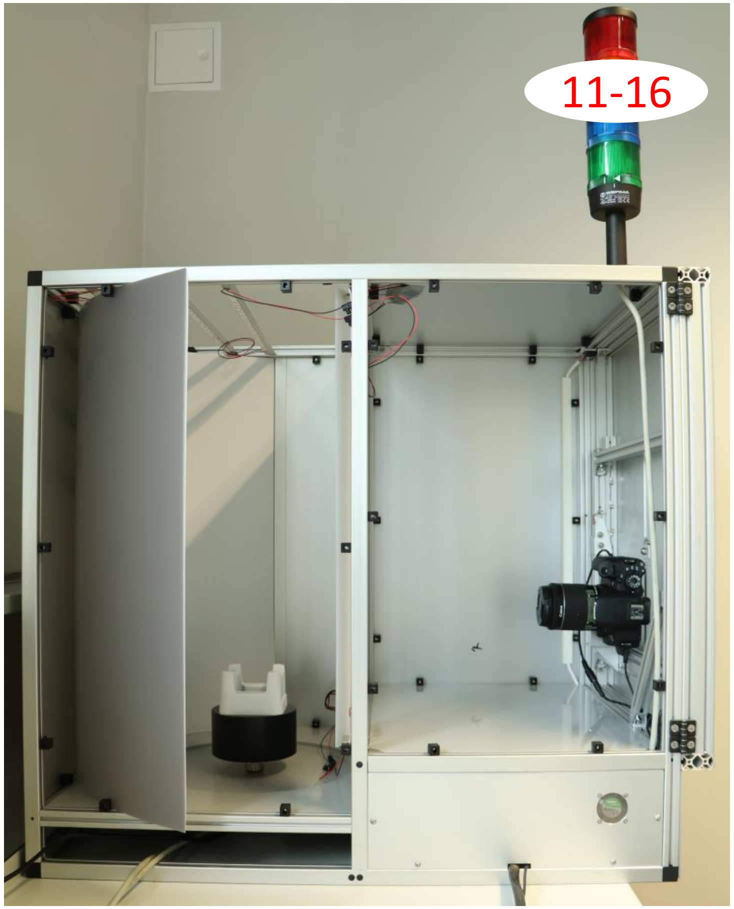

11-16

PhenoBox electronics compartment

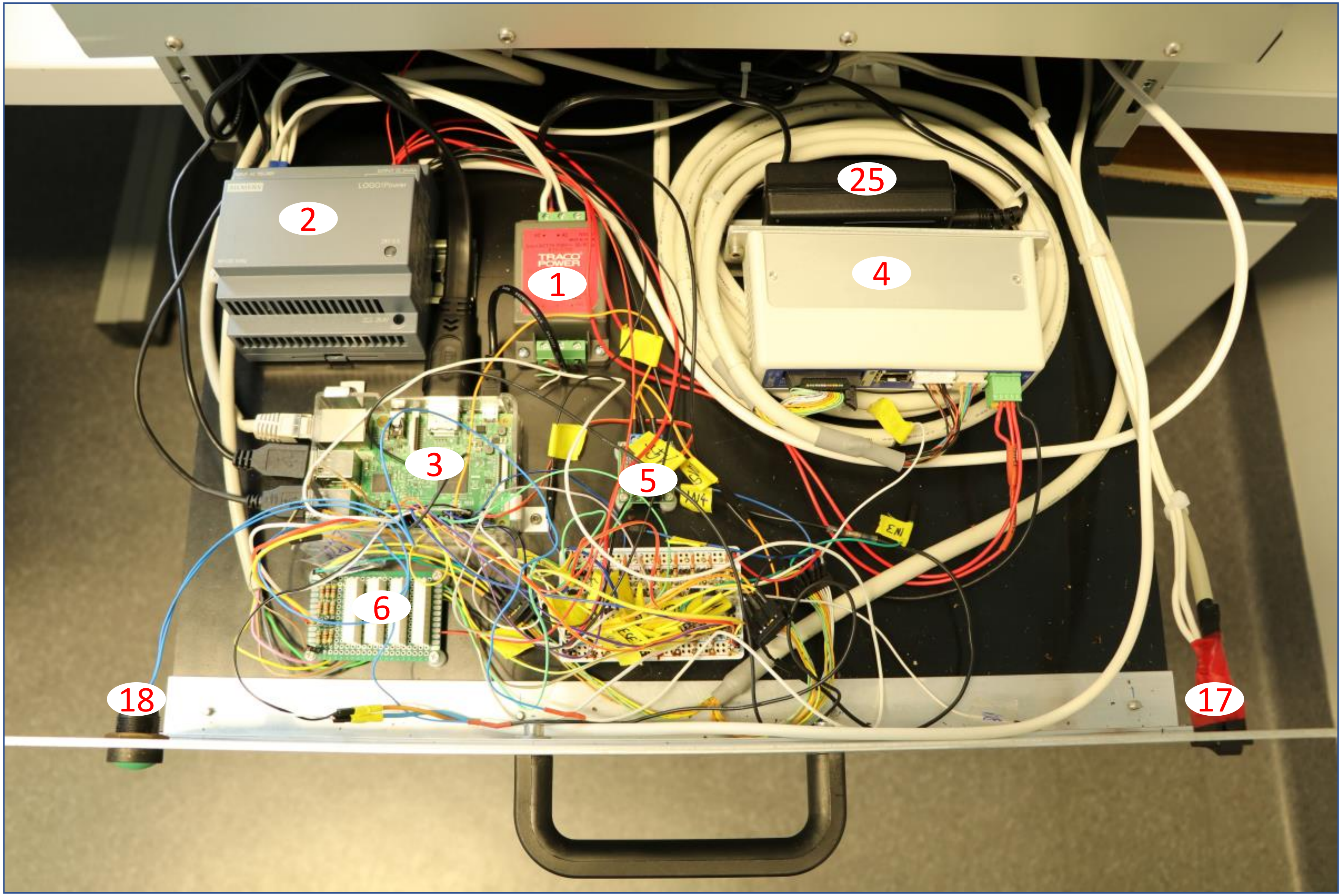

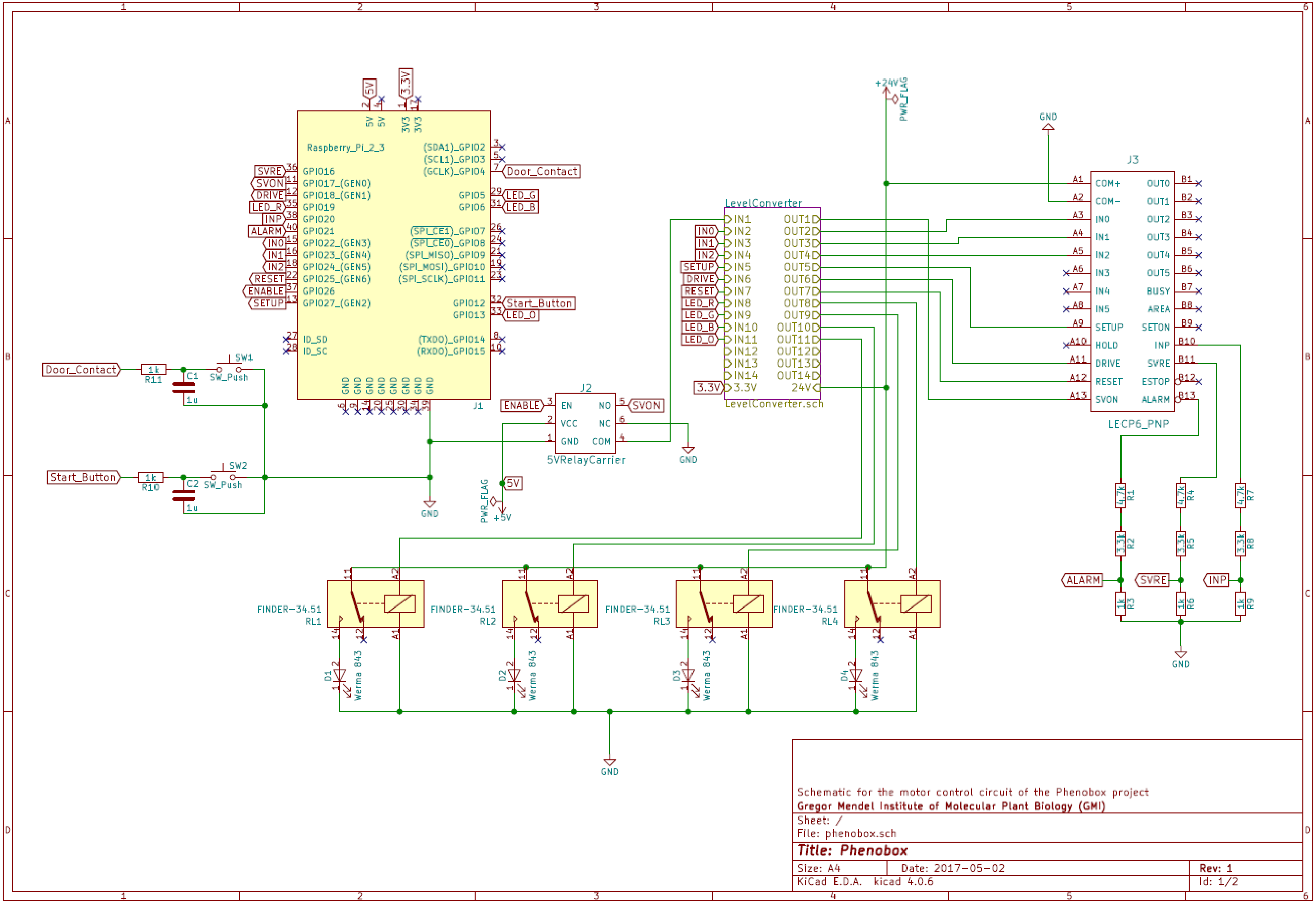

Raspberry_Pi_2_3
J1
5V
3.3V
GPIO16
GPIO17_(GEN0)
GPIO18_(GEN1)
GPIO19
GPIO20
GPIO21
GPIO22_(GEN3)
GPIO23_(GEN4)
GPIO24_(GEN5)
GPIO25_(GEN6)
GPIO26
GPIO27_(GEN2)
ID_SD
ID_SC
(SDA1)_GPIO2
(SCL1)_GPIO3
(GCLK)_GPIO4
GPIO5
GPIO6
(SPI_CE1)_GPIO7
(SPI_CE0)_GPIO8
(SPI_MISO)_GPIO9
(SPI_MOSI)_GPIO10
(SPI_SCLK)_GPIO11
GPIO12
GPIO13
(TXD0)_GPIO14
(RXD0)_GPIO15
SVRE
SVON
DRIVE
LED_R
INP
ALARM
IN0
IN1
IN2
RESET
ENABLE
SETUP
Door_Contact
LED_G
LED_B
Start_Button
LED_O
R11 1k
C1 1u
SW1 SW_Push
R10 1k
C2 1u
SW2 SW_Push
GND
J2
5VRelayCarrier
EN
VCC
GND
NO
NC
COM
PWR_FLAG
+5V
LevelConverter
LevelConverter.sch
IN1 … IN14
OUT1 … OUT14
3.3V
24V
+24V
J3
LECP6_PNP
COM+
COM−
IN0
IN1
IN2
IN3
IN4
IN5
SETUP
HOLD
DRIVE
RESET
SVON
OUT0
OUT1
OUT2
OUT3
OUT4
OUT5
BUSY
AREA
SETON
INP
SVRE
ESTOP
ALARM
R1 4.7k
R2 3.3k
R3 1k
R4 4.7k
R5 3.3k
R6 1k
R7 4.7k
R8 3.3k
R9 1k
FINDER−34.51
RL1
RL2
RL3
RL4
D1
D2
D3
D4
Werma 843
Schematic for the motor control circuit of the Phenobox project
Gregor Mendel Institute of Molecular Plant Biology (GMI)
Sheet: /
File: phenobox.sch
Title: Phenobox
Size: A4
Date: 2017-05-02
Rev: 1
KiCad E.D.A. kicad 4.0.6
Id: 1/2

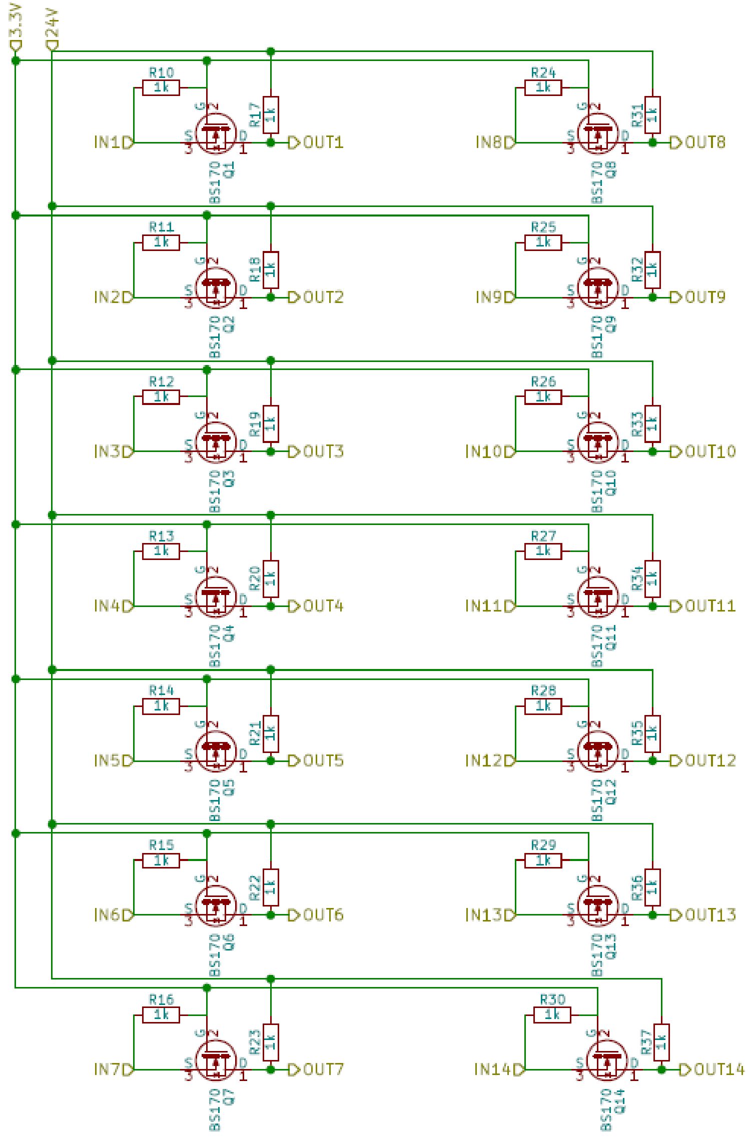

Level converter used to shift 3.3V to 24V

Gregor Mendel Institute of Molecular Plant Biology (GMI)

Sheet: /LevelConverter/

File: LevelConverter.sch

Title: Level Converter

Size: A4 Date: 2017-05-02 **Rev: 1**

KiCad E.D.A. kicad 4.0.6 Id: 2/2

PhenoBox frontal view with opened and closed door

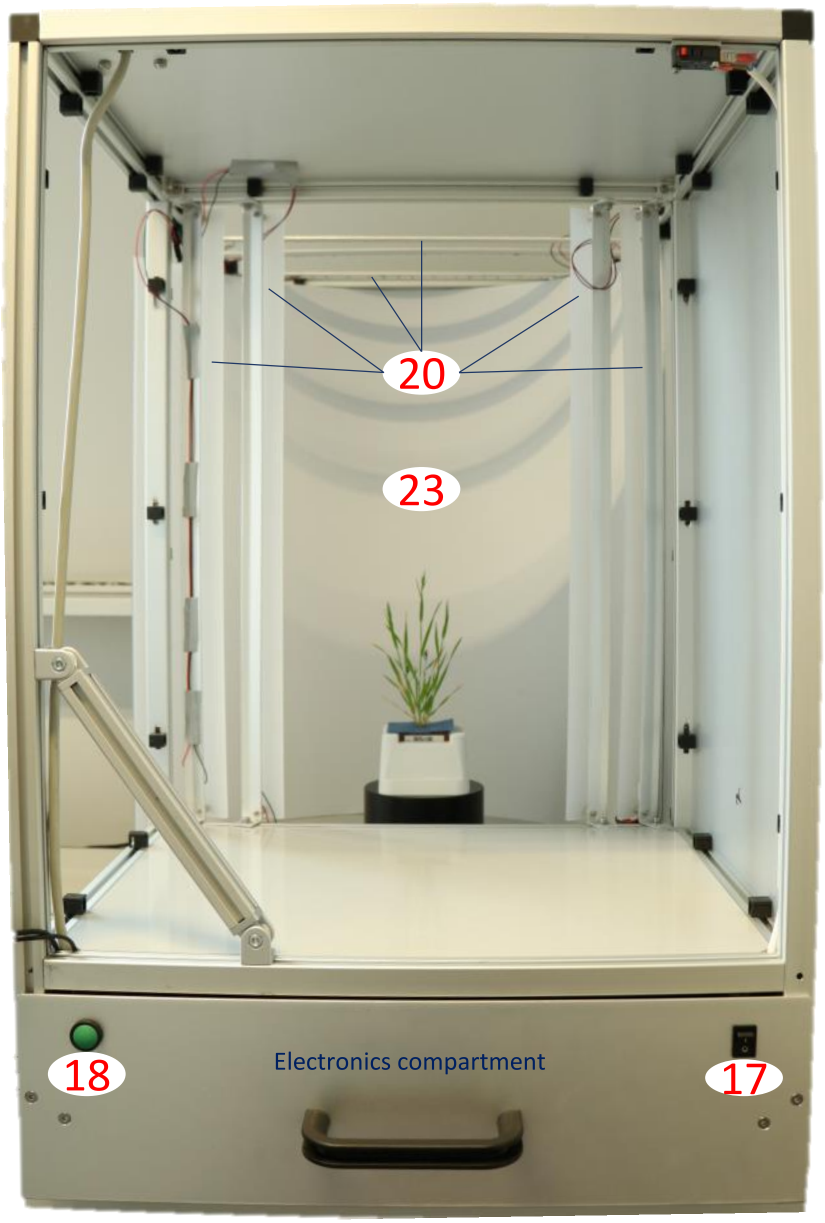

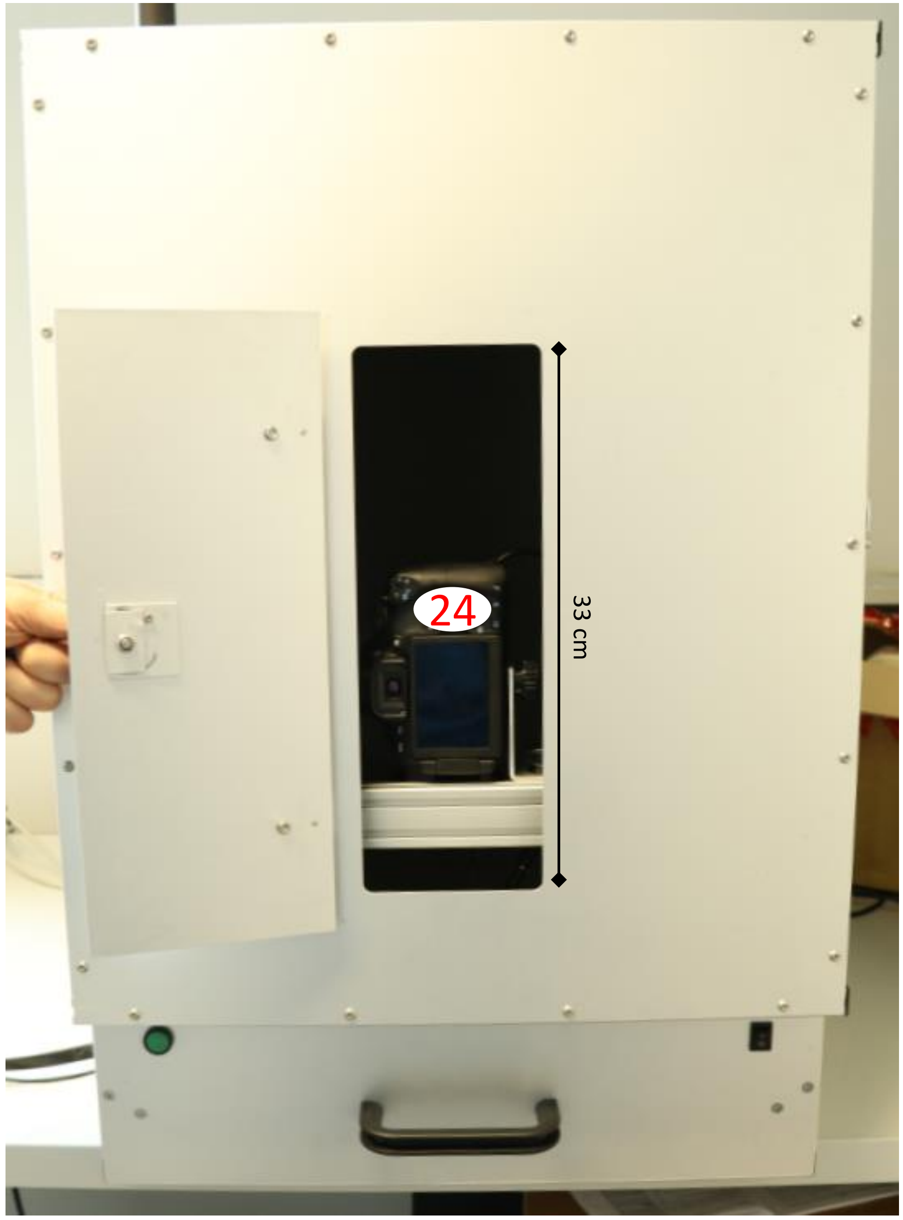

Phenobox door form inside + details of the camera height adjusting mechanism

Fixed position

push switch to adjust camera position up/down

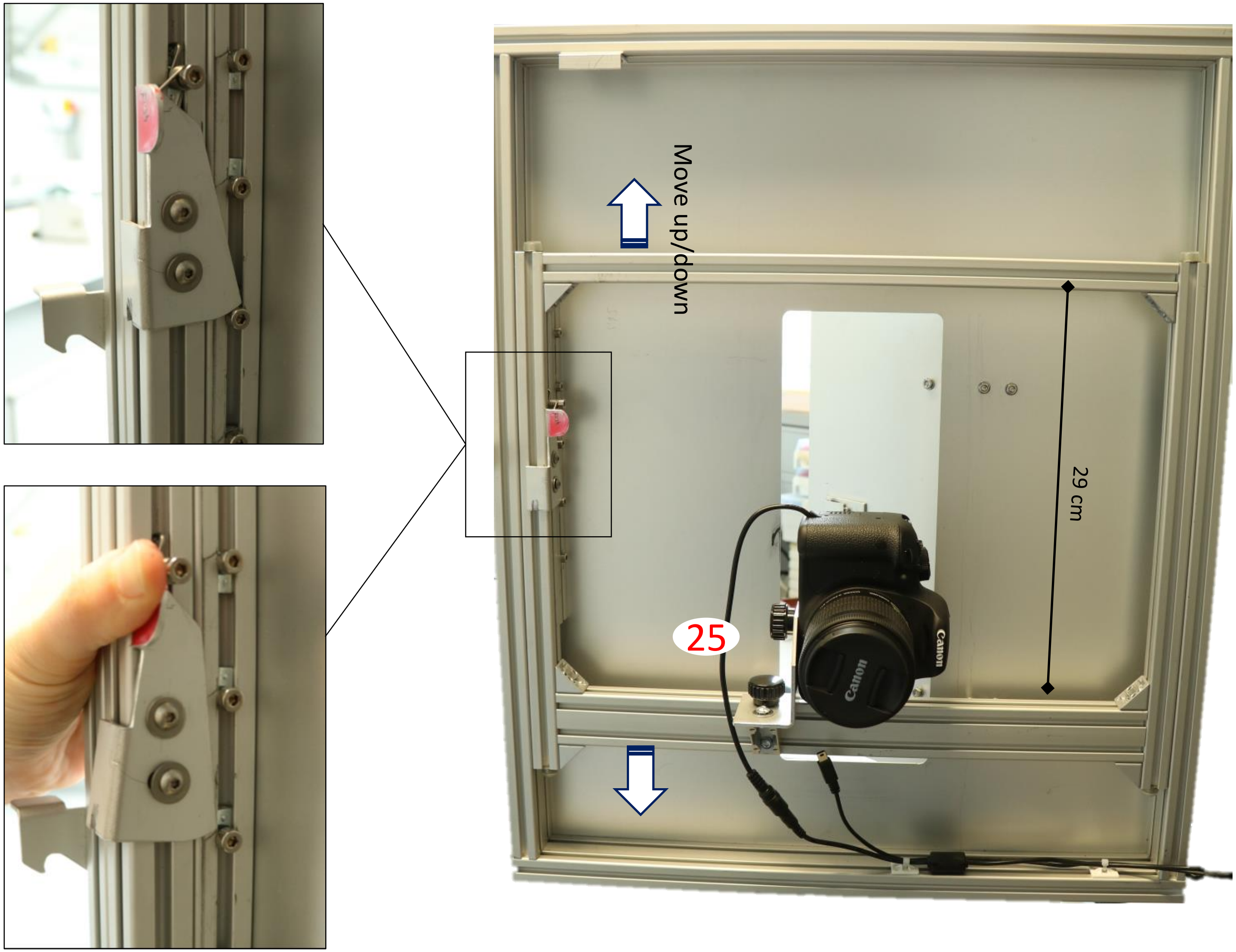

Pot adapator

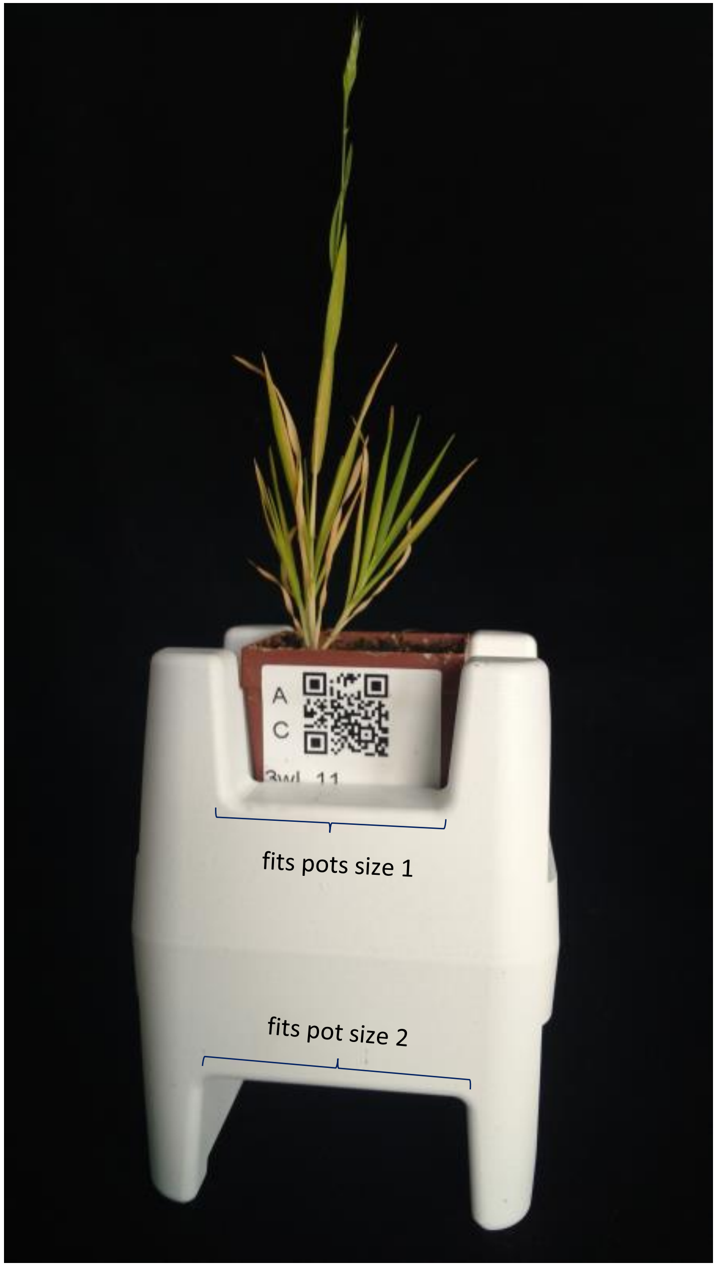

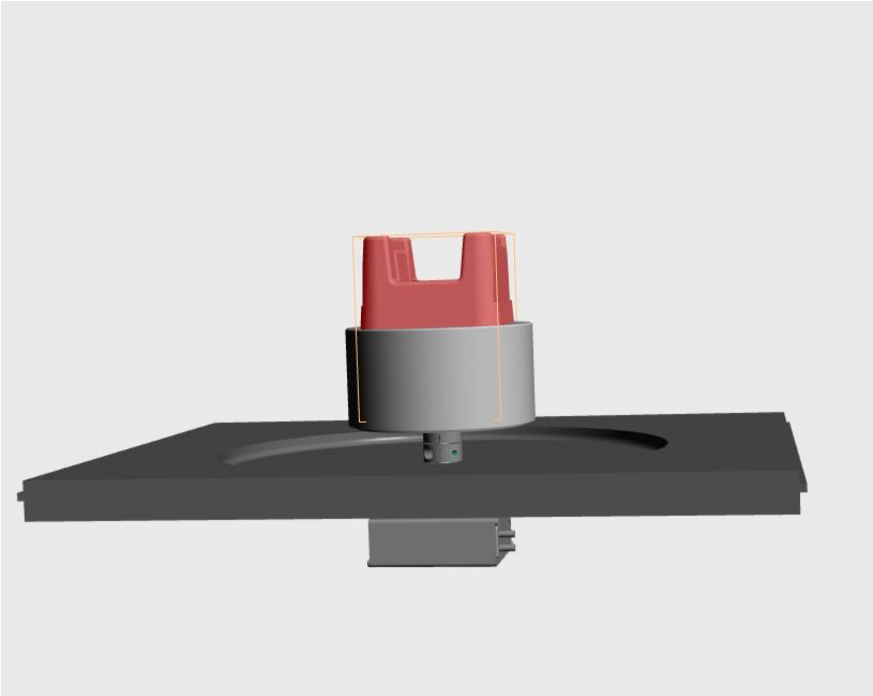

3D model of the pot adaptor (red) in context of the turntable/motor unit. The 3D model as an .pdf or .stp file can be downloaded as supplemental file.

Pot size1: #0388 Typ 1 Blumentopf tonrot 5,0x5,0x5,0 cm
Pot size2: #0554 Blumentopf Typ 2 tonrot 6,6x6,6x5,8 cm
Alpaco Lück GmbH & Co KG, Meinerzhagen, Germany

Component List:

	Component	Supplier	Quantity/Measure	Order Number*	approximate price	price x quantity
1	Transformer: TRACOPOWER 15W Embedded Switch Mode Power Supply SMPS, 3A, 5V dc	Tracopower, Baar, Switzerland	1	131965	55 €	55 €
2	Transformer: Logo! Power Switch Mode DIN Rail Panel Mount Power Supply, 24V dc to 24V dc, 4A	Siemens, Germany	1	734-2727	90 €	90 €
3	Main Controller: Raspberry Pi 3	Raspberry PI Foundation, Camebridge, UK	1	896-8660	33 €	33 €
4	Motor/Controller: SMC LER10K actuator	SMC Pneumatics, Korneuburg, Austria	1	LER10K-R36P1	760 €	760 €
5	Relay: Pololu 5V Relay Carrier	Pololu, Las Vegas, USA	1	Pololu item #: 2480	5 €	5 €
6	Relay: FINDER 34.51.7.024.0010	Finder, Almese, Italy	4	457-2846	5 €	20 €
7	Resistor (1/4W) 1k	[generic]	33	-	0.20 €	6.6 €
8	Resistor (1/4W) 4.7k	[generic]	3	-	0.20 €	0.6 €
9	Resistor (1/4W) 3.3k	[generic]	3	-	0.20 €	0.6 €
10	Capacitor 1u	[generic]	2	-	0.40 €	0.8 €
11	N-Channel Mosfet BS170	Fairchild Semiconductor, Phoenix, USA	14	671-4736	0.20 €	2.8 €
12	LED: KombiSIGN 70 Green LED Beacon, Steady Light Effect, 70mm Base, 24 V dc	Werma, Rietheim-Weilheim, Germany	1	2994424	32 €	32 €
13	LED: KombiSIGN 70 Red LED Beacon, Steady Light Effect, 70mm Base, 24 V dc	Werma, Rietheim-Weilheim, Germany	1	2994402	32 €	32 €
14	LED: KombiSIGN 70 Yellow LED Beacon, Steady Light Effect, 70mm Base, 24 V dc	Werma, Rietheim-Weilheim, Germany	1	2994418	32 €	32 €
15	LED: KombiSIGN 70 Blue LED Beacon, Steady Light Effect, 70mm Base, 24 V dc	Werma, Rietheim-Weilheim, Germany	1	3659942	32 €	32 €
16	Support Tube and Base for use with KombiSIGN 70/71	Werma, Rietheim-Weilheim, Germany	1	3147332	15 €	15 €
17	KombiSIGN 70 Termination Unit, 70mm Base, 24 V dc, 230 V ac	Werma, Rietheim-Weilheim, Germany	1	3147326	18 €	18 €
18	Rocker switch (on/off switch)	RS-Components, Mörfelden-Walldorf, Germany	1	2828248	3 €	3 €
19	Push button	RS-Components, Mörfelden-Walldorf, Germany	1	321256	11 €	11 €
20	Axial flow fan (for electronics compartment)	RS-Components, Mörfelden-Walldorf, Germany	1	3811263	31 €	31 €
21	LED light source: BC Series High CRI LED 2835 Hybrid Color Temperature Ribbon, 120 LED/m	Yuji LED, Beijing, China	1 (5m)	Yuji #: 2835	90 €	90 €
22	Construction profile type 5	[generic]	different sizes		400 €	400 €
23		[generic]	different sizes		120 €	120 €
24	Rounded back wall: Water Resistant Backlit 130µ	Canon, Ota, Japan	66 x 80cm	Canon #: 9172A	90 €	90 €
25	Camera: Canon EOS 700D with EF-S 18-55mm IS STM objective and power supply	Canon, Ota, Japan	1	Canon #: EOS 700D	500 €	500 €
26	Polaroid camer power supply for Canon EOS T5i, T4i, T3i, T2i Digital Cameras (Canon ACK-E8 / ACKE8 Replacement)	Polaroid, Minnetonke, USA	1	-	20 €	20 €
27	Label printer, 300 dpi	Labelident, Schweinfurth, Germany	1	Labelident #LD BP730	320 €	320 €
28	Thermotransfer color band Harz Kurz K504, 2" width	Labelident, Schweinfurth, Germany	1	nt #FTA-LH050-300BK	10 €	10 €
29	Custom size polyester labels, 40,0 mm x 30,0 mm	Labelident, Schweinfurth, Germany	8	#ERT-SOAF-AX-10165	30 €	30 €
30	Plastic material for Base of teh PhenoBox and Pot adaptor	[generic]	-	-	50 €	50 €
31	Various connecting materials and fastening materials	[generic]	-	-	150 €	150 €
	* if not otherwise stated, Order Numbers are RS-Components IDs http://www.rs-online.com/				Sum	2,960 €
