# Supplementary material for: The ‘PhenoBox’, a flexible, automated, open‐source plant phenotyping solution
Source: New Phytol. 2018 Apr 5;219(2):808–23. doi: 10.1111/nph.15129 (PMC6485332; doi:10.1111/nph.15129)
Supplement: Supplementary file 12 — Notes S5 PhenoBox system user guide. [file NPH-219-808-s012.pdf]

The "Phenobox", a flexible, automated, open-source plant phenotyping solution

Angelika Czedik-Eysenberg, Sebastian Seitner, Ulrich Güldener, Stefanie Koemeda, Jakub Jez, Martin Colombini, Armin Djamei

Accepted 22.2.2018

The PhenoBox/PhenoPipe system - User guide

Table of Contents

Example application 2
Project Creation and Plant Imaging 2
Project Creation 2
Print labels 5
Imaging plants with the PhenoBox 6
Analysis and Postprocessing 7
Create an IAP image analysis pipeline 7
Upload and select an IAP pipeline within the PhenoPipe interface 11
Create a postprocessing Stack 11
Start IAP analysis and postprocessing for an imaging time point 13
Task Status 14
View and download results after analysis 15
Snapshot exclusion for postprocessing 17
Running a Postprocess on existing IAP results 18

Example application

In this user guide exemplify the use of the Phenobox/PhenoPipe system by describing the imaging and processing of an experiment in which the phenotypic response of *Nicotiana benthamiana* plants treated with 400mM NaCl solution was compared to controls. The results of this experiments are described in our publication "The "Phenobox", a flexible, automated, open-source plant phenotyping solution" by Czedik-Eysenberg, Seitner et al., in the context of which this user guide is provided.
This experiment is entered as project "BenthaminaNaCl2" into the PhenoPipe.

Project Creation and Plant Imaging

Project Creation

Click "Create Project" in the navigation bar on the right in the PhenoPipe interface. This then opens a page where you are asked to enter project information. Please supply a project name and specify the start date of the experiment. It usually makes sense to choose the planting date or the date when treatment starts. If desired, two dates can be entered (i.e. planting date and the date when treatment started, if this information is required in further evaluation steps). This is achieved by clicking "use another date for analysis timeline" and selecting a second date. Entering a project description is optional but can be very helpful for documentation purposes.

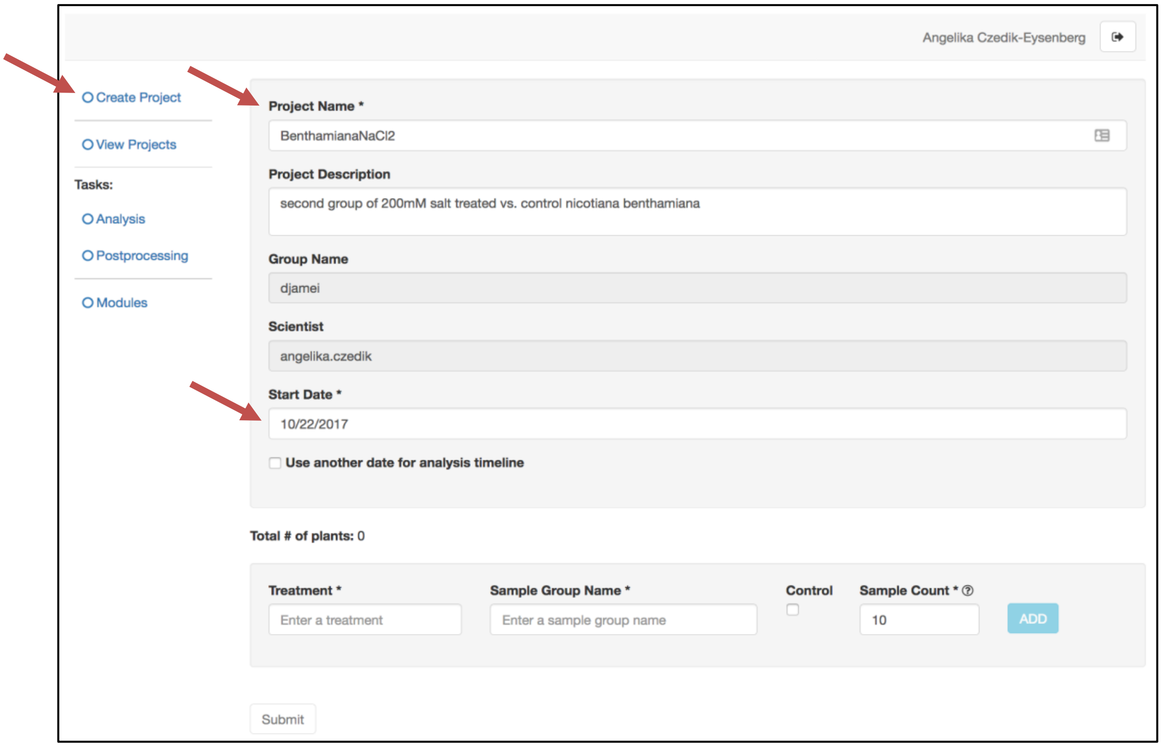

Entering sample groups

In the next step, sample groups are defined. These groups can then be plotted and compared in the postprocessing steps.

The field "Treatment" contains the treatment name that will later be displayed in the postprocessing results, while "Sample Group Name" specifies the name that will be displayed on the pot labels. The sample group name cannot be longer than 16 characters due to the limited space on the labels. "Sample count" specifies how many plants of this type will be used in the experiment. It is no problem if there are more plants in the group than finally imaged. The tick box "control" indicates if a sample group should be internally specified as control. This is required for certain postprocessing applications, for example if several treatments should each be compared to one shared baseline group.

In our example we enter 2 groups, the control plants and the NaCl treated. The images show the addition of the control group.

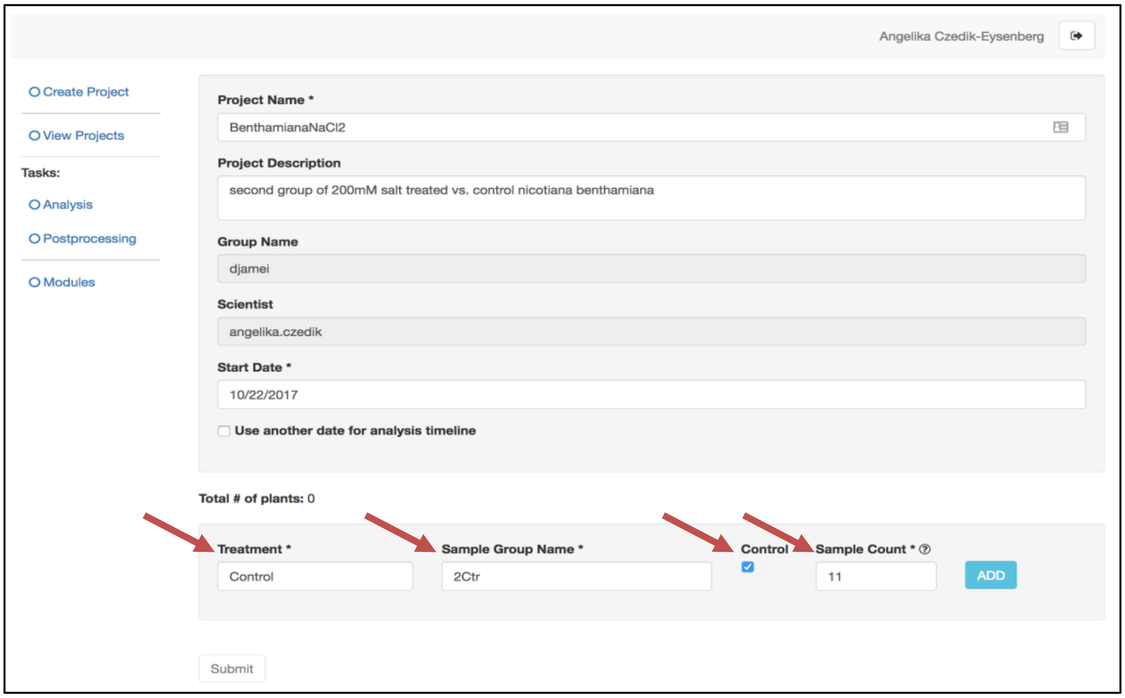

Once overall group information has been entered, click the "Add"-button. This now creates the group, which is visible at the bottom of the page and can be expanded by clicking the small arrow on the right.

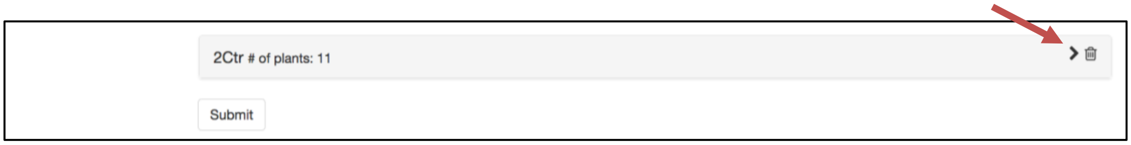

When a sample group is expanded, it is possible to modify the previously entered information, as well as add additional sample group metadata. These metadata are preserved throughout the IAP segmentation and can thus be used in post processing modules. It is also possible to add text to the pot labels of individual plants. This is however again limited by the space on the labels.

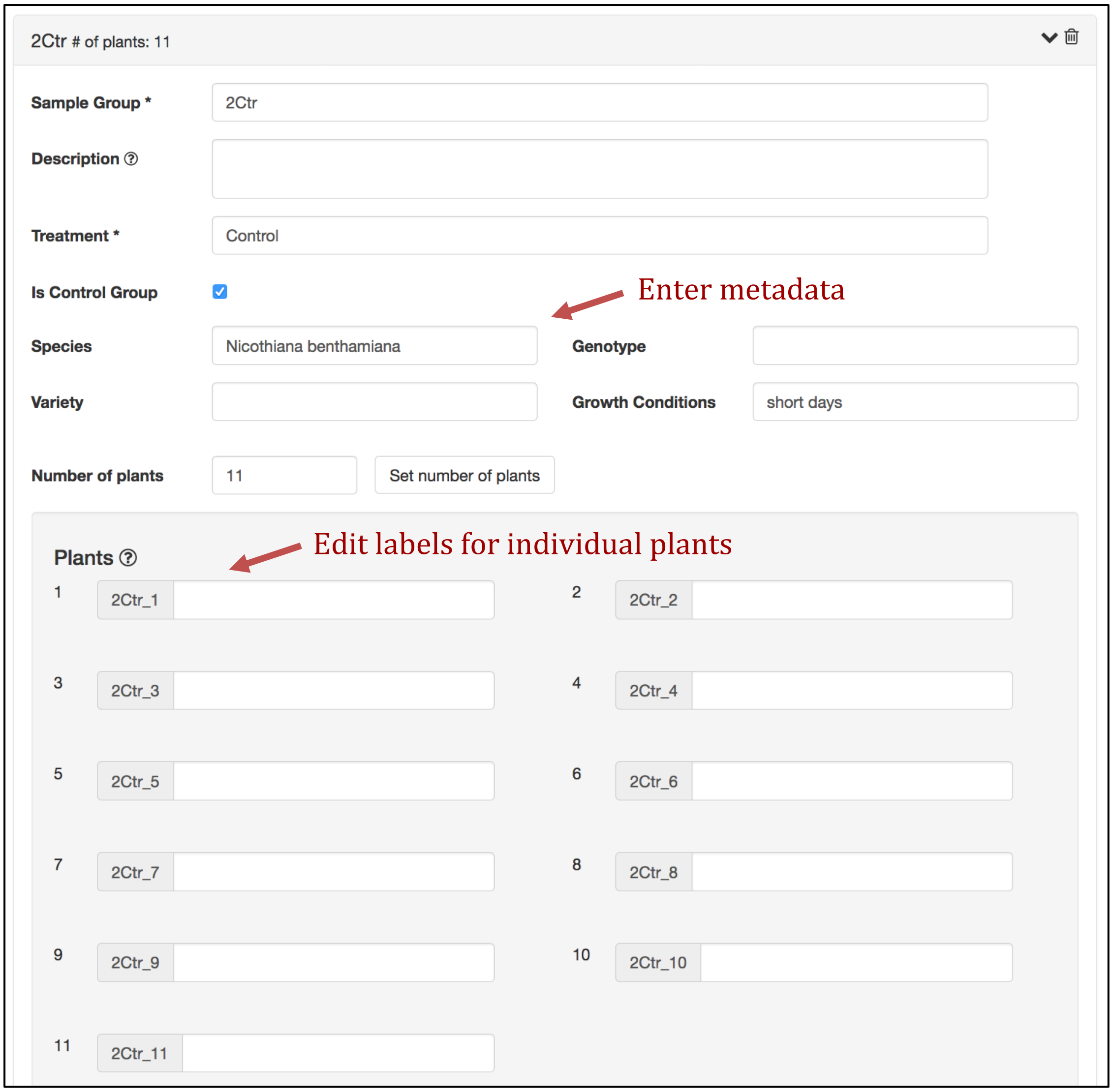

When information for all sample groups has been entered, click "Submit" to save the project. After clicking "Submit", you get to a screen summarizing the project information, which also allows you to go back and edit information. You can leave this screen and the whole interface at this step and later get back to this screen by clicking "View Projects" in the navigation bar in the left side.

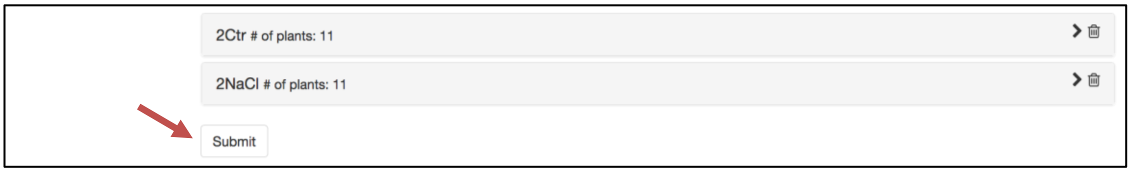

Print labels

To use the PhenoBox, it is necessary to print QR-code containing labels for all plants and stick them onto the pots, so that they are clearly visible once the pots are put into the PhenoBox pot adaptor of the box.

To do so navigate to your project (see previous page). Make sure that all sample group information is correct and then proceed to click "Print all Labels". You will be prompted to confirm your action, to avoid accidentally printing a lot of unnecessary labels.

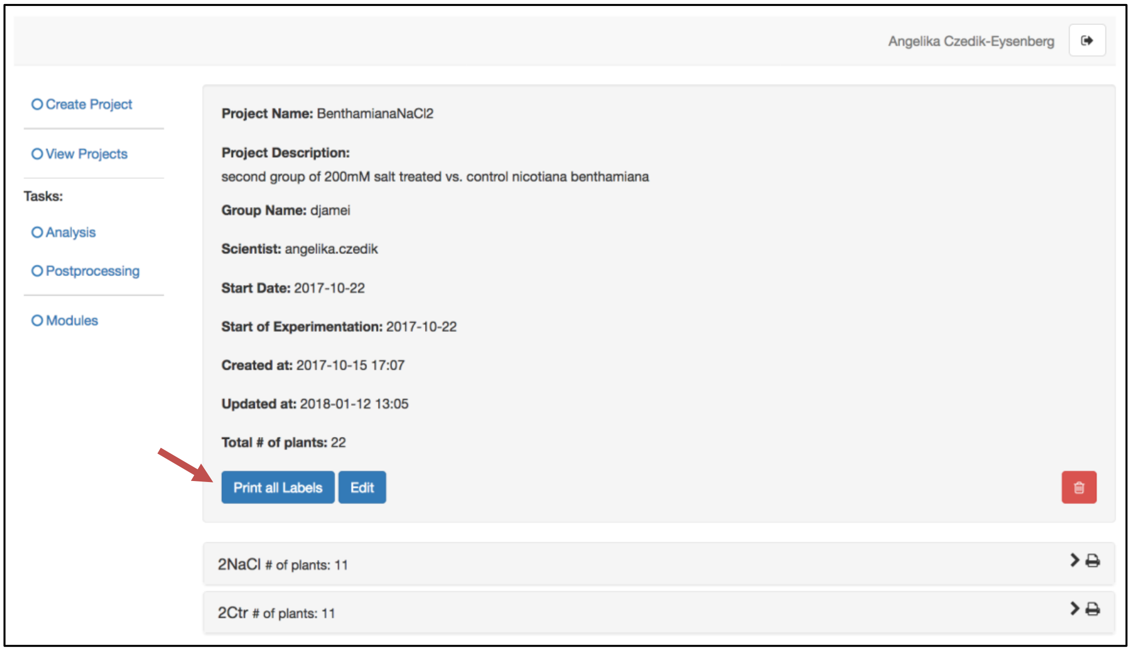

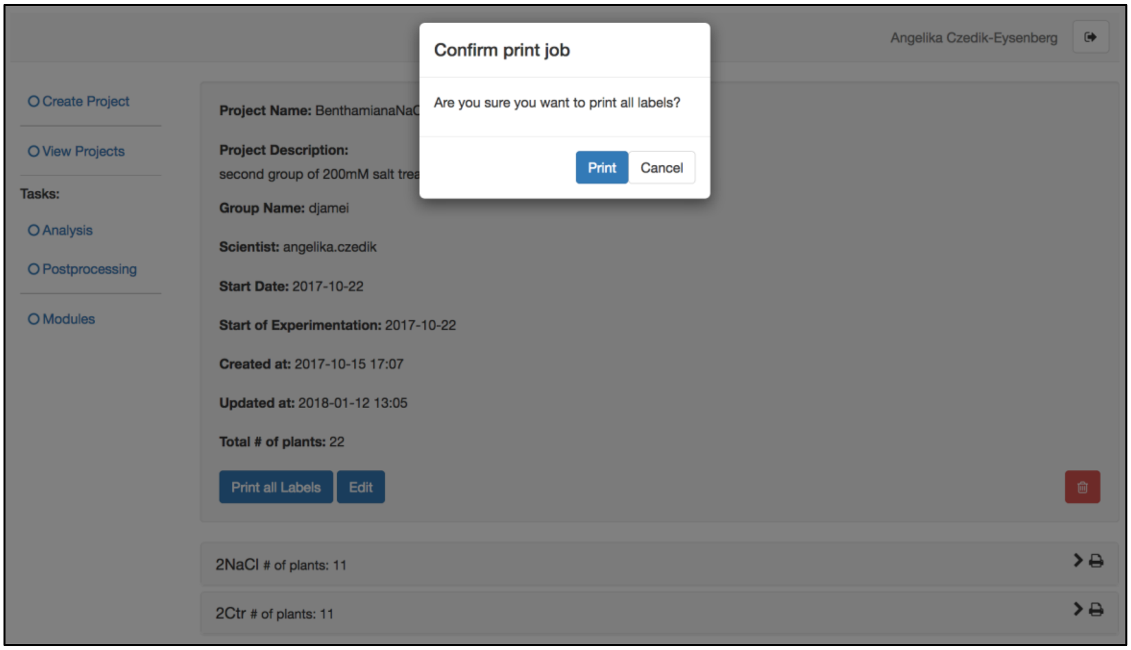

Imaging plants with the PhenoBox

Now that you printed all labels and attached them to your pots you are ready to take pictures using the PhenoBox. After starting up your PhenoBox, put a plant into the box, close the door and press the start button. The box will subsequently take a first picture, decode the QR code to identify the plant, and proceed to take pictures from different angles if the recognition was successful. Errors are indicated by the signal lamps on the PhenoBox and the status screen/error log. The green status lamp indicates that imaging of a plant is complete and the next plant can be entered. While you continue to image plants, the previously taken images are automatically converted and uploaded to the server.

After imaging your plants go back to the web interface and navigate to your project. You will see that the system created a timestamp to which it assigned the new images. If you want to retake an image, you first have to delete the snapshot by expanding the according group and delete the snapshot of the specific plant. After that you can image the plant again - otherwise the Phenobox will refuse to take images because you have already imaged this plant.

As you now have imaged all plants, you must finalize the timestamp to process the data. Before clicking on finalize, you should check if you have not forgotten any plants or if you want/must retake any images, since as of now finalization of a timestamp is not reversible. Currently, you have to download the images to inspect them. (We plan to implement image display in the browser in the future.) After finalizing a time point, the system will create a new timestamp if you image the same plants again.

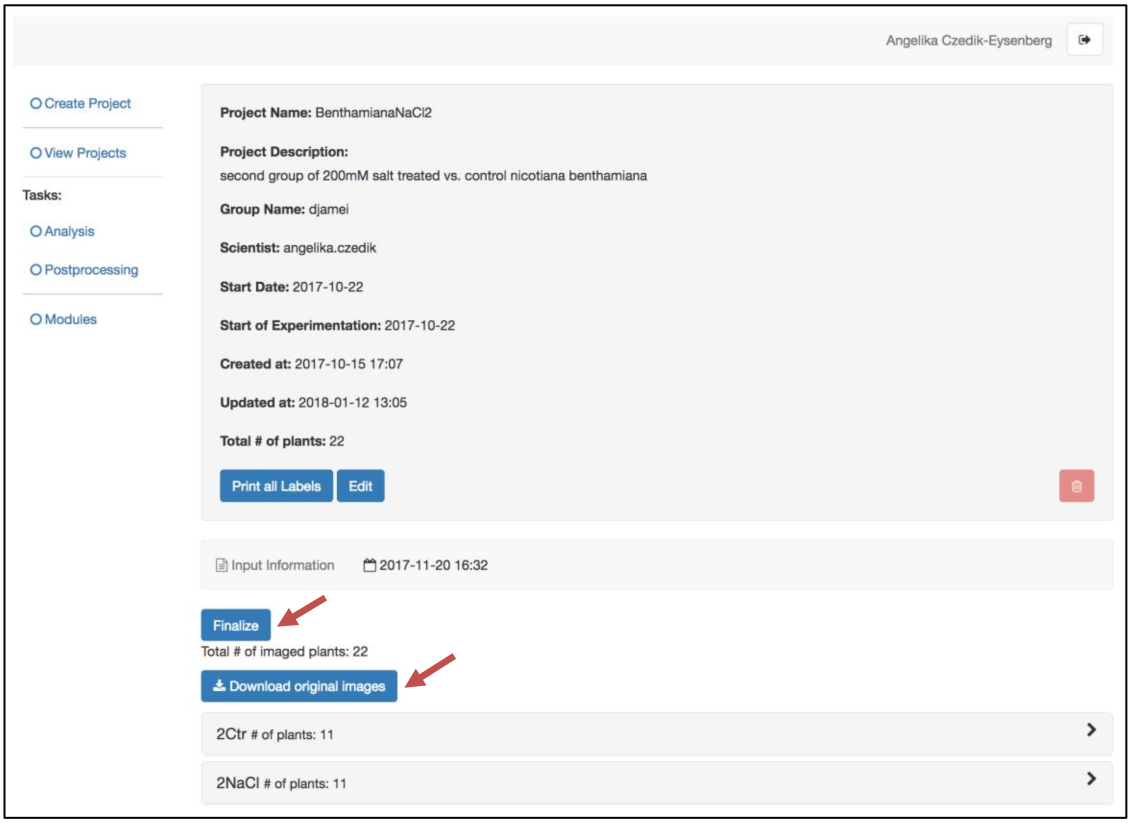

Analysis and Postprocessing

Once you finalized your timestamp you can start an analysis on the images. There is the option to exclude certain plants from postprocessing, which is described further down. To be able to analyze and postprocess your data you require an IAP pipeline for image analysis and a Postprocessing stack for further computations.

Create an IAP image analysis pipeline

For each type of imaging project (i.e. different species or plant sizes) you have to once create an IAP image analysis pipeline by running IAP manually on your client computer. IAP v2.1.0 can be downloaded from https://openimageanalysisgroup.github.io/IAP/ and detailed documentation can be found there. Here, a short summary of the necessary steps is given.

First create an IAP dataset with your images and load the dataset. Select the file import folder and within your project. Click "Analysis" and "Select Template / Remove Setting" to select an existing pipeline as basis for your new pipeline. Here we select the "Barley Analysis" pipeline pre-supplied with IAP.

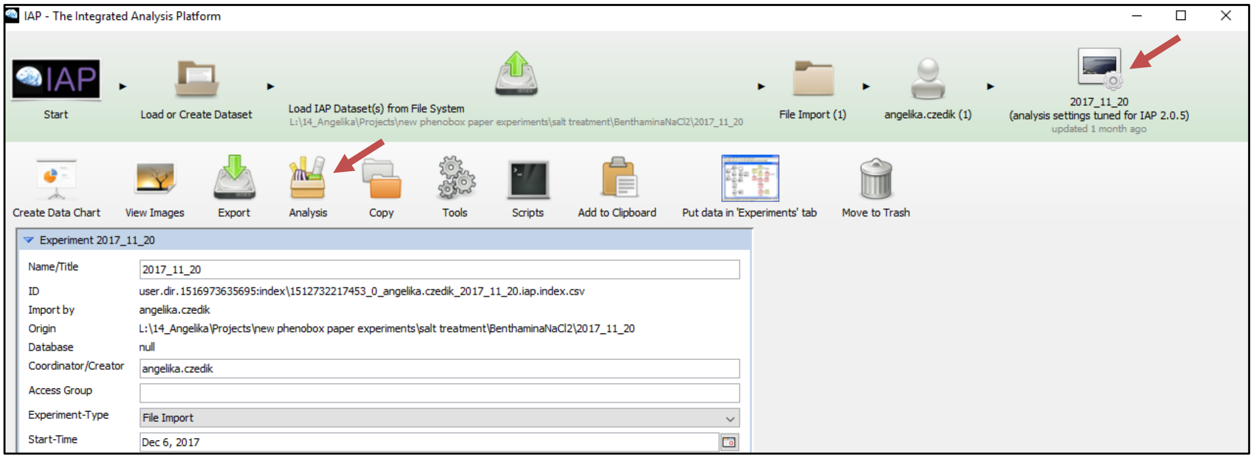

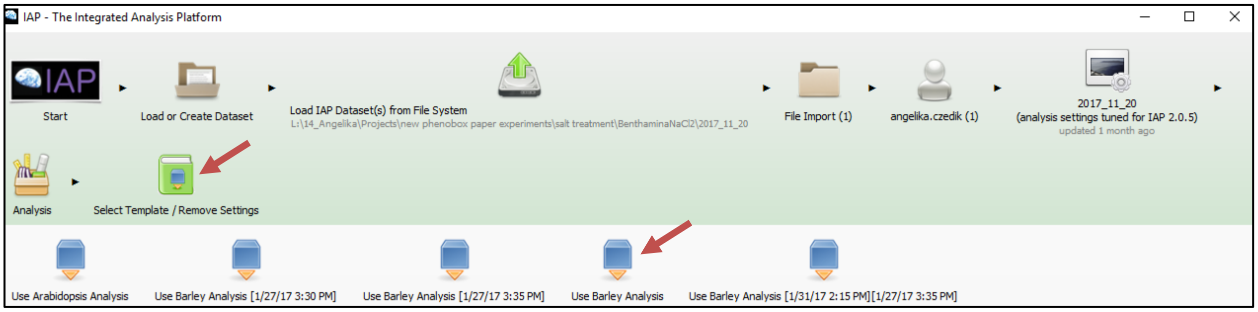

Go back to your project and click "View Images" and select "vis.side". You see your images displayed. If you click on one image, you get a menu where you select your specified pipeline (here "Barley Analysis (Image + Reference)"), to start analysis of this image with the pipeline.

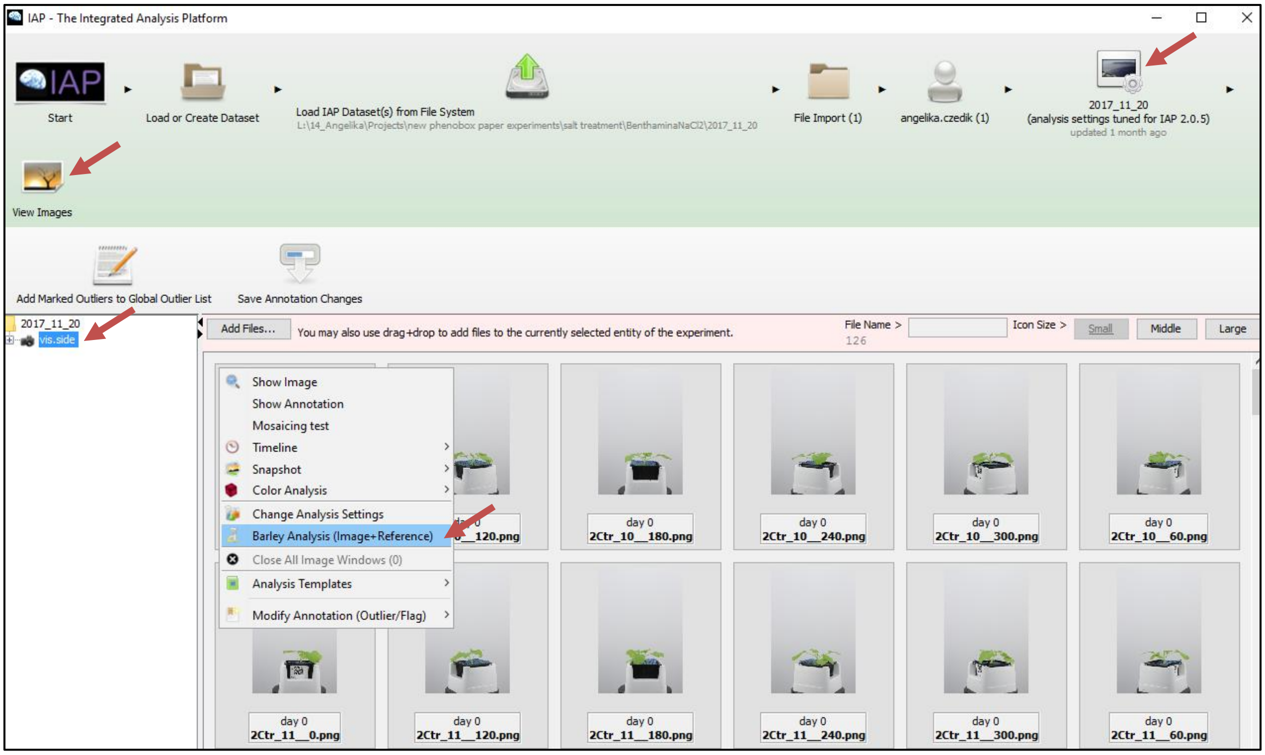

IAP - The Integrated Analysis Platform
Start
Load or Create Dataset
Load IAP Dataset(s) from File System
L:\14_Angelika\Projects\new phenobox paper experiments\salt treatment\BenthaminaNaCl2\2017_11_20
File Import (1)
angelika.czedik (1)
2017_11_20
(analysis settings tuned for IAP 2.0.5)
updated 1 month ago
View Images
Add Marked Outliers to Global Outlier List
Save Annotation Changes
2017_11_20
vis.side
Add Files...
You may also use drag+drop to add files to the currently selected entity of the experiment.
File Name >
126
Icon Size >
Small
Middle
Large
Show Image
Show Annotation
Mosaicing test
Timeline
Snapshot
Color Analysis
Change Analysis Settings
Barley Analysis (Image+Reference)
Close All Image Windows (0)
Analysis Templates
Modify Annotation (Outlier/Flag)
day 0
2Ctr_10__180.png
day 0
2Ctr_10__240.png
day 0
2Ctr_10__300.png
day 0
2Ctr_10__60.png
day 0
2Ctr_11__0.png
day 0
2Ctr_11__120.png
day 0
2Ctr_11__180.png
day 0
2Ctr_11__240.png
day 0
2Ctr_11__300.png
day 0
2Ctr_11__60.png

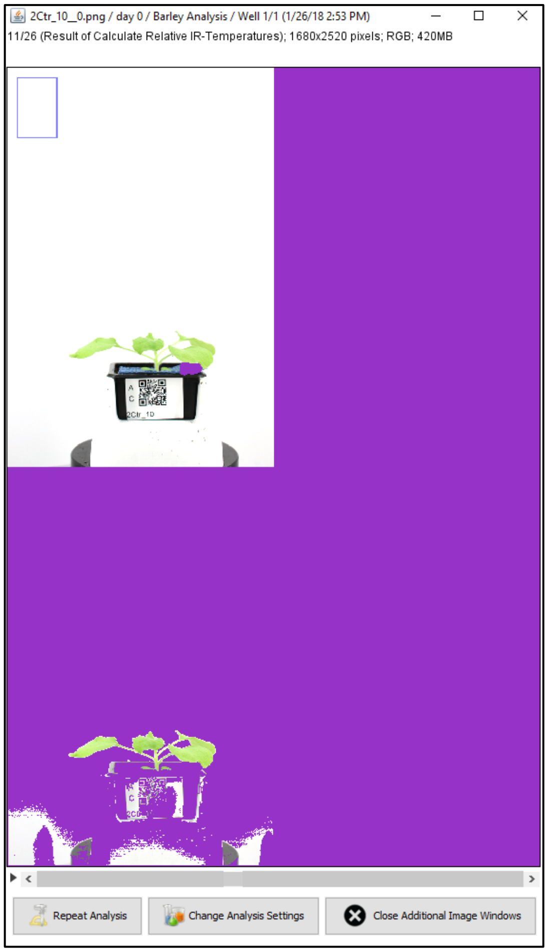

IAP calls ImageJ and a window where you can look at all steps of the analysis pops up.

You can scroll through the steps and click “Change Analysis Setting” to adjust settings for a given step, and then “Repeat Analysis” to evaluate the changes.

In our experience, it is especially critical to set the background color correctly, compare different background removal algorithms, and fine tune color filtering limits to achieve good plant segmentation.

The optimized pipeline should be tested on several representative plants and be adjusted accordingly.

To export the pipeline from IAP, go back to your project and select “Analysis” → “Export / Modify Settings” → “Description” to set the pipeline name and description.

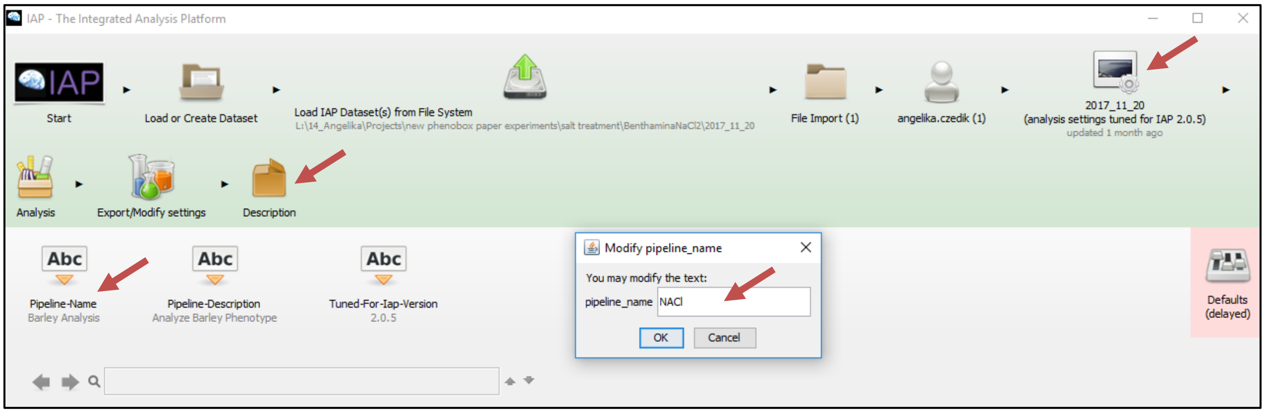

Then go back to “Analysis” → “Export / Modify Settings” and select “Export”. A window pops up, displaying the save location of your pipeline file.

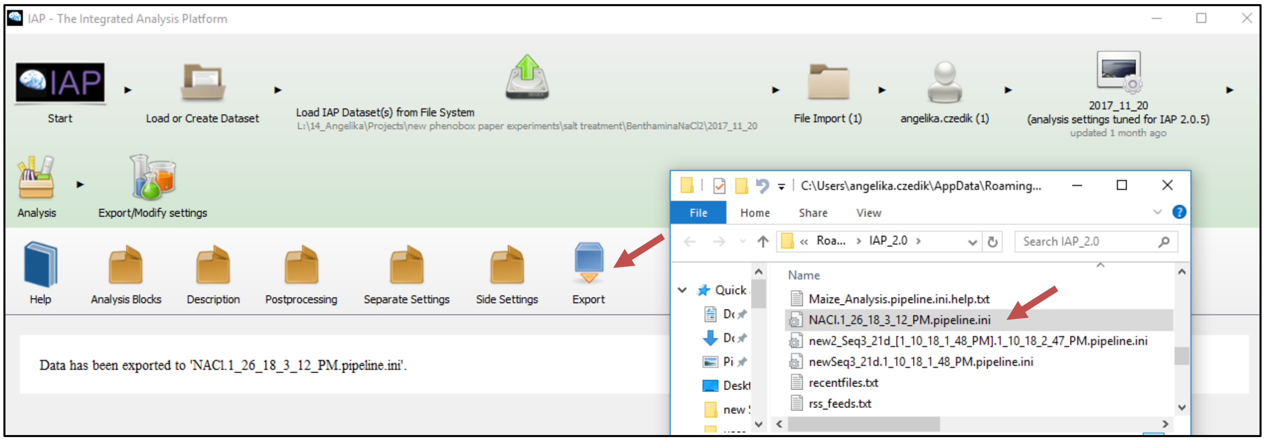

Upload and select an IAP pipeline within the PhenoPipe interface

If you go to the modules section of the PhenoPipe web interface you will first be presented with a list of available IAP Pipelines. Click on the 'Upload Pipeline' button to upload a new pipeline.

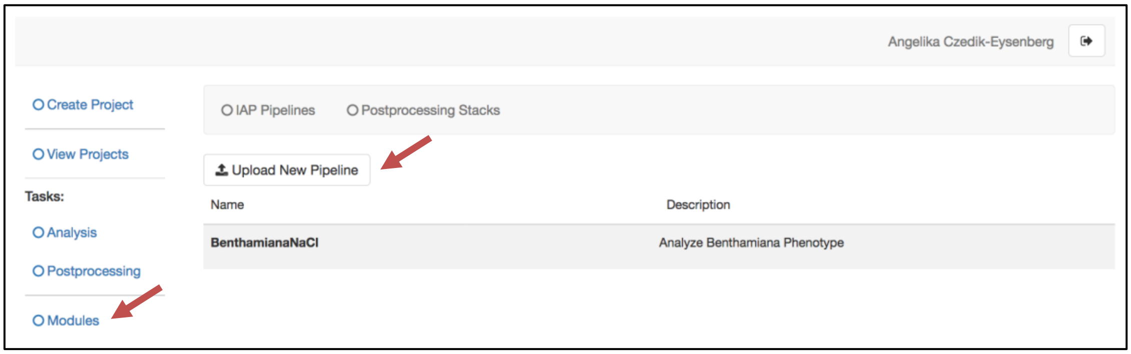

After you have selected the pipeline, it will show you the filename beside the button. The file name does not matter to the system, as each IAP pipeline file contains a description block which contains the name and a description of the pipeline. The PhenoPipe system will use these two values instead of the filename. This may for example look like this:

```
[DESCRIPTION]
pipeline_name = BenthamianaNaCl
pipeline_description = Analyze Benthamiana Phenotype
```

After you click on upload you will get a little message to indicate whether the upload was successful or not.

Create a postprocessing Stack

To analyze the results generated by IAP, a postprocessing stack is needed.

A postprocessing stack is basically a collection of R scripts which are executed sequentially and can pass data on to the next script. Before you create a stack you may want to familiarize yourself with the concept of Postprocessing stacks and the according rules by reading about it in the PhenoBox/PhenoPipe wiki. Enter a name and a description of what your stack will do so that you can easily choose the appropriate stack when analyzing your experiments. After you have done that, add the individual scripts you want to run in your stack.

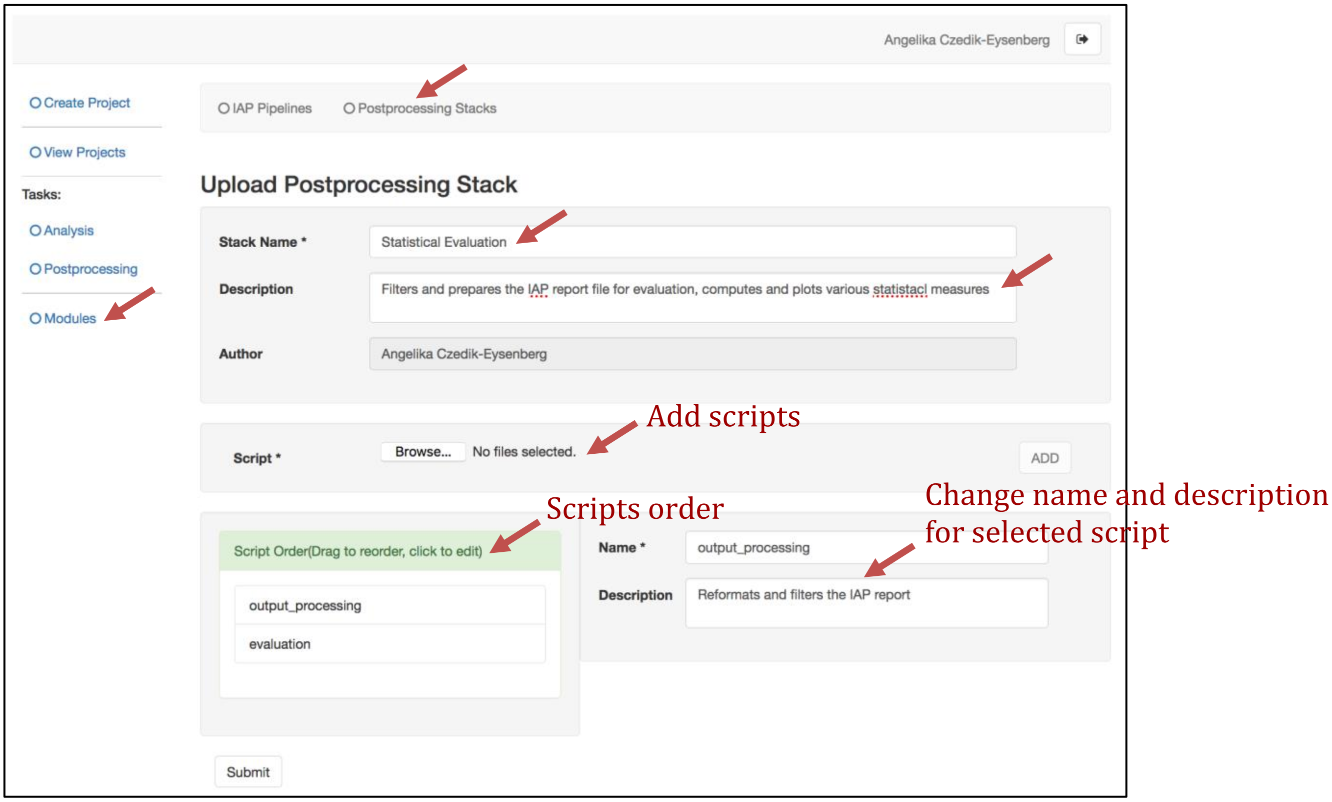

When adding scripts you can select multiple R-scripts (".r" files) at once so you don't have to add them separately. You can reorder the scripts by dragging them up or down inside the box. The order you see here is the order in which the scripts will be executed. When you click on an entry in the box you will see two fields on the right side in which you can enter a name and a description for the individual scripts. Per default, the file name is used as script name and a description is optional. In our example case we add a script to filter the IAP report and reformat it to create the desired structure for our evaluation script which will operate on the filtered and formatted data ("output_processing").

If you have added and ordered all scripts, you can press submit to upload the new stack to the server. You will get a notification on the bottom that indicates success or failure.

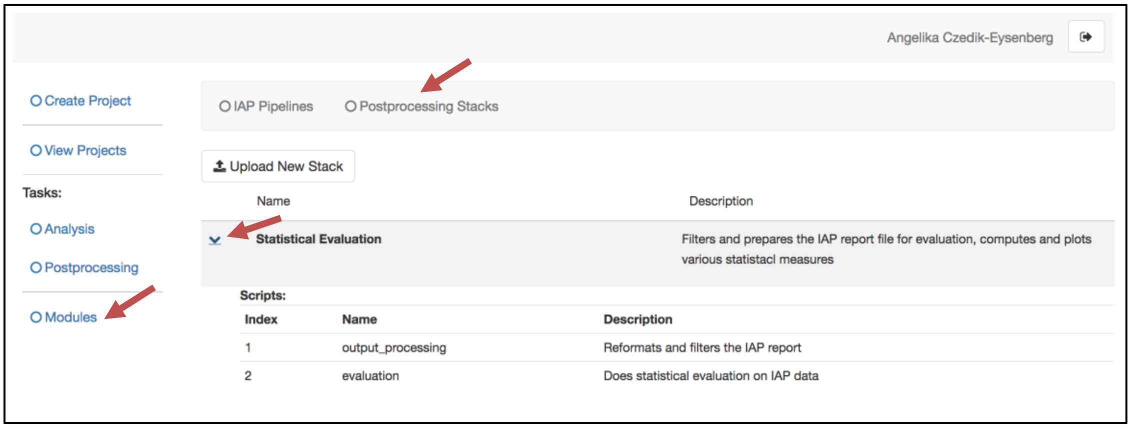

After you have uploaded a postprocessing stack you can expand its details by clicking on the small arrow in the postprocessing stack list in the modules section.

Start IAP analysis and postprocessing for an imaging time point

To analyze a timestamp, click on 'View Projects' and select the desired experiment. In the detail view, click on the timestamp you want to analyze. Now, if you have completed the timestamp already, you will see the 'Analyse' button at the top of the timestamp information. When you click on it, you will be redirected to a page where you can select and IAP pipeline and postprocessing stack.

Select the IAP pipeline uploaded before. Once you have done that, you will see the details of the pipeline right below to make sure you selected the correct one.

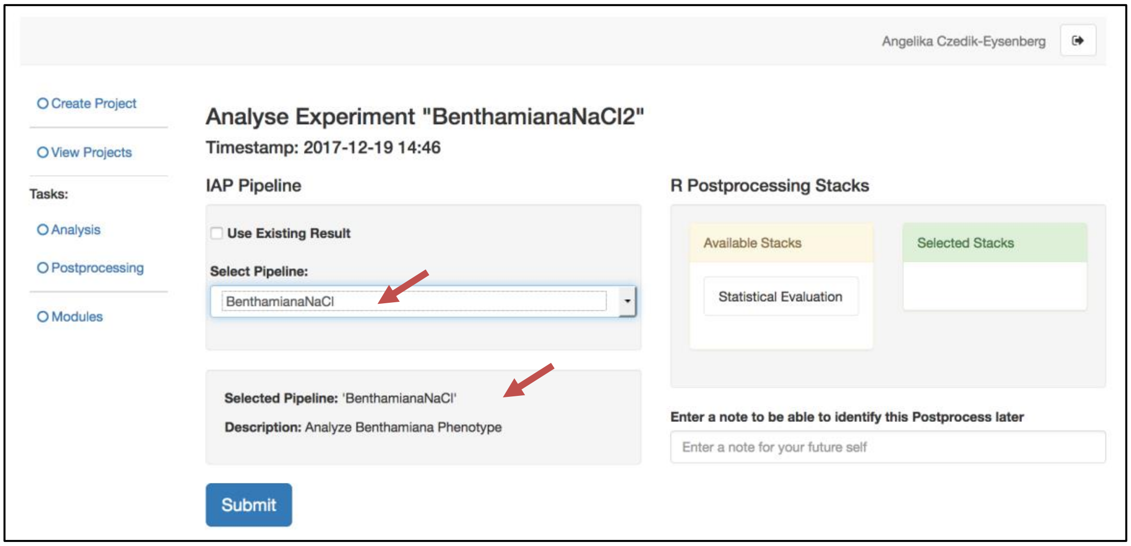

Now, you can optionally select the postprocessing stack you want. If you do this at this point, the postprocessing will run directly after the image analysis is completed saving you some time and clicks. There is, however, also the option to invoke the postprocessing manually after an analysis is finished. Thus, you always have the option to run another postprocessing stack on your results later.

To select a postprocessing stack, you just drag it into the box labeled 'Selected Stacks'. If you add more than one stack, the order in this box does not matter because each stack is executed independently. If you want to see more information about a stack just click on it and you will be presented with its details.

Postprocess Note

There is an additional textbox below the stack selection in which you can enter a note to identify this postprocessing run later on. This is helpful in situations such as, if you want to run the same postprocessing stack multiple times, while excluding different plants from the processing each time. Without a note can be hard to identify the results later on.

After you have selected everything, you can press submit to start the tasks accordingly. You will get a notification on the bottom indicating success or failure of task submission.

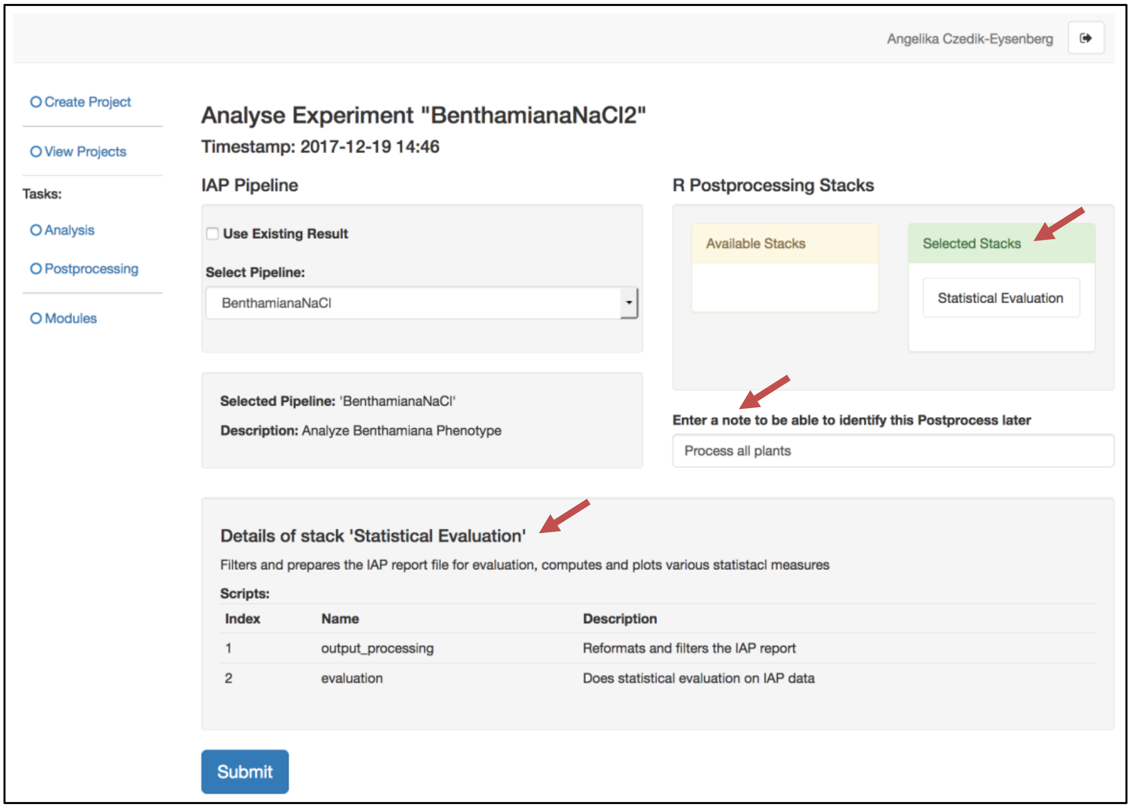

Task Status

After submitting an image analysis task and/or postprocessing task, you can watch the progress of your tasks by clicking on one of the links in the 'Tasks' section of the navigation sidebar.

Analysis Status

Pending: This means that the task is currently enqueued and will be started as soon as possible.

Running: Data are currently processed.
E.g. in the example, images are currently imported into IAP.

To get more detailed messages just click on the button under 'View Log'.

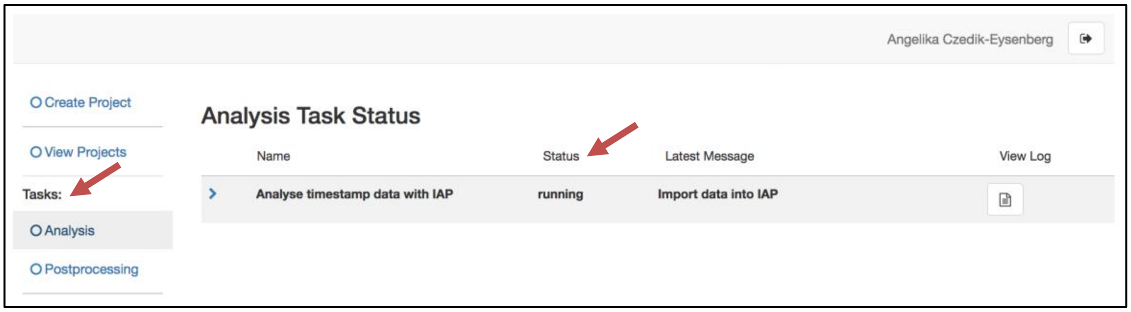

View and download results after analysis

After successful analysis, the timestamp view (View Projects → select your project and within the timestamp) looks as depicted below. You can see all pipelines that were used to analyze the images of this time stamp, and you can start an additional postprocess run right from this view.

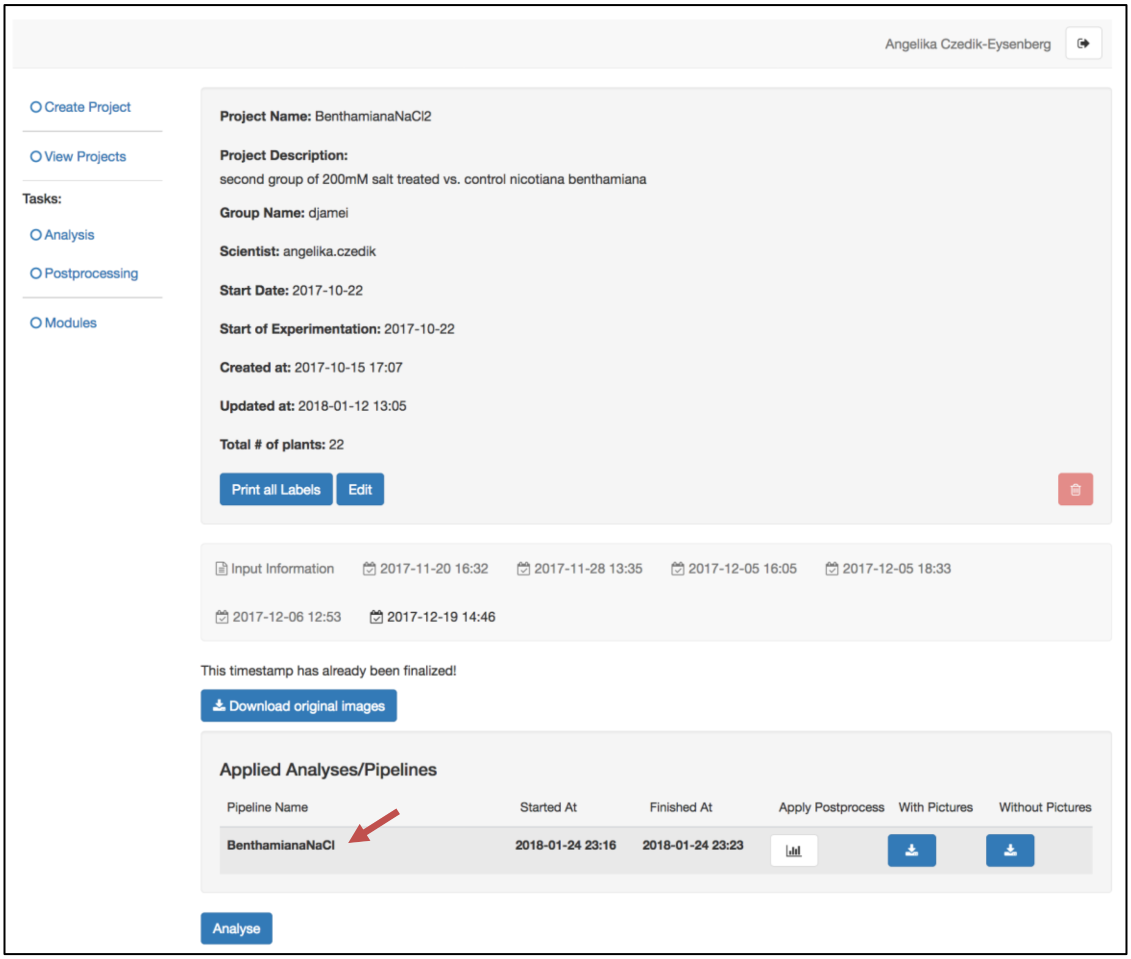

If you click on the entry, you will open a more detailed view where you can also see information about the applied postprocessing. When you click on any of the download buttons you will receive a zip archive with the results. For large projects, it may take a few moments before the download starts. The option to view the results right in the browser is not yet implemented, but we plan to implement it in the future.

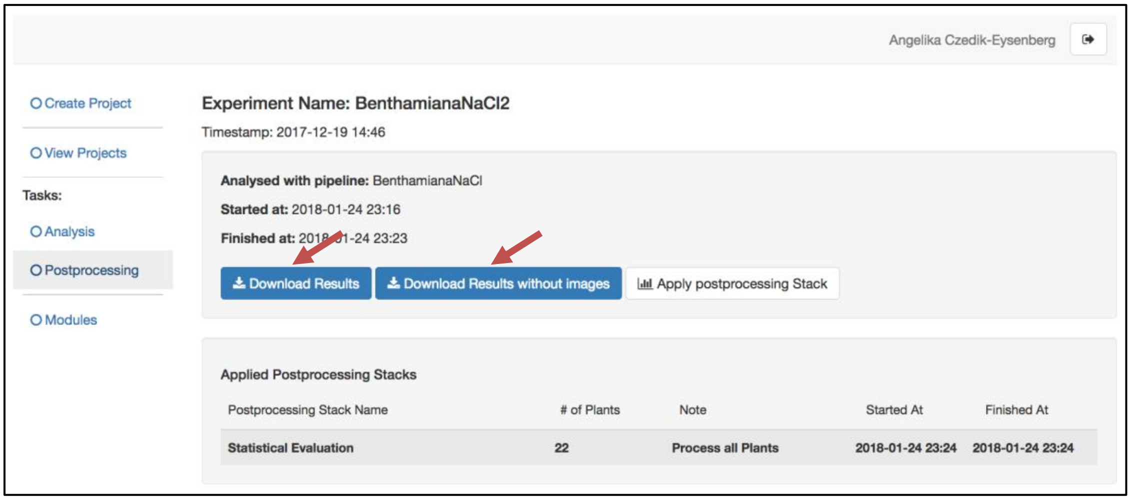
Angelika Czedik-Eysenberg
Create Project
View Projects
Tasks:
Analysis
Postprocessing
Modules
Experiment Name: BenthamianaNaCl2
Timestamp: 2017-12-19 14:46
Analysed with pipeline: BenthamianaNaCl
Started at: 2018-01-24 23:16
Finished at: 2018-01-24 23:23
Download Results
Download Results without images
Apply postprocessing Stack
Applied Postprocessing Stacks
Postprocessing Stack Name
# of Plants
Note
Started At
Finished At
Statistical Evaluation
22
Process all Plants
2018-01-24 23:24
2018-01-24 23:24

Snapshot exclusion for postprocessing

IAP analysis is always run on all available pictures for a given timestamp, but you have the option to exclude certain plants from postprocessing. You may run a postprocessing stack multiple times and exclude different plants each time. To exclude plants, navigate to the timestamp of the project and expand the sample group you want to exclude plants from. You will see a list of available snapshots inside every sample group. Those are all plants which have been imaged for the selected timestamp. In the example below, we exclude the last two control plants.

To exclude a plant simply click on the blue slider on its right side. Excluded snapshots will be marked so you can identify them easily. This selection will be remembered and applied to each postprocess you run on the timestamp, until you change it.

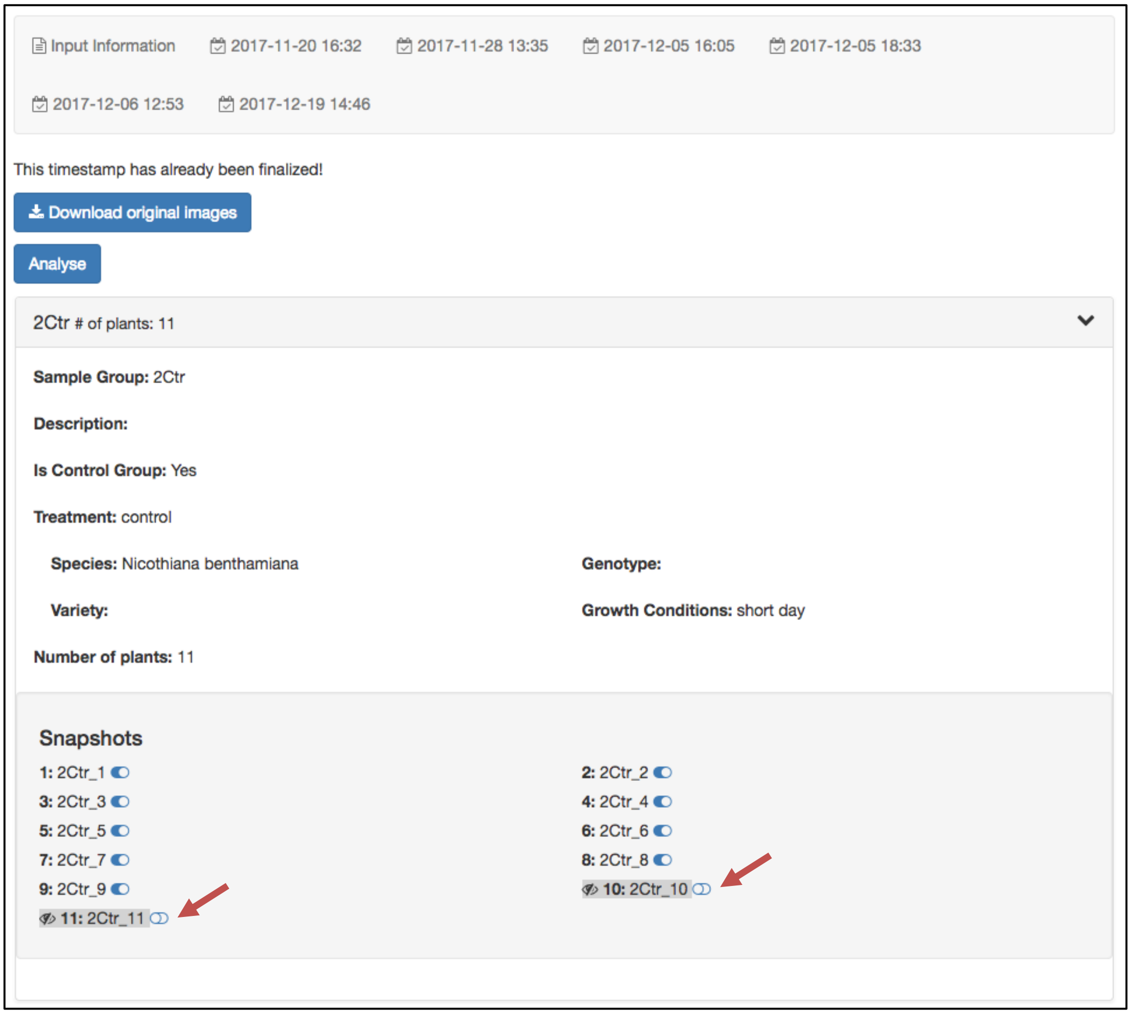

Running a Postprocess on existing IAP results

When you want to run a postprocess on an already existing IAP analysis, you have two options (A or B):

Variant A: Click on the 'Analyse' button when viewing a timestamp, as when you start a full analysis including IAP segmentation. Then check the 'Use Existing Result' box and select the appropriate entry. The entry name consists of the Pipeline name and the time at which the analysis was started.

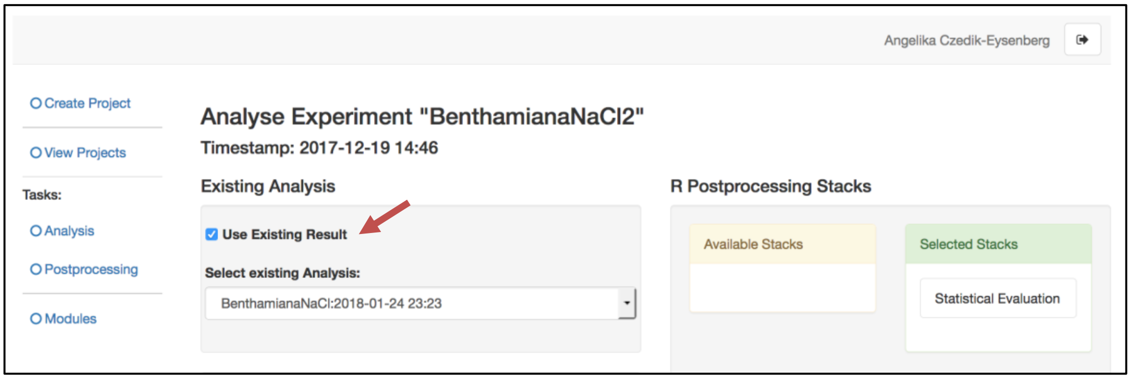

Variant B: Click on 'Apply Postprocessing stack' in the results detail view. This preselects the according IAP results for you.

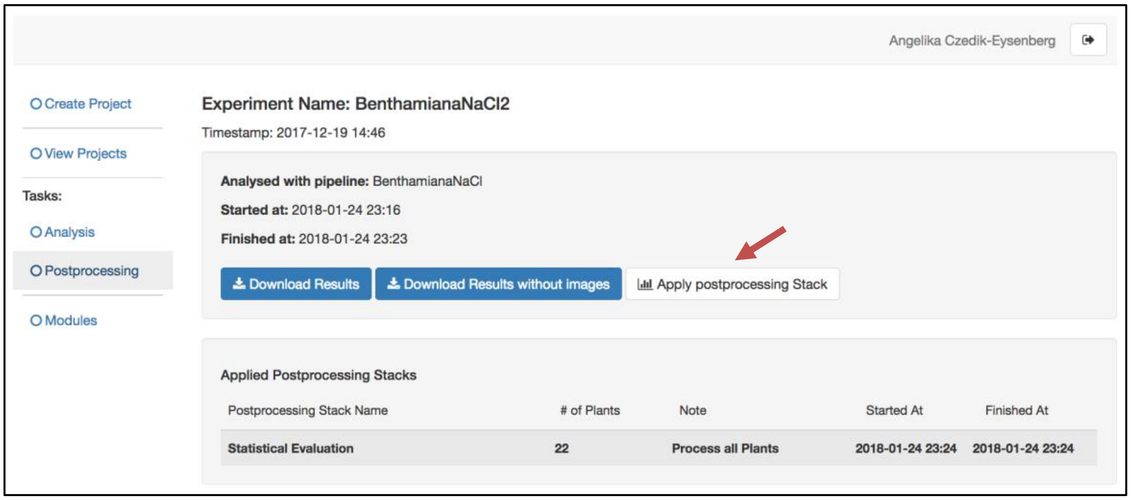

In both cases the list of postprocessing stacks only contains the ones you have not applied already to the selected results with the current snapshot exclusions.
